# Population history rather than tree age contributes to the evolutionary importance of ancient trees in an endangered conifer

**DOI:** 10.1038/s41477-026-02367-9

**Published:** 2026-08-11

**Authors:** Wei-Ping Zhang, Chao Feng, Xi-Zuo Shi, Shou-Jie Li, Martin Lascoux, Ming Kang

**Affiliations:** 1 State Key Laboratory of Plant Diversity and Specialty Crops, South China Botanical Garden, Chinese Academy of Sciences, Guangzhou, China; 2 Key Laboratory of National Forestry and Grassland Administration on Plant Conservation and Utilization in Southern China, Guangzhou, China; 3 Department of Ecology and Genetics, Evolutionary Biology Centre, Uppsala University, Uppsala, Sweden; 4 University of Chinese Academy of Sciences, Beijing, China

**Keywords:** Plant evolution, Population genetics, Conservation biology

## Abstract

Ancient trees are in global decline and face increasing conservation challenges. Their exceptional longevity has fostered the view that they are genetic reservoirs, yet whether old age is synonymous with unique genetic variation remains unclear. Here we assembled a ~8-Gb chromosome-level reference genome for the critically endangered conifer *Glyptostrobus pensilis*, now largely restricted to southern China with scattered populations in Vietnam and Laos, and resequenced 147 individuals, including 64 ancient (>100 years old and persisting in human-dominated landscapes), 33 wild and 50 recently cultivated individuals. Ancient individuals comprised both likely natural relics and historically introduced individuals and formed two deeply divergent lineages and one ancestral–admixed group, each with distinct demographic histories of prolonged contraction and genomic erosion. Lineage identity explained more variation in genome-wide diversity, inbreeding and genetic load than the three conservation types, despite broad differences in age structure. Rare-allele analyses revealed pronounced heterogeneity among ancient trees: only relic and ancestral-origin individuals from high-diversity lineages contributed substantial unique variation, much of which is poorly represented in wild and cultivated populations. Together, our findings suggest that ancient trees are not uniformly genetically irreplaceable and that, at least in this conifer, evolutionary importance is shaped more strongly by population history than by age alone.

## Abstract (web summary)

Genome analyses of the endangered conifer *Glyptostrobus pensilis* reveal that population history, rather than tree age, contributes more strongly to the evolutionary importance of ancient trees.

## Main

Ancient or old trees, long-lived individuals that persist for centuries or even millennia, are increasingly recognized as irreplaceable elements of natural forests and human-dominated landscapes. Their extreme longevity confers profound cultural value and allows them to integrate ecological and evolutionary processes across long timescales, forming living archives of past environments^1–6^. They can also function as structural anchors that support local biodiversity through the provision of persistent habitats and microhabitats for diverse organisms^3,7,8^. Given accelerating mortality and exposure to multiple interacting threats, ancient trees have become a prominent focus of conservation initiatives and are increasingly advocated as priorities in future biodiversity strategies^2,4,9,10^. In this context, previous studies have suggested that ancient trees or old-growth systems can retain important genetic diversity^11,12^. Whether this also means that ancient trees are themselves intrinsically valuable genetic reservoirs, however, remains insufficiently resolved.

This uncertainty is especially consequential for relict and endangered taxa^1^. Range contraction, habitat fragmentation and poor natural regeneration typically reduce genomic diversity while increasing inbreeding and genetic load, collectively resulting in genomic erosion with potentially negative consequences for long-term viability^13,14^. Within such systems, surviving ancient trees may retain remnants of ancestral diversity or allelic combinations underrepresented in contemporary populations that enhance adaptive capacity^2,15^, thereby providing disproportionately valuable material for conservation and restoration. Alternatively, they may represent genetically eroded survivors whose persistence masks underlying vulnerability, as genomic responses to environmental change can lag for many generations in long-lived species^16^. Distinguishing between these possibilities is essential for evidence-based conservation planning. More broadly, the evolutionary relevance of an ancient individual is best understood in the context of population history. Lineage persistence, demographic contractions and historical bottlenecks can shape genomic variation independently of tree age; in some cases, the surviving representatives of a lineage may simply happen to be old trees. This complexity is further compounded by the fact that ancient trees represent a functional rather than strictly biological category, and may include both naturally persisting relics and historically introduced individuals with distinct demographic histories and genetic backgrounds. In addition, size-based protection can fail to safeguard genuinely old individuals^17^. Whether ancient trees retain irreplaceable genetic variation therefore cannot be inferred from age, size or status alone, and is best evaluated through genomic tests.

**Fig. 1:**
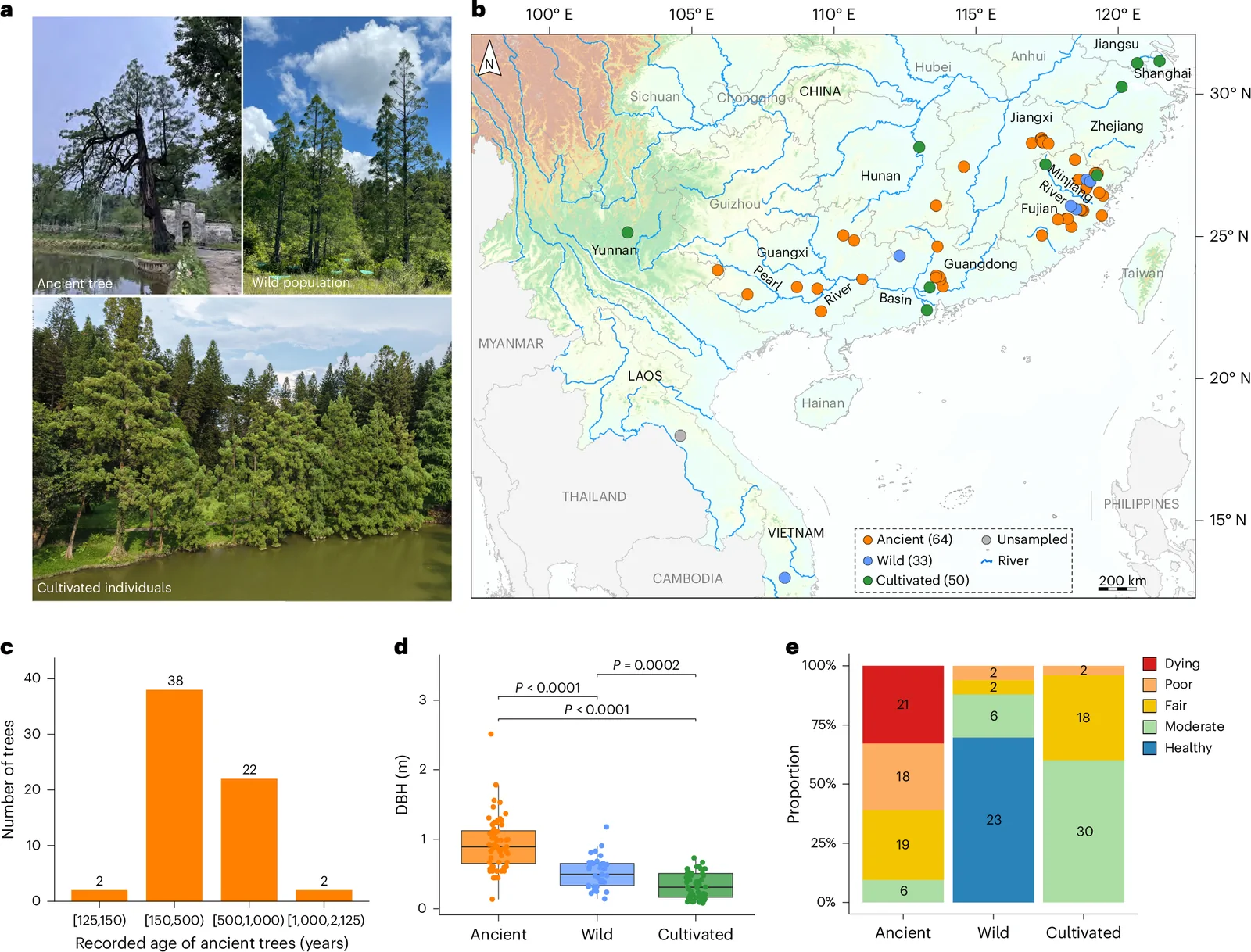
Conservation status, geographic distribution, recorded ages and phenotypic variation of *G. pensilis*. **a**, Representative photographs illustrating the three conservation types: ancient tree, wild population and cultivated individuals. **b**, Sampling sites of 147 individuals across southern China and Vietnam, including 64 ancient, 33 wild and 50 cultivated trees. Major river systems are shown, with most individuals distributed near the Pearl River (Zhujiang) basin and Minjiang River (Minjiang); a circle in Laos marks a known occurrence where no samples were obtained for this study. **c**, Distribution of recorded age classes for the 64 ancient trees. Recorded ages were compiled from published studies, on-site forestry protection plaques, news reports and local interviews, with detailed sources provided in Supplementary Table 3. **d**, Comparison of DBH among three conservation types. The smallest DBH value for ancient trees corresponds to a lateral branch of an individual with a dead main trunk. Box plots summarize the distribution across individuals: the centre line indicates the median, the box bounds correspond to the 25th and 75th percentiles, and whiskers extend to 1.5× the interquartile range. Statistical significance for pairwise group comparisons was assessed using two-sided Wilcoxon rank-sum tests, with *P* values adjusted using the Benjamini–Hochberg (BH) procedure. **e**, Stacked bar plot of five vitality categories (Dying, Poor, Fair, Moderate and Healthy) across the three conservation types. Source data

The critically endangered swamp cypress, *Glyptostrobus pensilis*^18^ (Fig. 1a), provides a practical and conservation-relevant system to address these questions. This wetland conifer is specialized to swamp and floodplain environments and usually forms distinctive pneumatophores above the water or soil surface^19^, making it highly sensitive to hydrological change. This relict lineage was widespread across the Northern Hemisphere from the Cretaceous to the early Eocene^20^ but is now restricted to southern China, Vietnam and Laos, where it persists mainly in remote inland areas and is rare in coastal regions^19,21–25^. Unlike many endangered taxa, its decline does not appear to have been driven directly by abrupt climatic shifts^26,27^. Instead, palaeoecological evidence suggests that repeated late-Holocene large fires (for example, ~2,100 years BP in the Pearl River Delta)^26^, together with more recent wetland degradation, anthropogenic deforestation and agricultural expansion^27^, probably contributed to progressive range contraction and fragmentation. Today, the species persists as ~100 geographically scattered ancient trees, alongside ~10 extremely small wild populations and several recently cultivated plantings established largely within the past five decades^21,22,25^. In this study, ancient trees refer to documented individuals older than 100 years occurring in human-dominated landscapes^28^, excluding those in remnant natural swamp habitats, and thus represent a conservation type distinct from wild and cultivated trees.

Here, we assembled and annotated a chromosome-level (~8 Gb) reference genome for *G. pensilis* and generated population-scale whole-genome resequencing data from 147 individuals spanning ancient, wild and cultivated trees across its fragmented range. These data allow us to assess how population history shapes the persistence of ancient individuals and to quantify the representation of their genetic variation in contemporary wild and cultivated populations. More specifically, in this endangered conifer, we ask whether the evolutionary importance of ancient trees is better explained by age-related status alone or by demographic lineage.

## Results

### Ancient trees differ from wild and cultivated trees in age structure and field condition

To characterize the contemporary status of *G. pensilis* across management contexts, we surveyed 59 sites across its distribution in southern China and Vietnam and sampled 147 living adult individuals for ecological and genomic analyses, representing three non-overlapping conservation types: 64 ancient, 33 wild and 50 cultivated trees (Fig. 1a,b and Supplementary Table 1). Most known surviving ancient trees were included, while wild and cultivated trees were collected representatively from documented populations and planting sites (Supplementary Table 2).

Available age-related information indicated broad differences in age structure among the three conservation types, despite the infeasibility of direct age measurement in this species. All sampled ancient trees had documented ages exceeding 100 years, and most recorded ages fell between 150 and 800 years after excluding the excluding the four most extreme values (Fig. 1c and Supplementary Table 3). For the wild type, 22 of the 33 individuals came from Fujian and 3 from Vietnam, the two main sampled wild-population regions of *G. pensilis*, for which published age data indicate mean ages of ~85 years^22^ and ~112 years^21^, respectively. The remaining eight wild individuals came from a small population in Guangdong, where even the largest reported tree is unlikely to exceed ~70 years. Cultivated trees were mostly recent plantings (~20–50 years). Diameter at breast height (DBH) differed in the same general direction among the three conservation types (median: 89.2 cm in ancient, 49.4 cm in wild and 31.1 cm in cultivated trees; Fig. 1d and Supplementary Table 1), providing supporting size-related evidence consistent with the age-related patterns summarized above.

**Extended Data Fig. 1:**
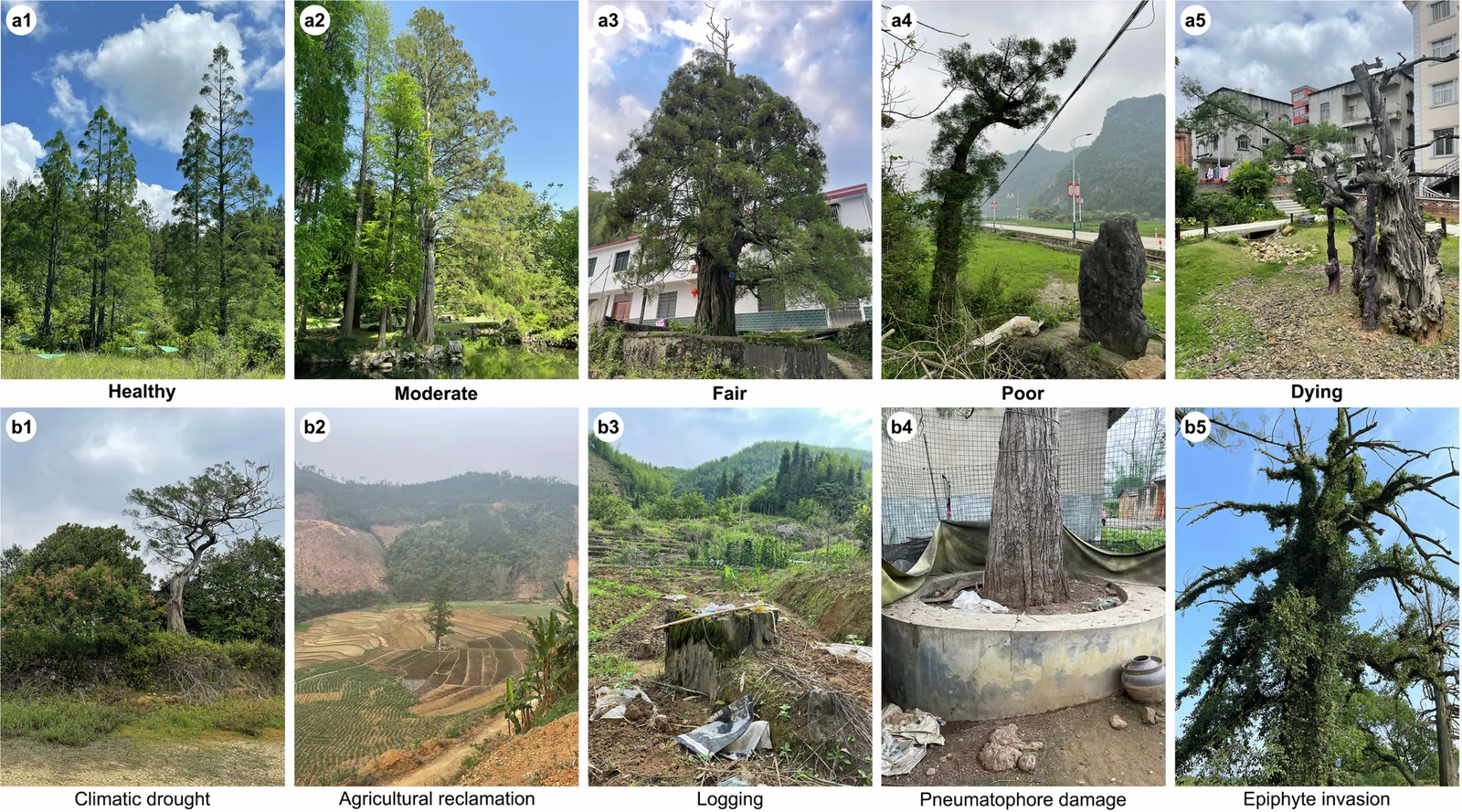
Vitality classification and major threat factors of sampled *G. pensilis* individuals based on field observations. **a** All 147 sampled individuals were assigned to one of five vitality categories, Healthy (**a1**), Moderate (**a2**), Fair (**a3**), Poor (**a4**), and Dying (**a5**), primarily based on crown structure, the abundance of newly produced leaves and cones, presence of seedlings beneath the canopy, pneumatophore development, surrounding habitat conditions, and epiphytic plant load. **b** Field surveys identified major threat factors affecting the growth and persistence of ancient individuals, including climatic drought (**b1**), agricultural reclamation (**b2**), logging and land clearing (**b3**), pneumatophore damage caused by burial or obstruction from artificial barriers (**b4**), and epiphyte invasion, exemplified by heavy infestation of *Ficus pumila* (**b5**).

Ancient trees were more frequently in poor condition than wild and cultivated trees, highlighting greater conservation urgency, based on current-condition categories assigned from field observations of crown architecture, structural integrity, growth condition, reproductive status and habitat quality (Methods and Supplementary Note 1). Specifically, 60.9% (39/64) of ancient trees were classified as Poor or Dying, whereas 87.8% (29/33) of wild individuals were Healthy or Moderate and 96% (48/50) of cultivated trees were Moderate or Fair (Fig. 1e, Extended Data Fig. 1 and Supplementary Table 1). Although natural regeneration was rarely observed in the field, seeds from 14 representative trees of all three conservation types germinated under common-garden conditions (Supplementary Fig. 1 and Supplementary Table 4), indicating that reproductive capacity was not completely lost.

### A high-quality reference genome for conservation genomics of *G. pensilis*

**Extended Data Fig. 2:**
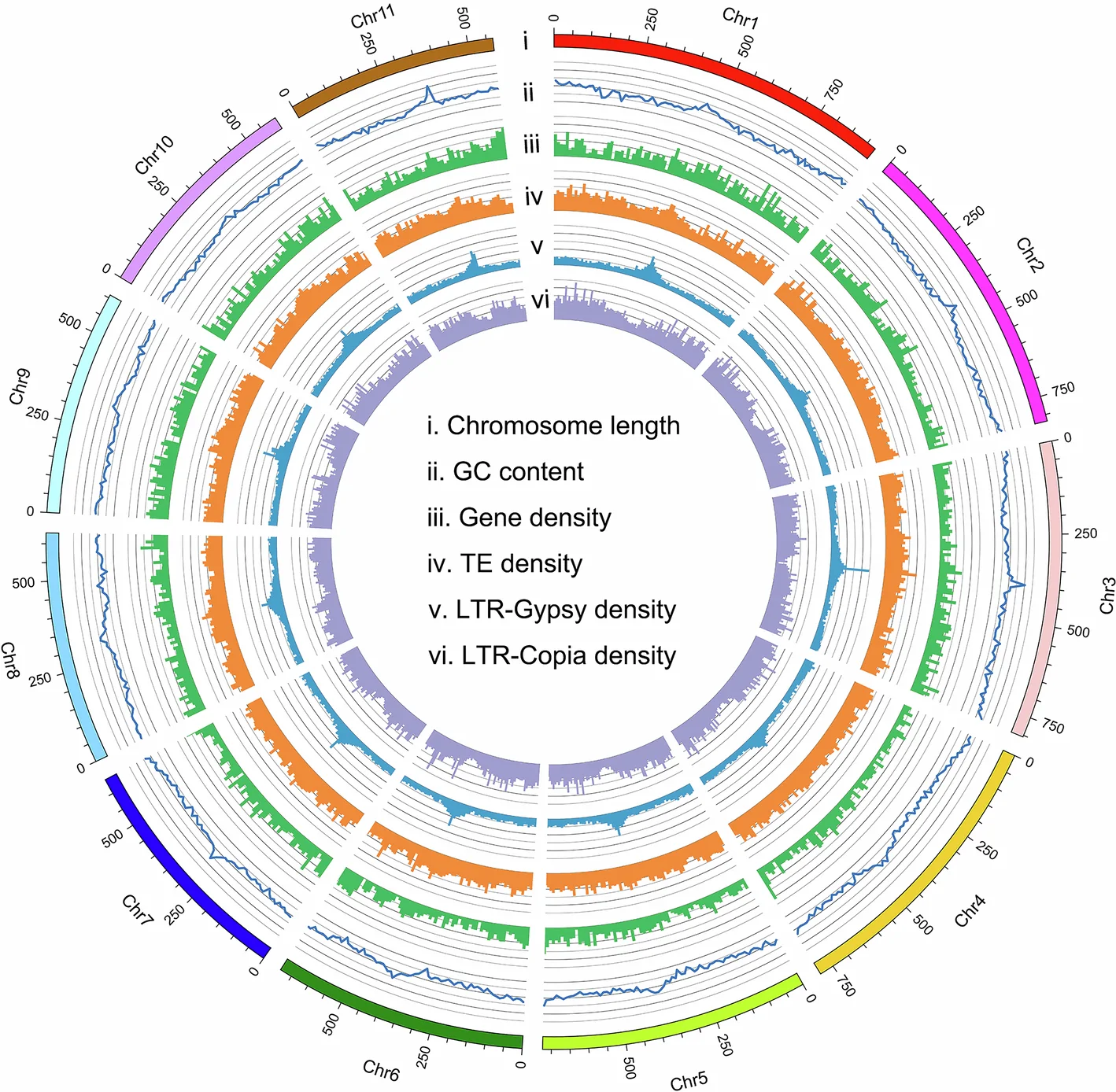
Genome features of *G. pensilis*. Circos plot showing major genomic features across 11 chromosomes. Tracks from the outside inward represent: (i) chromosome length (Mb); (ii) GC content; (iii) gene density; (iv) transposable element (TE) density; (v) LTR- Gypsy density; and (vi) LTR- Copia density. All features were calculated in 10-Mb non-overlapping windows. Source data

To provide a genomic foundation for subsequent conservation analyses, we assembled a chromosome-level reference genome of *G. pensilis* using Illumina, PacBio and Hi-C sequencing. In total, ~961.49 Gb of raw data (~121.3× coverage) were generated. *K*-mer analysis estimated a genome size of ~8.03 Gb with a heterozygosity rate of 0.24%. The final 7.93-Gb assembly was anchored to 11 pseudochromosomes, with a scaffold N50 of 723.2 Mb and a contig N50 of 1.5 Mb. Assembly quality was supported by 90.9% and 96.9% complete BUSCOs for embryophyta_odb10 and gymnosperm_odb10 datasets^29^, a 98.8% read-mapping rate and a long terminal repeat (LTR) assembly index (LAI) of 15.9, indicative of reference-grade quality. Repetitive elements accounted for 71.9% of the genome and were dominated by LTR retrotransposons (59.2%), consistent with the large genome size reported for closely related conifers (for example, *Metasequoia*^30^ and *Taxodium*^31^). We predicted 39,582 protein-coding genes, 64.4% of which were functionally annotated. Comparative analyses revealed no recent lineage-specific whole-genome duplication events and confirmed the placement of *G. pensilis* within the Cupressaceae. Expanded, contracted and species-specific gene families were enriched for water transport, root development, hypoxia and osmotic-stress responses, and cell-wall formation, broadly consistent with adaptation to swamp and periodically inundated habitats. Full details are provided in the Supplementary Information (Extended Data Fig. 2, Supplementary Figs. 2–6 and Supplementary Tables 5–11).

### Ancient trees belong to distinct genetic lineages shaped by long-term population history

**Fig. 2:**
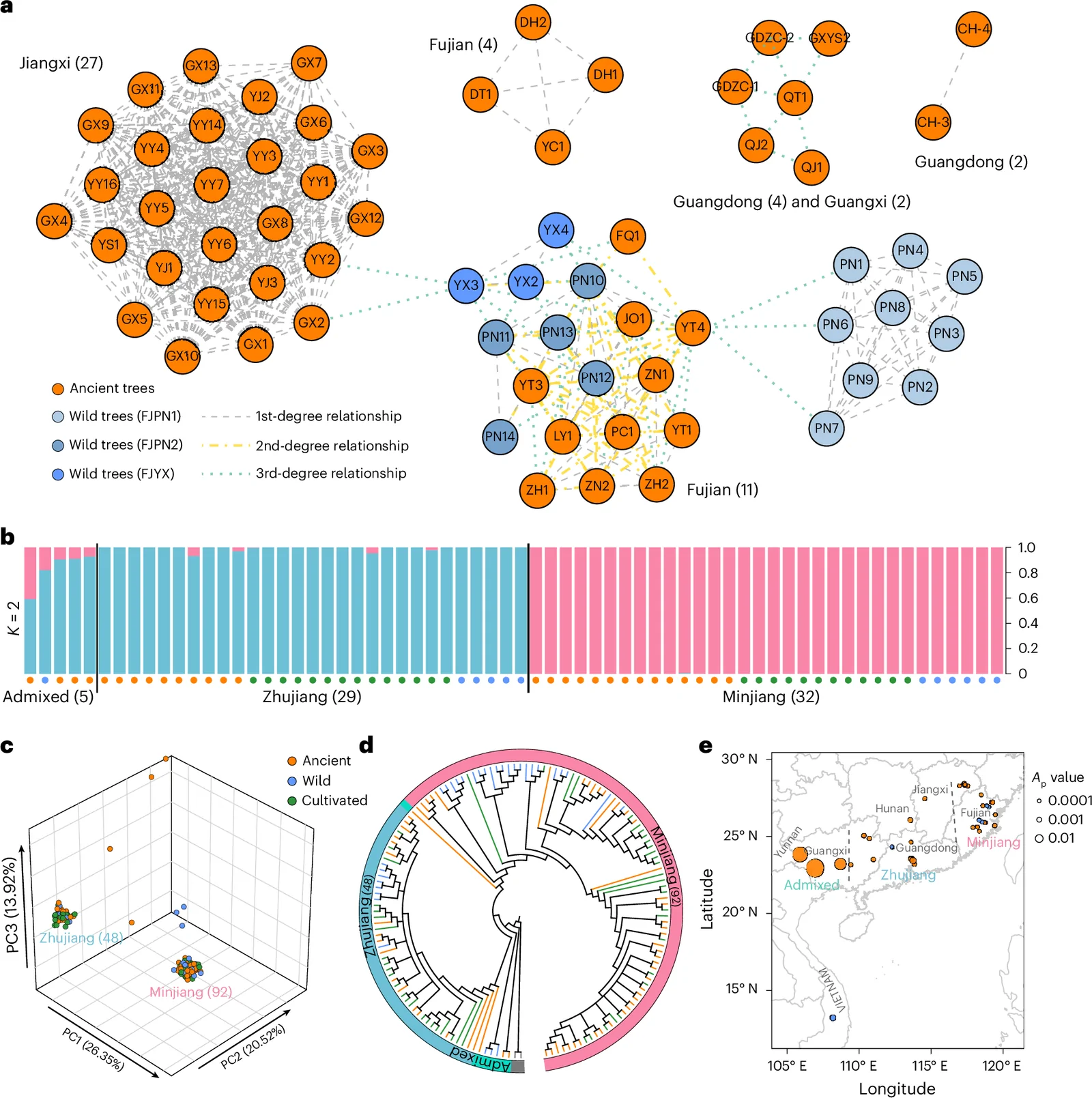
Genetic relatedness, geographic provenance and population structure. **a**, Pairwise kinship relationships among ancient and wild individuals, with first- to third-degree relatedness indicated by three types of dashed lines. Twenty-seven ancient trees from Jiangxi and eleven from Fujian Provinces, China, show first- to third-degree relatedness to three nearby wild populations. The remaining 26 ancient trees show no such pattern, although 12 were related to other ancient trees. **b**, Population structure inferred with STRUCTURE (*K* = 2) from 66 unrelated individuals after removing close kinship within three generations. Two major genetic lineages were identified: Zhujiang (29 individuals) and Minjiang (32), with five admixed individuals. **c**, PC-AiR of 147 individuals, with individuals assigned to Zhujiang (*n* = 48), Minjiang (*n* = 92) or admixed (*n* = 7). **d**, Phylogenetic relationships among 147 individuals inferred using SVDquartets and rooted with two outgroup taxa. **e**, Geographic distribution of private alleles (*A*_p_) in ancient and wild individuals. Cultivated individuals are excluded owing to potential recent translocations. Panels **b**–**e** share a common key indicating the three conservation types. Source data

Across the 147 resequenced individuals (mean depth 29.9×; Supplementary Table 12), the 64 ancient trees sampled across six provinces in southern China showed heterogeneous genetic origins reflecting their uneven geographic distribution (Fig. 1b and Supplementary Table 1). Eleven individuals from Fujian formed a first- to third-degree kinship cluster and were closely related to three nearby wild populations (Fig. 2a), suggesting historical introduction from local sources. Twenty-seven individuals from northeastern Jiangxi formed a single first-degree related cluster, most likely half-sibs, and were also closely related to the same nearby wild populations, consistent with a strong founder effect associated with human-mediated introduction. Together, these patterns identify 38 ancient trees as probably historically introduced. By contrast, the remaining 26 ancient trees showed little or no close kinship and lacked clear affinity to nearby extant wild populations (Fig. 2a and Supplementary Table 3) and are therefore inferred to represent likely natural relics. Cultivated trees formed clonal groups and were largely derived from a limited set of ancient sources, with additional evidence of exchange among planting sites, whereas wild populations showed pervasive first- to third-degree relatedness consistent with small, fragmented populations (Supplementary Fig. 7 and Supplementary Note 2).

To infer population structure independent of close kinship, we retained 66 unrelated individuals for clustering analyses. STRUCTURE analysis^32^ (optimal *K* = 2; Supplementary Table 13) resolved two strongly differentiated lineages: the Zhujiang lineage (*n* = 29), distributed across the Pearl River basin, and the Minjiang lineage (*n* = 32), centred near the inland Minjiang River (*F*_ST_ = 0.42, *d*_xy_ = 0.00273), along with a small number of admixed individuals (Fig. 2b). Principal component analysis (PCA) showed concordant separation (Supplementary Fig. 8). Analyses incorporating all 147 individuals using PCA in in related samples (PC-AiR)^33^, which accounts for both population structure and relatedness, together with phylogenetic analysis using SVDquartets^34^, supported the same structure, assigning 48 individuals to Zhujiang, 92 to Minjiang and 7 to an admixed group (Fig. 2c,d and Supplementary Table 12). Ancient trees occurred in both major lineages and the admixed group, indicating that they do not correspond to a single genetic entity. The seven admixed individuals, six of which originated from Guangxi, Yunnan and adjacent areas of Vietnam, occupied scattered or basal positions in PCA and the phylogenetic tree and carried markedly elevated private allele (*A*_p_) proportions (mean 3.81 × 10⁻^2^, compared with 6.42 × 10⁻^4^ in Zhujiang and 1.75 × 10⁻^4^ in Minjiang). Their rare-allele richness showed a clear southwest-to-northeast decline (Fig. 2e), indicating that the admixed group represents relict lineages retaining ancestral polymorphisms and suggesting Indochina/southwestern China as the historical distribution centre of *G. pensilis*. Aside from these admixed individuals, ancient and non-ancient wild individuals in the Zhujiang and Minjiang lineages showed broadly similar private-allele proportions.

**Fig. 3:**
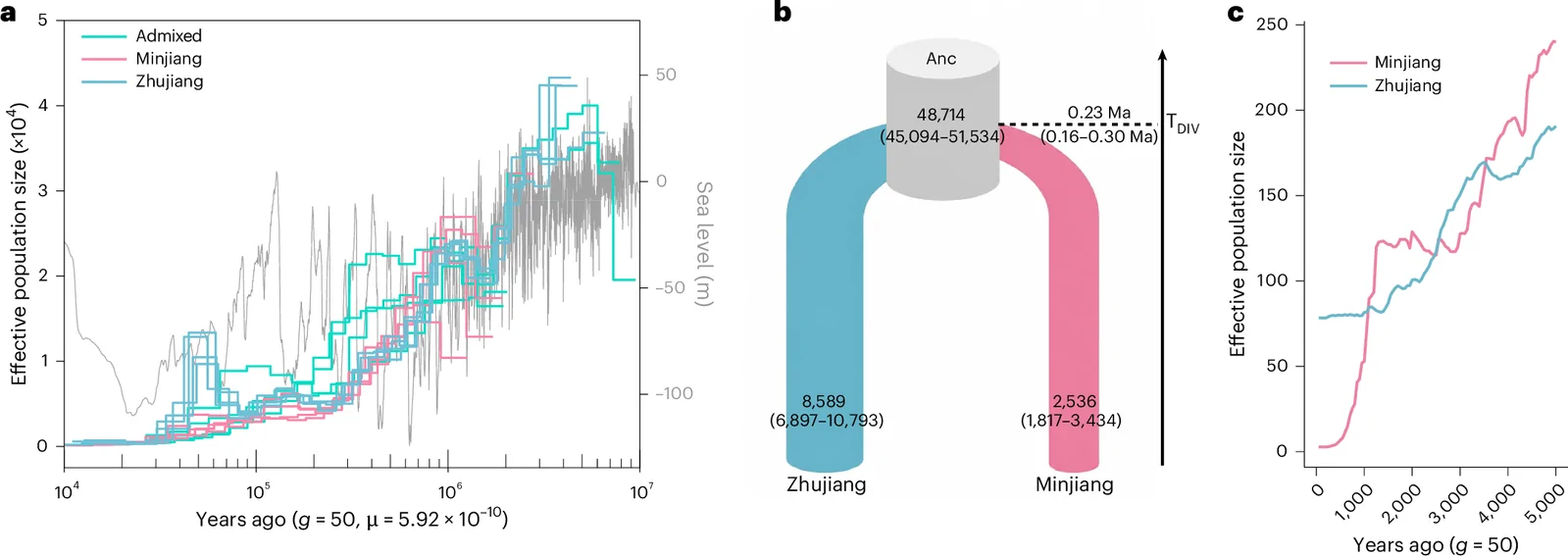
Long-term and recent demographic history inferred from whole-genome data. **a**, Long-term effective population size (*N*_e_) trajectory for the three genetic lineages inferred using the PSMC method. Four representative individuals with demographically informative *N*_e_ curves are shown for each lineage. Coloured lines represent inferred *N*_e_, and the grey curve indicates relative sea-level fluctuations^44,45^. The left *y* axis shows *N*_e_, and the right *y* axis shows relative sea level. **b**, Simplified schematic of the best-fitting demographic model for the Zhujiang and Minjiang lineages inferred using fastsimcoal2 based on a dataset of 61 unrelated individuals. Anc denotes the ancestral population and T_DIV_ denotes the estimated time of divergence between the Zhujiang and Minjiang lineages. Numbers indicate parameter estimates (effective population sizes or divergence times), with values in parentheses representing 95% confidence intervals. **c**, Recent effective population sizes (*N*_e_) for the Zhujiang (*n* = 29) and Minjiang (*n* = 32) lineages inferred using GONE. Generation time was set to 50 years, and a mutation rate of 2.96 × 10⁻^8^ substitutions per site per generation was assumed^74^. A recombination rate of 0.154 cM Mb^−1^ was applied based on estimates from the closely related conifer *C. japonica*^76,77^. Source data

To understand how the two divergent lineages and the ancestral–admixed group were shaped, we reconstructed their long-term and recent demographic histories. Pairwise sequentially Markovian coalescent (PSMC) analyses^35^ revealed pronounced lineage contrasts: Minjiang individuals generally exhibited shallow histories (~0.2–0.3 Ma), whereas most Zhujiang and ancestral–admixed individuals retained much deeper trajectories exceeding 1 Ma (Supplementary Fig. 9). All groups showed temporal patterns broadly coincident with Quaternary sea-level fluctuations under the assumed mutation-rate and generation-time parameters, including an early bottleneck and partial recovery during long-term sea-level decline (~5–1 Ma), followed by subsequent demographic shifts associated with intermediate sea-level oscillations (Fig. 3a). Effective population sizes (*N*_e_) fell to ~400–1,000, approximately corresponding to the period approaching the Last Glacial Maximum. From ~1 Ma onwards, lineage-specific trajectories emerged: both lineages underwent sustained declines, but Zhujiang showed a modest late-Pleistocene rebound, whereas Minjiang continued to contract without recovery. During the same interval, the ancestral–admixed group retained higher *N*_e_ and a slower decline, with *N*_e_ converging towards similarly low levels over the more recent part of the trajectory. Coalescent modelling with fastsimcoal2^36^ supported a strict isolation model and dated the split between the two major lineages to ~0.23 Ma (late Quaternary; 95% confidence intervals 0.16–0.30 Ma) without gene flow (Fig. 3b, Supplementary Fig. 10 and Supplementary Table 14). The demographic model also inferred strongly asymmetric population contractions (~5.8-fold in Zhujiang versus ~21.3-fold in Minjiang), consistent with their contrasting long-term *N*_e_ profiles. Finally, linkage disequilibrium (LD)-based inference using GONE^37^ revealed a shared *N*_e_ decline over the past ~100 generations, falling from roughly 240 to 80, with Minjiang showing an additional sharp collapse within the last 15 generations (Fig. 3c).

### Genomic diversity and genetic load reflect lineage history more strongly than conservation type

**Fig. 4:**
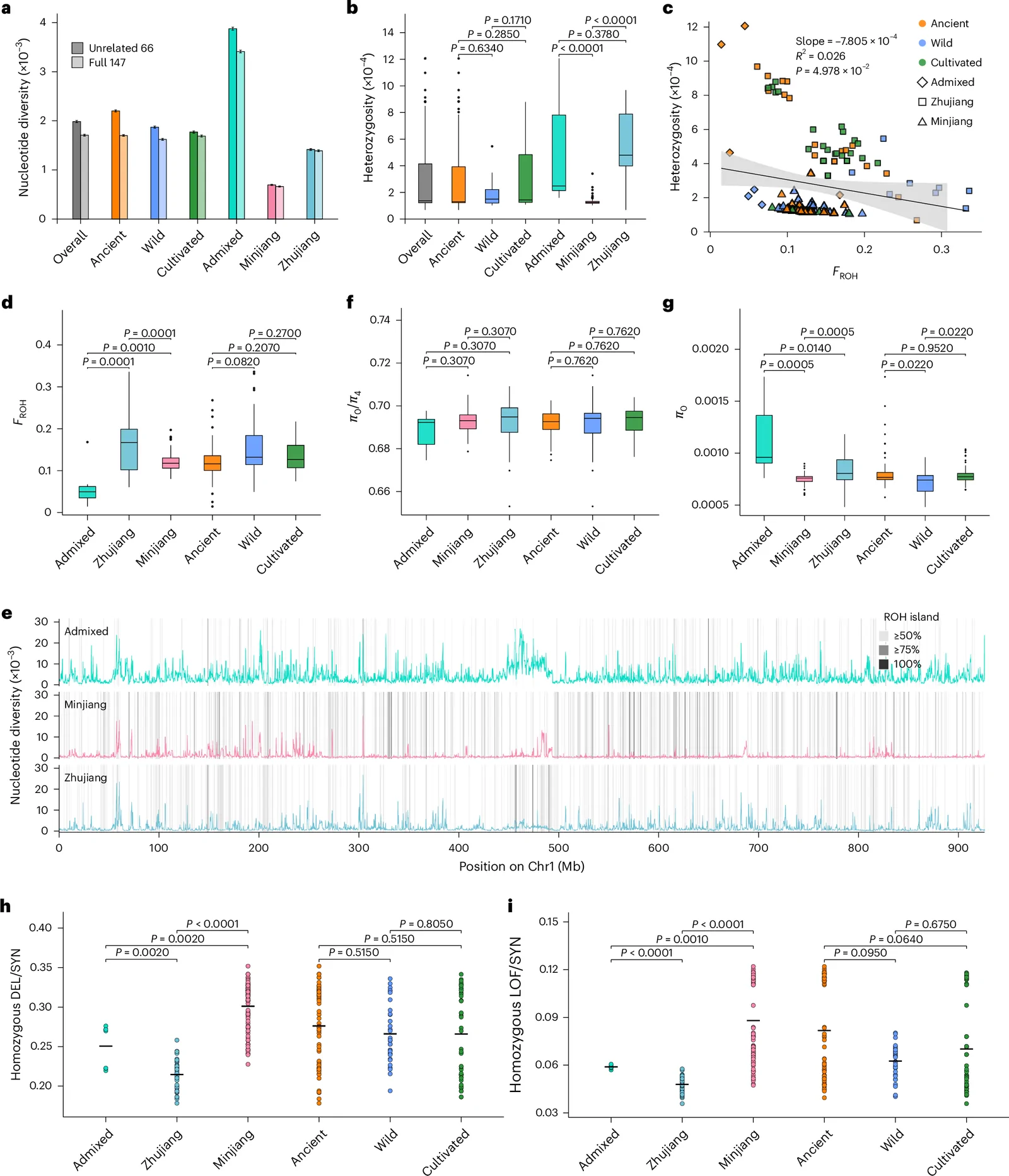
Genomic diversity, inbreeding and genetic load across genetic lineages and conservation types. **a**, Genome-wide nucleotide diversity (*π*) summarized overall and for each conservation type and genetic lineage. Bars represent *π* estimates based on the unrelated dataset (*n* = 66) and the full dataset (*n* = 147). Error bars indicate bootstrap 95% confidence intervals for the *π* estimates, calculated from 200-kb windows (1,000 bootstrap replicates). **b**, Mean genome-wide heterozygosity summarized overall and separately for the three conservation types and three genetic lineages. **c**, Relationship between individual-level genome-wide heterozygosity and *F*_ROH_, defined as the proportion of the genome contained within ROH. **d**, Comparison of *F*_ROH_. **e**, ROH islands along chromosome 1 (Chr1) for the three genetic lineages, shown together with nucleotide diversity (*π*) in 200-kb sliding windows. Colour intensity indicates regions shared by >50%, >75% and 100% of individuals within each lineage. **f**,**g**, Heterozygosity at 0-fold relative to 4-fold degenerate sites (*π*_0_/*π*_4_; **f**) and at non-synonymous (0-fold degenerate) sites (*π*_0_; **g**). **h**,**i**, Ratios of homozygous derived deleterious variants (**h**) and loss-of-function variants (**i**) to homozygous derived synonymous variants. DEL, LOF and SYN denote deleterious, loss-of-function and synonymous variants, respectively. Horizontal bars denote average values. All analyses in **b**–**i** are based on the full 147-individual SNP dataset; comparative analyses are shown across the three genetic lineages and three conservation types. Box plots summarize the distribution across individuals: the centre line indicates the median, the box bounds correspond to the 25th and 75th percentiles, and whiskers extend to 1.5× the interquartile range. Statistical significance for pairwise group comparisons was assessed using two-sided Wilcoxon rank-sum tests, with *P* values adjusted using the Benjamini–Hochberg (BH) procedure. Source data

Using the 66 unrelated individuals (ancient, *n* = 28; wild, *n* = 12; cultivated, *n* = 26), overall nucleotide diversity was *π* = 1.99 × 10⁻^3^ and differed only modestly among tree types: ancient (2.20 × 10⁻^3^), wild (1.87 × 10⁻^3^) and cultivated (1.77 × 10⁻^3^) (Fig. 4a). By contrast, lineage-level differences were much stronger: the ancestral–admixed group showed the highest diversity (*π* = 3.88 × 10⁻^3^), Zhujiang was intermediate (1.42 × 10⁻^3^) and Minjiang was substantially lower (6.96 × 10⁻^4^). These contrasts remained when all 147 individuals were analysed, although absolute *π* values were slightly reduced due to kinship among samples. Genome-wide heterozygosity showed the same patterns, with significantly higher values in the ancestral–admixed and Zhujiang groups than in Minjiang, and no significant differences among conservation types (Fig. 4b). This weak type-level separation reflects the substantial mixture of lineage backgrounds within each type.

Genome-wide runs of homozygosity (ROH) across 147 individuals showed that long tracts were rare (>1 Mb; the longest was 1.615 Mb, representing only 0.014% of all ROH; Supplementary Table 15), indicating little evidence for extremely recent inbreeding. Consistently, the genomic proportion of ROH (*F*_ROH_) was only weakly negatively correlated with heterozygosity (slope = –7.8 × 10⁻^4^, *R*^2^ = 0.026; Fig. 4c), suggesting that observed ROH variation primarily reflects long-term demographic processes. Clear differences emerged among genetic lineages. Zhujiang individuals had a higher overall ROH burden, with longer and more numerous ROH segments across all four size classes (Methods, Fig. 4d and Supplementary Fig. 11). By contrast, Minjiang individuals, although carrying fewer long tracts, exhibited substantially larger fractions of ROH islands shared among individuals (Fig. 4d,e and Supplementary Fig. 12), suggesting older and more pervasive inbreeding. The ancestral–admixed group showed the lowest overall ROH burden, in line with its elevated genome-wide diversity. Inbreeding coefficients (*F*_IS_), reflecting historical erosion of heterozygosity, showed a similar lineage ranking, rising from the ancestral–admixed group (0.239) to Zhujiang (0.391) and reaching the highest values in Minjiang (0.613), with Minjiang significantly exceeding the other two groups (Supplementary Fig. 13). By contrast, differences among conservation types were comparatively modest: ancient, wild and cultivated trees showed broadly overlapping ROH profiles (Fig. 4d) and similar mean *F*_IS_ values (0.504, 0.606 and 0.492, respectively).

We further calculated the genome-wide ratio of non-synonymous (0-fold) to synonymous (4-fold) diversity, which was uniformly very high across all 147 individuals (*π*_0_/*π*_4_ = 0.65–0.71, towards the upper end reported for plants; Fig. 4f), indicating globally weak purifying selection at the species level. Both *π*_0_ and *π*_4_ declined markedly from the ancestral–admixed group to Zhujiang and then to Minjiang (Fig. 4g and Supplementary Fig. 14), mirroring their long-term *N*_e_ differences, whereas *π*_0_/*π*_4_ did not differ among lineages. Notably, the ratio of derived deleterious missense to derived synonymous variants (DEL/SYN) ranged from ~0.28 to 0.47, and the ratio of derived loss-of-function to derived synonymous variants (LOF/SYN) from ~0.08 to 0.15 (Supplementary Table 16), showing that a substantial fraction of segregating derived mutations is potentially harmful at the species level. Lineage-specific differences were clearer: Minjiang individuals carried the highest deleterious-load ratios in both heterozygous and homozygous states, whereas Zhujiang and the ancestral–admixed group exhibited comparably lower values (Fig. 4h,i and Supplementary Fig. 15), suggesting stronger drift and reduced purifying selection efficacy in the Minjiang lineage. Differences among conservation types were limited, with ancient and cultivated trees showing slightly higher *π*_0_ and *π*_4_ diversity and marginally elevated deleterious-load ratios relative to wild individuals (Fig. 4f–i and Supplementary Figs. 14 and 15).

Weak differentiation among conservation types was also observed within lineages. In the Minjiang lineage, ancient, wild and cultivated individuals showed broadly overlapping nucleotide diversity, heterozygosity, inbreeding (*F*_ROH_), selection efficacy (*π*_0_/*π*_4_) and mutation load metrics, without a consistent pattern of significant differences across these variables (Supplementary Fig. 16). A similar genomic pattern was observed in the Zhujiang lineage, with minor deviations involving wild individuals (Supplementary Fig. 17), probably reflecting their small sample size and origin from a single geographic population.

### Ancient trees differ in rare-allele representation across lineages and relic statuses

**Fig. 5:**
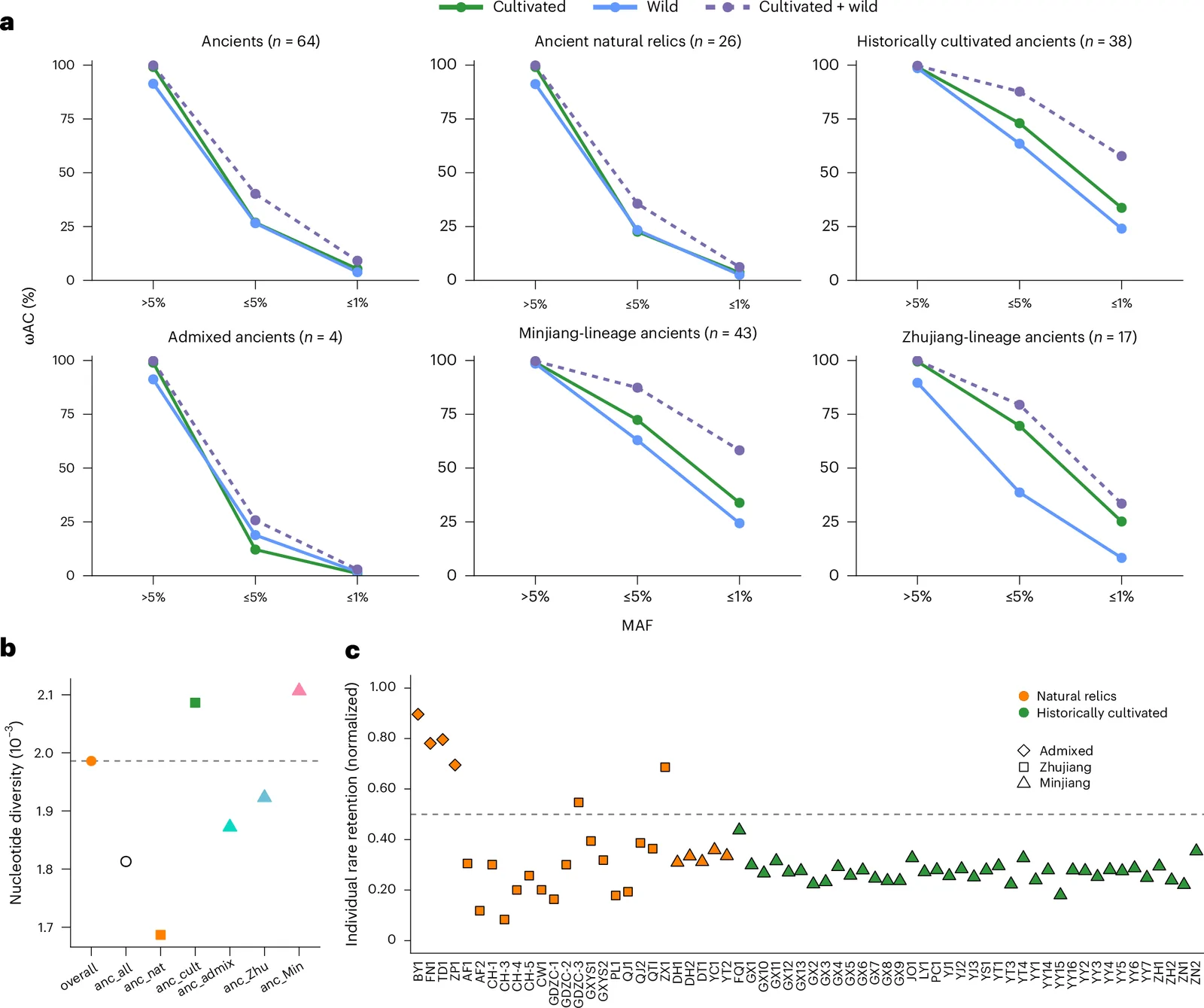
Representation and irreplaceability of ancient-tree genetic variation. **a**, ωAC of ancient-tree alleles across three MAF thresholds, showing the proportion of variants captured by cultivated, wild and combined (cultivated + wild) populations, based on the full dataset (*n* = 147). The full ancient-tree set comprised 64 individuals. For stratified analyses, these ancient individuals were grouped by origin (natural relics, *n* = 26; historically cultivated, *n* = 38), admixed group (*n* = 4) and genetic lineage (Minjiang, *n* = 43; Zhujiang, *n* = 17). **b**, Rarefaction analysis showing changes in nucleotide diversity (*π*) following the hypothetical loss of different ancient-tree subsets, based on the unrelated dataset (*n* = 66). Scenarios include: overall (no loss); anc_all (loss of all ancient trees); anc_nat (loss of natural relic ancient trees); anc_cult (loss of historically cultivated ancient trees); anc_admix (loss of admixed ancient trees); anc_Zhu (loss of Zhujiang-lineage ancient trees); and anc_Min (loss of Minjiang-lineage ancient trees). **c**, IRR (normalized to 0–1) for all ancient trees (*n* = 64), calculated relative to the combined cultivated and wild populations using the full dataset (*n* = 147). Higher IRR values indicate rare alleles present in ancient trees that are poorly represented in non-ancient populations. Source data

Ancient trees are often assumed to function as reservoirs of genetic diversity^11,12^, yet many *G. pensilis* ancient individuals are at imminent risk of loss. We therefore quantified how allelic variation from ancient trees is represented in contemporary wild and cultivated populations using rarity-weighted allelic coverage (ωAC) as the primary metric. For common variants (minor allele frequency (MAF) >0.05), wild populations, cultivated populations and their combined populations all captured >95% of ancient-tree alleles (Fig. 5a and Supplementary Fig. 18). However, coverage of rare alleles dropped steeply and differed strongly among lineages and between natural relic and historically introduced ancient trees. Rare-allele representation was lowest in ancestral–admixed ancient trees, intermediate in Zhujiang and highest in Minjiang, with coverage of 25.8%, 79.4% and 87.4% for low-frequency variants (MAF ≤0.05), and 2.9%, 33.5% and 58.3% for very rare variants (MAF ≤0.01; Fig. 5a). Origin-based differences were similarly pronounced: natural relic ancient trees had lower coverage than historically introduced ancient trees, with 35.6% versus 87.7% for MAF ≤0.05 and 6.2% versus 57.7% for MAF ≤0.01 in the combined wild and cultivated population. Across ancient-tree subsets, cultivated collections generally captured slightly more rare alleles than wild populations, except for the ancestral–admixed group, where wild individuals performed marginally better. In addition, rare alleles present in wild populations were poorly represented in cultivation (MAF ≤0.05: 42.0%; MAF ≤0.01: 19.3%; Supplementary Fig. 19), indicating that current ex situ collections incompletely capture natural genetic variation.

Rarefaction analyses based on the 66 unrelated individuals further clarified the genetic consequences of ancient-tree mortality across different categories. Simulated loss of relic trees, or of ancestral–admixed and Zhujiang ancient trees, caused disproportionately large reductions in nucleotide diversity, with decreases of 3.5–15.1% (Fig. 5b). By contrast, loss of historically introduced or Minjiang ancient trees resulted in slight diversity gains, indicating that many individuals in these groups contribute little unique variation to the overall gene pool.

To evaluate the genetic uniqueness of individual ancient trees, we quantified retention of rare ancient-specific alleles using the individual rare retention (IRR) index. IRR varied widely among the 64 ancient trees, ranging from 0.08 to 0.90 when assessed against the combined wild and cultivated population (Fig. 5c and Supplementary Fig. 20). Notably, individuals with IRR values above 0.5 were exclusively relic, ancestral–admixed or Zhujiang ancient trees, whereas Minjiang and historically introduced ancient trees consistently carried fewer unique alleles.

## Discussion

Ancient or old trees worldwide are experiencing accelerated mortality at an unprecedented rate, raising deep concerns about the irreversible loss of biological, ecological, cultural and genetic values^9,38^. Previous studies have primarily examined ancient trees from ecological and demographic perspectives, emphasizing extreme longevity, functional and physiological traits, as well as the influence of human land use and culture on their persistence and spatial distribution^2,3,5,8,17,28,39–42^. However, ancient trees are rarely incorporated into population genetic sampling designs^4^, leaving their population-level genomic value largely unexplored. Here, we address this gap by integrating a chromosome-level reference genome with whole-genome resequencing of 147 ancient, wild and cultivated individuals of the endangered conifer *G. pensilis*, enabling a direct population-genomic evaluation of whether ancient trees consistently retain evolutionarily important genetic variation.

Genome-wide patterns in *G. pensilis* indicate pervasive genomic erosion associated with a prolonged history of small effective population size. The species exhibits low nucleotide diversity (*π* = 1.99 × 10⁻^3^), comparable to that of other threatened gymnosperms such as *Cupressus gigantea* (2.01 × 10⁻^3^)^14^ and *Ginkgo biloba* (2.11 × 10⁻^3^)^43^. Such long-term demographic constraint is plausibly linked to Quaternary sea-level fluctuations^44,45^ and more recent human disturbance^26,27^, which probably maintained small, spatially fragmented populations over extended timescales.

Across the species, lineage history explained more of the observed genomic variation than conservation type, despite broad differences in age structure among ancient, wild and cultivated trees. Even without considering the ancestral–admixed group, the two major lineages still showed a consistent genomic contrast: Zhujiang retained higher genetic diversity and lower inbreeding and genetic load, whereas Minjiang showed the opposite pattern, together with more extensive sharing of ROH islands. Notably, Zhujiang individuals combine relatively high overall diversity with elevated ROH, consistent with temporally lagged genomic signals in long-lived species^16^. By comparison, differences among three conservation types were comparatively weak, both overall and within lineages, despite substantial heterogeneity among ancient trees. This lineage-specific context makes the genetic contribution of ancient trees highly uneven, reflecting redundancy versus uniqueness. Many Minjiang ancient trees are closely related and historically introduced, and their loss would have limited impact on nucleotide diversity estimates. Conversely, the loss of natural relic individuals from the ancestral–admixed and Zhujiang lineages could reduce overall diversity by more than 15%. Thus, genetic irreplaceability in this endangered conifer is determined less by age per se than by the lineage history and provenance that ancient trees represent, in line with lineage- and complementarity-based conservation theory^46–48^.

These lineage-specific genomic patterns have direct implications for the conservation of ancient trees in *G. pensilis*. Our results suggest that conservation strategies may benefit from prioritizing high-diversity, low-load lineages and their natural relic individuals, while maintaining representation of the highly eroded Minjiang lineage under ecologically appropriate conditions. Current ex situ collections capture only a limited proportion of rare genetic variation present in ancient trees, largely because cultivated material is often sourced from few individuals, inflating apparent census size while failing to represent rare alleles^49,50^. Such practices, together with reciprocal exchange and clonal propagation among botanical gardens (Supplementary Fig. 7), may further amplify genetically eroded lineages in managed landscapes, with potential consequences for adaptive capacity^51^.

Beyond these immediate management implications, our study adds a complementary, in some respects corrective, genomic perspective to research on ancient trees and their conservation. This is particularly relevant for endangered species shaped by strong demographic contraction, drift, and the frequent occurrence of ancient trees in human-dominated settings. Ancient individuals are often portrayed as life-history lottery winners^2^, with modular growth and tissue turnover proposed as mechanisms that may partially limit the accumulation of deleterious somatic mutations^40^ and thereby facilitate persistence across multiple generations of environmental variability. However, such within-individual processes do not necessarily reduce heritable genetic load at the population level, as our results demonstrate. Instead, the persistence of some ancient trees may reflect extrinsic factors, including habitat refugia^15,43^, cultural protection such as monumental, fengshui or worship trees^28,38,40^, or historical contingency under human management^40^, rather than superior genomic integrity. These findings caution against treating ancient trees as a genetically homogeneous or uniformly high-value category. In line with this view, recent surveys indicate that ancient-tree assemblages can be highly skewed, with a small number of human-associated, non-threatened species accounting for most ancient individuals, while many native and already rare species persist as only a handful or even single ancient trees^28^. This structural imbalance implies that the abundance of ancient trees within a species does not necessarily correspond to high genetic resilience or conservation security.

Overall, by integrating demographic history and rarity-weighted allele representation, our study shows that much of the heterogeneity among ancient trees is better explained by population history than by age alone. Similar logic may also extend beyond highly endangered taxa to more widespread species, because larger effective population sizes do not necessarily eliminate lineage structure^52^, and where more extensive regeneration and gene flow may allow genetic variation carried by old trees to be more readily transmitted to and retained in extant populations. The generality of this possibility, however, will require direct testing. At least in *G. pensilis*, longevity alone does not necessarily predict evolutionary importance; conservation efforts should focus more on preserving the genetic legacy of ancient trees that represent distinct lineages and contribute to the adaptive potential of subsequent populations.

## Methods

### Field surveys, conservation-type classification and age-related information

Between 2023 and 2024, we conducted comprehensive field surveys across the extant distribution of *G. pensilis* in southern China and northern Vietnam. Known occurrences in Laos were not sampled in this study owing to infeasible field access. All sampled trees were classified into one of three non-overlapping conservation types (ancient, wild and cultivated; Fig. 1a) on the basis of ecological context, management history and site characteristics.

Ancient trees were defined as individuals older than 100 years occurring in human-dominated environments, such as traditional villages, temple ponds and agricultural landscapes, while excluding trees persisting in remnant natural swamp habitats. Wild trees occurred in remnant submontane swamp habitats at 459–1,254 m elevation, where hydrological and ecological conditions remain close to their natural state. Cultivated trees represented recent human plantings located in managed wetlands or other artificial settings such as botanical gardens, parks and campuses. Because ancient trees are scarce, sampling of this type was targeted to include most known surviving ancient trees where sampling was feasible, whereas interval-based random sampling was applied to wild and cultivated trees at the same sites to ensure comparable spatial coverage (Supplementary Tables 1 and 2). In total, 59 sites were surveyed, and 147 living adult individuals were sampled, including 64 ancient, 33 wild and 50 cultivated trees.

Age-related information was compiled for all sampled trees using the most reliable sources available for each conservation type. Given the Critically Endangered (CR) status of *G. pensilis*^18^, large-scale coring and ring counting were not carried out, as direct age determination was not feasible and could have posed unacceptable risks to tree health. For ancient trees, documented age information was compiled from multiple sources, with priority given to direct dendrochronological evidence from published studies where available, followed by forestry bureau records (including plaques and official news reports), local gazetteers and other archival documentation, and finally local interviews conducted during field investigation (Supplementary Table 3). Available records indicate that all sampled ancient trees were older than 100 years. We did not further subdivide ancient trees into narrower age classes because the available age records were derived from heterogeneous documentary sources and did not provide equally precise ages for all individuals. For wild trees, age-related information was obtained from published records for the main sampled wild-population regions in Fujian^22^ and Vietnam^21^, together with available news reports for the small Guangdong population. For cultivated trees, age was inferred primarily from planting histories, local records, and information provided by site managers during field investigation. These sources indicated that most cultivated individuals were recent plantings (~20–50 years old). DBH was recorded for all individuals and treated as supporting size-related information consistent with broad age-structure differences among conservation types, rather than as a direct estimator of chronological age for individual trees.

### Vitality assessment across three conservation types

For each of the 147 surveyed trees, we recorded geographic coordinates, elevation, habitat type, hydrological status, land-use context and protection information. Additional field observations included local stand pattern, crown condition, pneumatophore development, epiphyte load and fruiting status. The presence or absence of natural regeneration (seedlings or saplings) within a 10-m radius of each focal tree was also documented. Tree vitality was evaluated using a multi-indicator assessment based on crown architecture, structural integrity, growth condition, reproductive status and habitat quality. Anthropogenic disturbance was recorded separately as a site-related descriptor, but was not included as a direct component of the score. Individuals were assigned to one of five vitality categories (Healthy, Moderate, Fair, Poor and Dying) according to a consistently applied set of field criteria (Extended Data Fig. 1 and Supplementary Note 1). In addition, seed germination was evaluated using a common-garden experiment with mature cones collected from three representative conservation sites, with detailed procedures provided in Supplementary Note 3.

### Genome sequencing, assembly and annotation

Fresh leaf tissues of *G. pensilis* were collected from an adult tree (accession SCBG00004966) at the Relic Plant Garden of the South China National Botanical Garden (23.18746° N, 113.36625° E). Samples were snap-frozen in liquid nitrogen and processed by Novogene for genomic DNA extraction and library preparation. Three sequencing libraries were constructed: a short-insert (350 bp) Illumina NovaSeq 6000 platform (PE150) library, yielding ~251.05 Gb of clean reads; a long-insert (10–20 kb) PacBio Sequel II library, generating ~288.67 Gb of high-fidelity HiFi reads through Circular Consensus Sequencing; and a DpnII-based Hi-C library sequenced on the Illumina NovaSeq 6000 platform (PE150), producing ~421.77 Gb of high-quality data. Genome size, heterozygosity and repeat content were estimated from 17-mer frequency analysis of Illumina short reads using GCE v1.0.2^53^. A de novo assembly was generated from PacBio HiFi reads using hifiasm v0.16^54^, followed by the integration of Hi-C data with the ALLHiC pipeline^55^ to correct misjoins and anchor contigs to chromosomes. Assembly quality was evaluated using BUSCO v5.4.4^56^ against both the embryophyta_odb10 and gymnosperm_odb10 datasets^29^, as well as by Illumina read mapping and LAI assessment computed with LTR_retriever v2.9.0^57^. Genome annotation, including repeat annotation, gene prediction and functional annotation, was performed using an integrative pipeline combining homology, transcriptome, and protein evidence^58–60^. Detailed annotation procedures, synteny analysis and gene family analyses are described in Supplementary Notes 4 and 5.

### Resequencing read mapping and variant calling

Genomic DNA was extracted from 147 dried leaf samples and sequenced by BGI Genomics on the DNBSEQ-T7 platform (MGI Tech), targeting an average depth of ~20× (~160 Gb per individual) with 350-bp insert libraries and 150-bp paired-end reads. To facilitate comparative analyses in phylogenomics and genetic load estimation, one individual each of *Taxodium distichum* and *Cryptomeria japonica* was also resequenced. Before read mapping, adapter trimming and quality filtering were performed using Trimmomatic v0.32^61^ to remove low-quality bases (Phred <10), trim sliding windows (3 bp, average Phred <20) and discard reads shorter than 50 bp. High-quality reads were then aligned to the *G. pensilis* reference genome (after excluding scaffolds shorter than 10 Mb) using the BWA-MEM algorithm implemented in BWA v0.7.12^62^. Only uniquely mapped reads were retained. SAM files were converted to BAM, sorted with SAMtools v0.1.19^63^, and PCR duplicates were removed using the MarkDuplicates function in GATK v4.0^64^. Local realignment around indels was performed to correct misalignments and improve mapping accuracy. Variant calling was conducted per individual with the HaplotypeCaller module in GATK v4.0^64^, followed by joint genotyping using CombineGVCFs and GenotypeGVCFs.

To meet the requirements of different downstream analyses, we generated four single-nucleotide polymorphism (SNP) datasets under standardized filtering criteria, including minimum depth 5×, maximum depth 100× and retention of biallelic sites using VCFtools v0.1.13^65^, as well as Q20 base quality score thresholds and heterozygosity adjustments following Nielsen and Paul^66^ and Zhang and Cao^67^. The resulting datasets were organized into four major analytical goals. (1) The kinship dataset includes all 147 individuals, with missing data retained, resulting in 100,754,525 SNPs. (2) The population structure dataset includes 66 unrelated individuals (after removing close kinship within three generations, as detailed later in the kinship inference section) and all 147 individuals. To obtain a set of putatively neutral and independent SNPs, we allowed ≤20% missing data, excluded singletons, removed variants within coding sequences and their 200-kb flanking regions, and thinned SNPs by retaining only one site per 200-kb window. This yielded 13,129 (unrelated set) and 13,125 SNPs (full set). (3) The genetic diversity dataset includes 66 unrelated individuals and all 147 individuals, filtered with ≤20% missing data, yielding 92,413,365 and 97,438,546 SNPs, respectively. For both datasets, an additional version retaining both invariant and variant sites was generated specifically for unbiased estimation of nucleotide diversity and divergence using pixy^68^. (4) The genetic load dataset includes 66 unrelated individuals plus two outgroups (*T. distichum* and *C. japonica*), and 147 individuals plus the same outgroups, both filtered with ≤20% missing data, resulting in 234,330,345 and 240,014,660 SNPs, respectively. Additional specialized SNP datasets derived from these base filters are described in the relevant analysis sections below.

### Kinship inference and ancient-tree provenance classification

To assess genetic relatedness among ancient, wild, and cultivated individuals, we performed pairwise kinship inference using KING v2.1^69^. This method estimates kinship coefficients by evaluating the balance between shared heterozygous and homozygous genotypes across genome-wide SNPs and does not require prior information on population structure. The kinship analysis was conducted on the full SNP dataset comprising 147 individuals and 100,754,525 SNPs, with missing genotypes retained to maximize informative sites. Kinship coefficients were computed using the ‘--kinship*’* function, and degrees of relatedness were classified according to the thresholds provided in the KING manual. To minimize the impact of close kinship on population-level analyses, we additionally identified a set of unrelated individuals using the ‘--unrelated*’* function.

To aid interpretation of ancient-tree provenance, we combined kinship structure with geographic context and genetic affinity to nearby wild populations. Ancient trees occurring as tight local clusters of individuals related within three degrees of kinship, especially when they also showed clear affinity to geographically nearby wild populations, were interpreted as likely historically introduced. By contrast, ancient trees showing little or no close kinship and lacking clear affinity to nearby extant wild populations were interpreted as likely natural relics, representing remnants of historically more extensive wild stands. On this basis, the 64 ancient trees were classified as being of likely historical introduction or likely natural-relic origin.

### Population structure and phylogenetic clustering

Population structure was first inferred using STRUCTURE v2.3.4^32^ based on 13,129 SNPs from 66 unrelated individuals. *K* values ranging from 1 to 8 were tested with 20 replicates per *K* under an admixture model with correlated allele frequencies. To mitigate the effects of unequal sample sizes among clusters, we set POPALPHAS = 1 and ALPHA = 0.25 following Meirmans^70^. The optimal *K* was determined using Δ*K*, Ln(*K*) and PI index implemented in KFinder v1.0^71^. PCA was performed on the same SNP dataset using the R package SNPRelate v1.34.1^72^. Pairwise *F*_ST_ and *d*_xy_ between the two inferred genetic lineages (Zhujiang, *n* = 29; Minjiang, *n* = 32) were calculated using pixy^68^ in 200-kb non-overlapping windows.

To incorporate related individuals while accounting for kinship, we applied PC-AiR to the full dataset of 147 individuals (13,125 SNPs) using GENESIS^33^. To validate the genetic clustering inferred from PC-AiR, we conducted phylogenetic analysis using SVDquartets^34^ based on genome-wide SNP data from 149 individuals (147 ingroup samples plus 2 outgroup species). A total of 6,794 SNPs were used, with additional filtering to retain ≤20% missing data overall but none in the two outgroup individuals, remove singletons, and thin sites at 200-kb intervals. Based on the combined PC-AiR and SVDquartets analyses, individuals were classified into three genetic clusters for downstream analyses: 48 assigned to the Zhujiang lineage, 92 to the Minjiang lineage and 7 to the admixed group.

### Ancient polymorphism comparison

To assess whether admixed individuals represent ancestral relic lineages retaining ancient polymorphisms, we quantified private allele counts across genetic groups using the whole-genome SNP set (97,438,546 SNPs; 147 individuals). Admixed samples were defined a priori based on STRUCTURE ancestry, dispersed positions in the PCA/PC-AiR plots, and basal placements in the phylogenetic tree. For the analysis, we focused on the putative historical range, including six admixed individuals from Guangxi, Yunnan and central Vietnam together with non-cultivated individuals from the Zhujiang and Minjiang groups, thereby minimizing potential confounding from recent human-mediated translocations. Private allele counts were obtained with the ‘-singletons*’* option in VCFtools^65^, treating both singletons and doubletons as rare alleles observed in the focal individual. Rare-allele loads were then calculated for each individual as proportions of the total number of callable SNPs, allowing standardized comparisons across samples. These individual-level estimates were used to compare private allele richness among the admixed, Zhujiang and Minjiang groups.

### Demographic history inference

Long-term history was inferred using the PSMC model^35^. Individuals with ≥18× mean coverage, ≥10× site coverage and ≤25% missing data were selected following Nadachowska-Brzyska and Burri^73^. Heterozygous sites were called using the mpileup command in SAMtools^63^ with the parameters ‘-C50 -q20 -Q20*’*, and sites with coverage <10× or >100× were excluded. Analyses were conducted with standard PSMC parameters (-N30 -t15 -r5 -p ‘4 + 25*2 + 4 + 6’). Given that most sampled *G. pensilis* individuals occur in estuarine–deltaic wetlands, we plotted the estimated population sizes against a background of sea-level changes^44,45^. Generation time was set to 50 years, and the mutation rate to 2.96 × 10⁻^8^ substitutions per site per generation, corresponding to the Cupressaceae estimate of 5.92216 × 10⁻^10^ substitutions per site per year^74^.

Recent effective population size (*N*_e_) was estimated using GONE^37^ based on LD information. Analyses were performed separately for the Zhujiang (*n* = 29) and Minjiang (*n* = 32) lineages. Both two datasets were processed under standardized filtering criteria with no missing genotypes, and variant data were converted to PED/MAP with PLINK v1.9^75^. Because no linkage map is currently available for *G. pensilis*, we used recombination rate estimates from the closely related conifer *C. japonica*, which has a genome size of 9,100 Mb (ref. ^76^) and a linkage map length of 1,405.2 cM (ref. ^77^). These values correspond to an average recombination rate of approximately 0.154 cM Mb^−1^ (equivalent to 6.5 Mb cM^−1^), which was used in the GONE analyses. The high-recombination cutoff was set to hc = 0.01 to minimize artefacts from sampling noise and potential recent migrants. For each dataset, 100 replicate runs were performed, each subsampling 50,000 SNPs per chromosome under default settings.

To estimate divergence times between the Zhujiang and Minjiang lineages, we used a coalescent simulation-based method implemented in fastsimcoal2 v2.8^36^. A two-dimensional site frequency spectrum was generated using easySFS.py (https://github.com/isaacovercast/easySFS) based on the joint SNP dataset from unrelated 61 individuals belonging to these two lineages. Five demographic scenarios were tested to represent alternative hypotheses of lineage divergence and gene flow (Supplementary Fig. 10): strict isolation without gene flow, continuous migration after divergence, gene flow confined to either early or late post-divergence phases, and secondary contact following an initial period of isolation. Each model was run for 100 independent replicates, with 100,000 coalescent simulations per likelihood estimation (-n100000) and 50 conditional maximization algorithm cycles (-L50). The best-fitting model was identified as the one yielding the highest composite likelihood. Parameter uncertainty (95% confidence intervals) was evaluated by parametric bootstrapping with 100 simulated SFS datasets that were refitted under the best-supported model. Estimates were converted from coalescent to absolute time using a per-generation mutation rate of 2.96 × 10⁻^8^ and a generation time of 50 years^74^.

### Estimation of nucleotide diversity and individual heterozygosity

We estimated genome-wide diversity across the entire dataset, among conservation types, and among genetic lineages. Nucleotide diversity (*π*) was estimated in non-overlapping 200-kb windows using pixy^68^ based on SNP datasets that included both invariant and variant sites. Window-level values were averaged and weighted by the number of callable sites per window to obtain genome-wide estimates, using a fixed callable-sites file to ensure identical site inclusion. To minimize the confounding effects of close kinship, an unrelated dataset (*n* = 66) was used as the primary panel for estimating nucleotide diversity across genetic lineages (admixed, *n* = 5; Minjiang, *n* = 32; Zhujiang, *n* = 29) and conservation types (ancient, *n* = 28; wild, *n* = 12; cultivated, *n* = 26). In parallel, the full dataset (*n* = 147; 64 ancient, 33 wild, 50 cultivated; 7 admixed, 92 Minjiang, 48 Zhujiang) was also analysed to represent practical conservation scenarios in which kinship redundancy is retained. Uncertainty of genome-wide *π* was estimated by bootstrap resampling of the 200-kb windows within each group (1,000 replicates), from which 95% confidence intervals were obtained.

Genome-wide individual heterozygosity, defined as the ratio of heterozygous sites to the total number of callable sites, was estimated from genotype likelihoods using realSFS implemented in ANGSD^78^. Analyses were performed with the ‘-dosaf 1*’* option without calling major and minor alleles, using the SAMtools genotype likelihood model (-gl 1). Bases with quality scores below 20 (-minQ 20) and reads with mapping quality below 30 (-minMapQ 30) were excluded.

### Inference of ROH and inbreeding coefficient

ROH were identified using PLINK v1.9^75^ based on the full SNP dataset (*n* = 147) without prior LD or MAF filtering, as pruning may reduce ROH detection sensitivity^79^. We used scanning windows of 50 SNPs (--homozyg-window-snp 50), allowing at most one heterozygous site per window (--homozyg-window-het 1). A minimum SNP density of one SNP per 50 kb was required (--homozyg-density 50), and two consecutive SNPs separated by more than 500 kb could not be included in the same ROH (--homozyg-gap 500). ROHs were called with a minimum length of 100 kb (--homozyg-kb 100) and at least 50 SNPs per segment (--homozyg-snp 50), allowing a maximum of one heterozygous call per ROH (--homozyg-het 1), with all other parameters set to default values.

Individual inbreeding coefficients (*F*_ROH_) were calculated as the proportion of the genome covered by ROHs, obtained by dividing the total length of ROHs by the total genome size^80^. To infer the temporal scale of inbreeding, ROH segments were grouped into length classes corresponding to the expected number of generations since the common ancestor. The number of generations (*g*) was estimated using the formula *g* = 100/2 × *L*_cM_, where *L*_cM_ is the ROH length in centimorgans, as described by Thompson^81^. Using the recombination rate given above (0.154 cM/Mb)^76,77^, we convert physical ROH length into genetic distance according to *L*_cM_ = *r* × *L*_Mb_. Based on these calculations, ROH segments were assigned to four length classes: >0.812 Mb (<400 generations), 0.406–0.812 Mb (400–800 generations), 0.203–0.406 Mb (800–1,600 generations) and 0.100–0.203 Mb (1,600–3,247 generations), with 0.100 Mb corresponding to approximately 3,247 generations.

In addition, individual inbreeding coefficients based on heterozygosity (*F*_IS_) were calculated for the same SNP set using the --het option in VCFtools^65^.

### Assessing selection efficacy and genetic load

To characterize selection on coding regions across 147 individuals and genetic lineages, we estimated the genome-wide ratio of heterozygosity at 0-fold and 4-fold degenerate sites. These sites were identified using the Python script get_degeneracy.py (https://github.com/zhangrengang/degeneracy/tree/master) based on the reference genome and its annotation, and nucleotide heterozygosity at 0-fold non-synonymous and 4-fold synonymous was calculated for each individual in non-overlapping 200-kb windows. We additionally quantified individual deleterious genetic load by annotating SNP effects on protein-coding genes using SnpEff v5.0^82^, classifying variants as synonymous (SYN), missense or loss-of-function (LOF). LOF mutations were defined as those involving gains or losses of stop codons, or losses of start codons. Missense SNPs were further classified as deleterious (score ≤0.05) or tolerated (score >0.05) using SIFT4G program^83^ with custom databases built from the UniRef90 protein dataset. To avoid reference bias in allele polarization and deleteriousness inference, we retained only SNPs without missing genotypes across all samples and for which both outgroups (*T. distichum* and *C. japonica*) exhibited consistent homozygous states. Ancestral and derived alleles were then inferred using Est-SFS^84^ with a posterior probability cutoff of 0.95 under a Rate-6 substitution model. We assumed ancestral alleles to be non-deleterious and derived alleles to be potentially deleterious. For each individual, we calculated the total number of derived alleles by counting heterozygous genotypes once and homozygous-derived genotypes twice. Finally, we estimated the relative proportions of homozygous and heterozygous derived alleles for deleterious missense (DEL) and LOF variants by normalizing them to the corresponding numbers of synonymous SNPs.

### Quantifying the irreplaceability of ancient-tree genetic variation

Given the rapid decline of ancient trees, we quantified how well their genetic variation is represented in cultivated and wild populations and assessed the conservation priority of each ancient individual. First, we extracted genotype calls from the full 147-individual VCF and converted them into ALT allele counts using BCFtools v1.15^85^. Genotypes were coded as 0 for ALT-absent (0/0 or 0|0), 1 for heterozygous ALT (0/1 or 0|1), and 2 for ALT-homozygous (1/1 or 1|1), while any genotype containing missing calls was treated as NA. We then summarized allele-level metrics for three broad populations: ancient trees (*n* = 64), cultivated trees (*n* = 50) and wild trees (*n* = 33), as well as for a set of complementary ancient subgroups. Ancient-status subgroups were defined using kinship relationships in combination with geographic information and comprised ancient natural relic individuals (*n* = 26) and historically cultivated ancient individuals (*n* = 38). In parallel, genetic-lineage subgroups were defined from population structure analyses and comprised admixed ancient individuals (*n* = 4), Minjiang ancient individuals (*n* = 43) and Zhujiang ancient individuals (*n* = 17). For each of these populations and subgroups, we calculated the ALT allele count (AC), the non-missing allele denominator (AN = 2 × number of non-missing genotypes), the allele frequency (*f*_a_ = AC/AN) and presence–absence indicators. Full-set allele frequencies across all 147 individuals were used as a common reference for rarity-based analyses.

Second, to measure how effectively cultivated and wild populations capture ancient-tree variation, we computed both unweighted allelic coverage (AC) and ωAC. Alleles were assigned to rarity bins based on full-set MAF (>5%, ≤5% and ≤1%; singletons excluded). For any ancient allele set AS and comparison population G (cultivated, wild or both), allelic coverage was calculated as AC = ∣AS ∩ AG∣/∣AS∣. To incorporate the disproportionate evolutionary importance of rare alleles, each allele was assigned a weight *ω*_a_ = −log_10_(*f*_a_), and weighted coverage was calculated as ωAC = ∑(*ω*_a_ × 1(a∈*G*))/∑*ω*_a_ where the indicator 1(a∈*G*) reflects whether the allele appears in cultivated, wild or combined populations.

Third, to assess the irreplaceability of individual ancient trees, we computed the IRR index, which measures the weighted load of rare ancient-specific alleles absent from other populations. Rare ancient alleles were defined as those occurring in at most two ancient individuals (ancient singletons or doubletons). For each ancient tree *i*, the set of rare alleles it carries is denoted *A*_*i*_. IRR was calculated as IRR_*i*_(*G*) = ∑ [*ω*_a _× (1 − 1(a∈*G*))], where the indicator 1(a∈*G*) reflects whether the allele appears in cultivated, wild, or combined populations. IRR therefore quantifies the uniquely retained rare variation of each ancient tree. Values were normalized to the 0–1 range by dividing by the total rarity weight of all rare alleles carried by that individual.

### Rarefaction analysis

To assess potential losses of genetic diversity under hypothetical scenarios of ancient-tree mortality, we performed rarefaction analyses using the panel of 66 unrelated individuals, which minimizes kinship redundancy and better reflects species-wide genomic variation than the full 147-individual dataset. We first evaluated the effect of losing all 28 ancient individuals. We then quantified diversity loss by removing ancient trees according to two grouping schemes: provenance (historically introduced, *n* = 11; putative natural relics, *n* = 17) and genetic lineage (admixed, *n* = 4; Minjiang, *n* = 14; Zhujiang, *n* = 10). Nucleotide diversity (*π*) was calculated in non-overlapping 200-kb windows using pixy^68^, following the same procedures described above.

### Reporting summary

Further information on research design is available in the Nature Portfolio Reporting Summary linked to this article.

## Supplementary information

Supplementary Information Supplementary Notes 1–5 and Figs. 1–21.

Reporting Summary

Peer Review File

Supplementary Tables Supplementary Tables 1–17.

## Source data

Source Data Fig. 1 Sampling locations, recorded ages, DBH measurements and vitality assessments.

Source Data Fig. 2 Kinship, population structure, phylogenetic relationships and private alleles.

Source Data Fig. 3 Long-term and recent demographic history.

Source Data Fig. 4 Genomic diversity, inbreeding and genetic load.

Source Data Fig. 5 Rare allele representation and genetic irreplaceability.

Source Data Extended Data Fig. 2 Chromosome-scale genome features.

## Data Availability

The raw sequence data reported in this study have been deposited in the National Genomics Data Center (NGDC; https://ngdc.cncb.ac.cn) under accession number PRJCA061991. The final genome assembly and genome annotation files are available via figshare at https://doi.org/10.6084/m9.figshare.32824982.v1 (ref. ^86^). Source data are provided with this paper.

## Code availability

Code used for the main analyses in this study is publicly available via GitHub at https://github.com/Hickory01/Ancient-Trees-Conservation-Genomics-of-Chinese-Swamp-Cypress.git.
