# Supplementary material for: Population history rather than tree age contributes to the evolutionary importance of ancient trees in an endangered conifer
Source: Nat Plants. 2026 Aug 11;12(8):1455–68. doi: 10.1038/s41477-026-02367-9 (PMC13481254; doi:10.1038/s41477-026-02367-9)
Supplement: Supplementary file 1 [file 41477_2026_2367_MOESM1_ESM.pdf]

nature plants

Supplementary information https://doi.org/10.1038/s41477-026-02367-9

Population history rather than tree age contributes to the evolutionary importance of ancient trees in an endangered conifer

In the format provided by the authors and unedited

Supplementary Note 1. Field survey, population status, and vitality assessment of *Glyptostrobus pensilis*

Field survey and population characteristics

Comprehensive field investigations of *G. pensilis* were conducted between 2023 and 2024 to document its current population status, habitat context, and individual vitality across its extant distribution range. Based on field observations, ecological setting, and management history, all recorded individuals were classified into three conservation types: ancient, wild and cultivated trees (Fig. 1a and Supplementary Table 1–3). In total, 147 individuals were surveyed, comprising 33 wild trees distributed across 6 geographic localities, 50 recently cultivated individuals from 9 sites, and 64 ancient trees spanning 44 locations.

Ancient trees, defined in this study as individuals older than 100 years persisting in human-dominated landscapes (recorded ages are summarized in Supplementary Table 3), are frequently preserved as fengshui trees in front of ancestral halls, village plazas, or temple ponds. Most are currently under formal government protection, often accompanied by protection signage or designated conservation sites (Supplementary Fig. 21). Notable concentrations of ancient individuals were recorded in parts of Jiangxi Province, including Yiyang County and Guixi City, where dozens of such trees remain. Despite their cultural significance, the overall vitality of ancient trees was generally low. During the present survey, more than ten ancient individuals that had been recorded as living within the past decade were found to have died. Major threats observed included encroachment by climbing vines (for example, *Ficus pumila*), burial or damage of roots and pneumatophores associated with road construction, and land-use change linked to local development.

Wild individuals occur exclusively in remnant submontane swamp habitats, typically at elevations of approximately 459–1,254 m, where hydrological and ecological conditions remain closest to the presumed natural state of the species. These individuals generally exhibit conical to umbrella-shaped crowns and comparatively robust growth, and were found either as isolated trees or in small groves under low anthropogenic disturbance.

Cultivated individuals are primarily distributed in botanical gardens, parks, campuses, and forest farms, and generally represented recent plantings (ca. 20–50 years old) in managed wetland environments. Although *G. pensilis* has historically been reported from multiple ex situ planting sites across China, including Beijing, Nanjing, and Wuhan, our field surveys confirmed that many such cultivated plantings have failed and no longer survive. Field observations suggest that these failures may be attributable to inappropriate management practices, such as prolonged inundation under the assumption of flood tolerance comparable to *Taxodium* species, or exposure to colder winter conditions at higher latitudes relative to the species' extant native range.

In addition to documenting population status, reproductive output was qualitatively assessed during seed collection. Wild individuals typically produced the highest quantities of seeds, followed by ancient trees, whereas recently cultivated individuals showed markedly reduced seed production. These differences likely reflect variation in reproductive maturity, habitat suitability, and management history.

Criteria for tree vitality classification

Tree vitality of surveyed *G. pensilis* individuals (n = 147) was assessed using a standardized multi-indicator framework integrating crown architecture, structural integrity, growth condition, reproductive status and habitat quality. Where present, evidence of natural regeneration (seedlings or saplings) in the immediate vicinity was also considered as a contextual indicator of population vitality. Anthropogenic disturbance was not included as a direct component of the vitality classification, but was recorded separately as a potential contributing factor influencing tree condition. Based on these criteria, individuals were assigned to one of five vitality categories (Supplementary Table 1), with representative reference images provided in Extended Data Fig. 1.

Healthy: Individuals exhibiting intact umbrella-shaped or pagoda-like crowns with dense foliage and no visible structural damage. Pneumatophores were well developed and actively extended from the basal trunk region. Trees showed vigorous annual growth and regular reproductive output. Individuals typically occurred in high-elevation (459–1,254 m) swamp habitats forming natural populations with relatively intact age structures; limited natural regeneration (seedlings and saplings) was occasionally observed.

Moderate: Trees generally at mature stages, often under cultivation, with overall intact structure and dense basal foliage. Trunks were slightly swollen at the base, and pneumatophores were commonly upright and visible. Growth and reproduction were continuous but moderately reduced compared with healthy individuals. Natural regeneration was rare or absent.

Fair: Individuals usually at early or late mature stages under cultivation, characterized by either partial apical crown dieback with otherwise intact structure, or by intact crowns exhibiting pronounced crown thinning. Localized structural damage, basal hollowing, or anthropogenic modification of pneumatophores was common. Growth vigor was substantially reduced, although some reproductive output could still occur. Trees were mostly found in cultivated environments with degraded habitat conditions. No natural regeneration was observed.

Poor: Trees at late mature stages exhibiting severe crown loss or extensive apical dieback, with large areas of trunk damage or hollowing. Growth was highly fragmented or largely arrested, reproductive output was minimal, and pneumatophores were typically absent or severely degraded. Habitats were generally of poor quality, some accompanied by heavy epiphytic plant loads. No natural regeneration was observed.

Dying: Individuals retaining only minimal living tissue (consistent with modular growth), often with sparsely distributed or largely collapsed crowns and widespread branch mortality. This category includes both senescent individuals and severely stressed trees. Pneumatophores were nearly imperceptible, and evidence of growth or reproduction was very weak. Trees typically occurred in severely degraded habitats, with a very high likelihood of imminent mortality. Natural regeneration was entirely absent.

In general, individuals classified as Healthy were more frequently associated with relatively intact high-elevation swamp or wetland habitats and occurred as small natural

populations. Individuals classified as Moderate and Fair were more commonly recently cultivated trees occurring in artificial or semi-artificial wetland environments. These individuals were often present as sparse plantings or small stands used for protection or landscaping purposes, and were more frequently exposed to relatively low levels of anthropogenic or environmental stress. In contrast, individuals classified as Poor or Dying were more often ancient trees in similar settings, typically occurring as isolated individuals and including both historically cultivated individuals and likely natural relics. In some cases, poor condition in these ancient individuals may be associated with environmental degradation or anthropogenic disturbance rather than age alone. This classification scheme was applied consistently across all surveyed individuals to facilitate comparative analyses among conservation types and to enable transparent and repeatable assessment of population condition.

Supplementary Note 2. Kinship structure among ancient, wild and cultivated trees

To facilitate interpretation of kinship relationships among the 147 sampled *G. pensilis* individuals, we classified kinship relationships inferred using KING[1] into six categories. This framework facilitates interpretation of the potential genetic origins of ancient trees and their relationships with extant wild and cultivated populations. All interpretations are based on kinship analyses of the full dataset (n = 147), with results shown in Supplementary Fig. 7 and partially illustrated in Fig. 2a.

Kinship among ancient trees: A total of 64 ancient trees were sampled from 44 locations across six provinces in southern China (Jiangxi, Fujian, Guangdong, Hunan, Guangxi and Yunnan), with most individuals concentrated in Jiangxi (n = 29) and Fujian (n = 17). First- to third-degree kinship relationships were detected in 50 of the 64 individuals, forming five groups (a1, a3, b, c and d). In northeastern Jiangxi, 27 of 29 individuals formed a single first-degree related cluster, most likely half-sibs, consistent with a strong founder effect associated with human-mediated introduction. In addition, 15 individuals from Fujian and six from Guangdong and Guangxi formed two kinship groups each (a3 and b; c and d), in all cases involving geographically proximate individuals.

Kinship among wild individuals: A total of 33 wild individuals were sampled from six localities (five in China, n = 30; one in Vietnam, n = 3). Of these, 30 individuals were involved in first- to third-degree kinship relationships, forming five groups (a2, a3, e, f and g). The pervasive relatedness within wild populations is consistent with elevated levels of inbreeding, likely reflects small effective population sizes and long-term fragmentation of remnant stands.

Kinship among cultivated individuals: A total of 50 cultivated individuals were sampled from nine sites, including botanical gardens, managed forest systems and university campuses. Among them, 40 individuals were involved in duplicated or first- to third-degree kinship relationships, forming five groups (a1, a3, d, h and i). One group (i), comprising three individuals, was identified as duplicated genotypes, consistent with clonal propagation (e.g. cuttings). Kinship links spanning multiple sites (groups d and h; involving 10 individuals) suggest potential exchange of planting material among cultivated populations.

Kinship between ancient and wild individuals: Substantial kinship connections were observed between ancient and wild individuals. Specifically, 27 ancient trees from Jiangxi and 11 from Fujian showed first- to third-degree relatedness to three nearby wild populations (group a1–a2–a3), consistent with likely historical introduction from local wild sources. The remaining 26 ancient trees showed no detectable affinity to extant wild populations and are therefore inferred to represent likely natural relics.

Kinship between ancient and cultivated individuals: Extensive kinship links were detected between ancient and cultivated individuals. In group a1, 27 ancient trees were related to 14 cultivated individuals distributed across five sites, whereas group d linked six ancient and six cultivated individuals across four sites. Given the old age of ancient trees, these patterns suggest that cultivated individuals were derived, directly or indirectly, from ancient germplasm. In addition, group a2 indicates that some cultivated

individuals in Fujian may have been introduced locally from nearby ancient trees or from wild individuals closely related to those ancient trees.

Kinship between wild and cultivated individuals: Kinship links between wild and cultivated individuals were comparatively limited, suggesting that cultivated populations were not commonly established directly from extant wild germplasm. The detected relationships (group a2–a3) were restricted to Fujian and likely reflect localized introduction events.

Overall, the observed kinship patterns are consistent with localized reproduction, limited dispersal, and human-mediated propagation, which together have likely contributed to shaping the present-day genetic composition of this critically endangered species, *G. pensilis*, across ancient, wild and cultivated populations.

Supplementary Note 3. Common garden seed germination experiment

To assess germination potential, mature cones were collected in mid-November 2023 from three representative sites corresponding to the three conservation types: Conghua (Guangzhou, China; ancient trees), Huaiji (Zhaoqing, China; wild populations), and the South China National Botanical Garden (Guangzhou, China; cultivated individuals; Supplementary Table 4). Cones with yellow–brown coloration indicating maturity were air-dried indoors and subsequently sun-dried for one week, then stored in dry containers at 4 °C until use.

Germination experiments were conducted in late March 2024 at the cultivation facilities of the South China National Botanical Garden (Supplementary Fig. 1). Prior to sowing, cones were processed by gentle mechanical striking and manual rubbing to remove cone scales and wing membranes. Seeds were surface-sterilized in 0.1% carbendazim solution for 2 h and thoroughly rinsed with sterile water. Seeds were sown on peat substrate in plastic trays and covered with approximately 2 cm of peat to ensure adequate soil contact and moisture retention. Trays were maintained under shaded conditions to provide suitable temperature and humidity.

Germination monitoring commenced 10 days after sowing and was conducted at 5-day intervals over a 30-day period. Germination potential was calculated as the proportion of viable seeds producing seedlings. Because *G. pensilis* seeds are extremely light (approximately 0.1 g per seed) and produced in large quantities, direct seed counting was impractical. Mean seed mass was therefore estimated based on measurements from 20 randomly selected cones and 100 randomly sampled seeds, and this estimate was used to derive germination rates.

Supplementary Note 4. Genome sequencing, assembly and annotation

Sample collection and sequencing

Fresh leaf tissues of *G. pensilis* were collected from an adult tree (accession SCBG00004966) at the Relic Plant Garden of the South China National Botanical Garden, Chinese Academy of Sciences (23.18746°N, 113.36625°E). Samples were snap-frozen in liquid nitrogen and processed by Novogene Co., Ltd. (Beijing, China) for genomic DNA extraction and library preparation. Three sequencing libraries were constructed: a short-insert Illumina NovaSeq 6000 platform (PE150) library (350 bp), yielding ~251.05 Gb of clean reads; a long-insert PacBio Sequel II library (10–20 kb), generating ~288.67 Gb of high-fidelity HiFi reads through Circular Consensus Sequencing; and a DpnII-based Hi-C library sequenced on the Illumina NovaSeq 6000 platform (PE150), producing ~421.77 Gb of high-quality data (Supplementary Table 5).

Genome size estimation and assembly

Illumina short reads were used to perform a 17-mer frequency analysis with GCE v1.0.2[2] to estimate genome size, heterozygosity, and repeat content. De novo genome assembly was carried out with hifiasm v0.16[3] using parameters-D10 -l0 -u based on PacBio HiFi reads. Hi-C data were mapped to the draft assembly using the BWA-MEM algorithm in BWA v0.7.17[4]. Misjoins and chimeric contigs were corrected with the ALLHiC_corrector module in ALLHiC[5], after which Hi-C reads were re-mapped and filtered using ALLHiC scripts (mapping quality > 30, uniquely mapped reads, edit distance < 5, mismatch count < 4, gap count < 2). A Hi-C contact heatmap was constructed using the ALLHiC_plot utility.

Assembly evaluation

Assembly completeness was evaluated using BUSCO v5.4.4[6] with two ortholog datasets: embryophyta_odb10 and gymnosperm_odb10[7] Accuracy was examined by mapping Illumina short reads back to the assembly with BWA v0.7.17[4] and SAMtools v1.19[8] to calculate mapping rate and genome coverage. Genome continuity was assessed by computing the Long Terminal Repeat Assembly Index (LAI) with LTR_retriever v2.9.0[9].

Repeat annotation

Repetitive elements were annotated using EDTA v2.0.0[10] with parameters '--sensitive 1 --anno 1 --evaluate 1'. EDTA integrates structure-based and homology-based TE prediction methods. The outputs were processed with RepeatMasker v4.1.4[11] to generate soft-masked and hard-masked versions of the genome.

Gene structure annotation

RNA was extracted from multiple *G. pensilis* tissues collected at different developmental stages (leaf, root, stem, female cone, male cone, and seed cone). RNA-seq libraries were sequenced on the Illumina NovaSeq 6000 platform (PE150). RNA-seq reads were aligned to the reference genome using HISAT2 v2.2.1[12] and sorted with SAMtools v1.19[8]. Gene prediction was performed using BRAKER2 v2.1.6[13], which employs GeneMark-ET[14] for HMM-based gene model training, followed by prediction with AUGUSTUS v3.5.0[15]. Protein sequences from 19 plant species (*Amborella*

trichopoda, *Arabidopsis thaliana*, *Azolla filiculoides*, *Cryptomeria japonica*, *Cycas panzhihuaensis*, *Ginkgo biloba*, *Gnetum montanum*, *Metasequoia glyptostroboides*, *Nymphaea colorata*, *Oryza sativa*, *Pinus tabuliformis*, *Populus trichocarpa*, *Salvinia cucullata*, *Selaginella moellendorffii*, *Sequoiadendron giganteum*, *Thuja plicata*, *Torreya grandis*, *Vitis vinifera*, *Welwitschia mirabilis*; Supplementary Table 8) were downloaded, clustered with CD-HIT v4.6.8[16] to remove redundancy, and used as homology evidence. Transcript assemblies generated with StringTie[17] were also provided to MAKER2[18]. Predicted gene models from MAKER2, along with gene models from SNAP[19] and trained models from AUGUSTUS and GeneMark-ET, were incorporated into a second round of MAKER2 annotation. Gene models with annotation edit distance (AED) scores > 0.5 were discarded. PASA v2.5.2[20] was used twice to update gene structures and produce the final annotation set.

Functional annotation

Predicted protein sequences were aligned to SWISS-PROT, NCBI NR, KOG, and eggNOG databases using BLASTP with an E-value cutoff of ≤1e−5. InterProScan v5[21] was employed to identify protein domains and assign InterPro entries, and Pfam domain annotation was performed using hmmscan[22]. Gene Ontology (GO) annotations were derived from eggNOG and InterPro results using a custom Perl script. KEGG pathway annotation was conducted with the KEGG Automatic Annotation Server (KAAS; http://www.genome.jp/tools/kaas/).

Supplementary Note 5. Synteny and gene family evolution analyses

Synteny analysis

Syntenic blocks within and between the genomes of *G. pensilis*, *M. glyptostroboides*[23], *Pinus tabuliformis*[24], and *Cycas panzhihuaensis*[25] were identified by conducting genome-wide BLASTP searches (-e 1e-10 -num_alignments 5), combined with MCScanX software[26]. For each pair of syntenic genes derived from whole-genome duplication (WGD) events, the synonymous substitution rate (K_s) was calculated using the Gamma-MYN algorithm[27] implemented in KaKs_Calculator 2.0[28].

Gene family comparison and expansion analysis

All protein sequences from 15 representative plant species—including 8 gymnosperms (*Cryptomeria japonica*, *Thuja plicata*, *M. glyptostroboides*, *Sequoiadendron giganteum*, *Torreya grandis*, *Pinus tabuliformis*, *Cycas panzhihuaensis*, and *Ginkgo biloba*), 4 angiosperms (*Arabidopsis thaliana*, *V. vinifera*, *Amborella trichopoda*, and *O. sativa*), 2 ferns (*Azolla filiculoides* and *Salvinia cucullata*), and 1 lycophyte (*Selaginella moellendorffii*)—were obtained from public genomic databases (Supplementary Table 8). To ensure data quality, only the longest isoform per gene was retained, and sequences containing premature stop codons, incompatible reading frames, or fewer than 50 amino acids were removed. Gene family clustering was performed using OrthoFinder v0.4.0[29], which identified 154 single-copy orthologous gene families shared among the 16 species. The first and second codon positions of these genes were concatenated to construct a phylogenetic tree using RAxML v8.2.8[30]. Divergence time estimation was performed using the approximate likelihood method implemented in the MCMCTREE program of the PAML package[31], with the following parameters: RootAge < 5.0, model = 4, alpha = 0.5, clock = 3, sample frequency = 2, burn-in = 20,000, nsample = 100,000, and finetune = "1: 0.1 0.1 0.1 0.01 0.5". Fossil calibrations[32] together with divergence time estimates from the TimeTree database (http://timetree.org/) were used to calibrate node ages (Supplementary Table 17). MCMC convergence was confirmed by examining effective sample sizes (ESS > 200) using Tracer v1.4 (http://beast.community/tracer), and then gene family expansion and contraction were inferred using CAFE v4.2.1[33]. To further evaluate the distribution of homologs, *G. pensilis* was compared against a broader panel of 22 land plant species (Supplementary Table 8) using OrthoFinder v0.4.0[29]. Genes lacking detectable orthologs were classified as putative species-specific genes. Gene Ontology (GO) enrichment analyses of expanded, contracted, and species-specific gene sets were performed using the R package clusterProfiler[34].

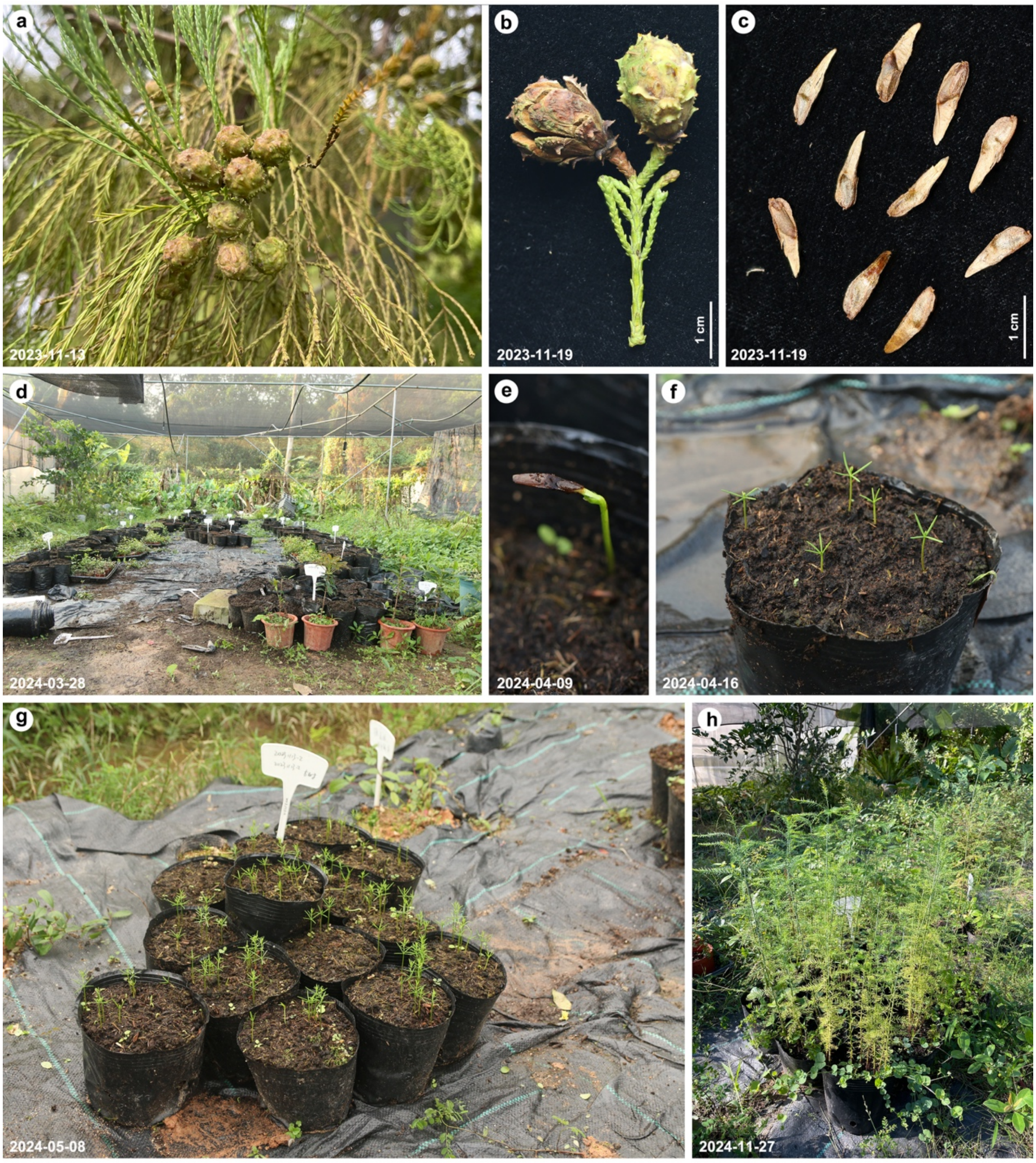

Supplementary Fig. 1 Year-long germination experiment and seedling monitoring of *G. pensilis.*

a Mature cones at the time of collection, showing a yellowish-brown coloration. **b–c** Cone dehiscence and mature seeds. **d** Germination experiment setup after seeds were stored at 4 °C for 4 months, conducted outdoors under a shaded canopy. **e** Germination status 10 days after sowing, with emerging seedlings breaking through the substrate. **f** Seedlings at day 17. **g** Seedlings at day 40. **h** Seedlings at 8 months (~1 m tall). The experiment was conducted in an outdoor greenhouse facility at the South China Botanical Garden, Chinese Academy of Sciences (Guangzhou, China).

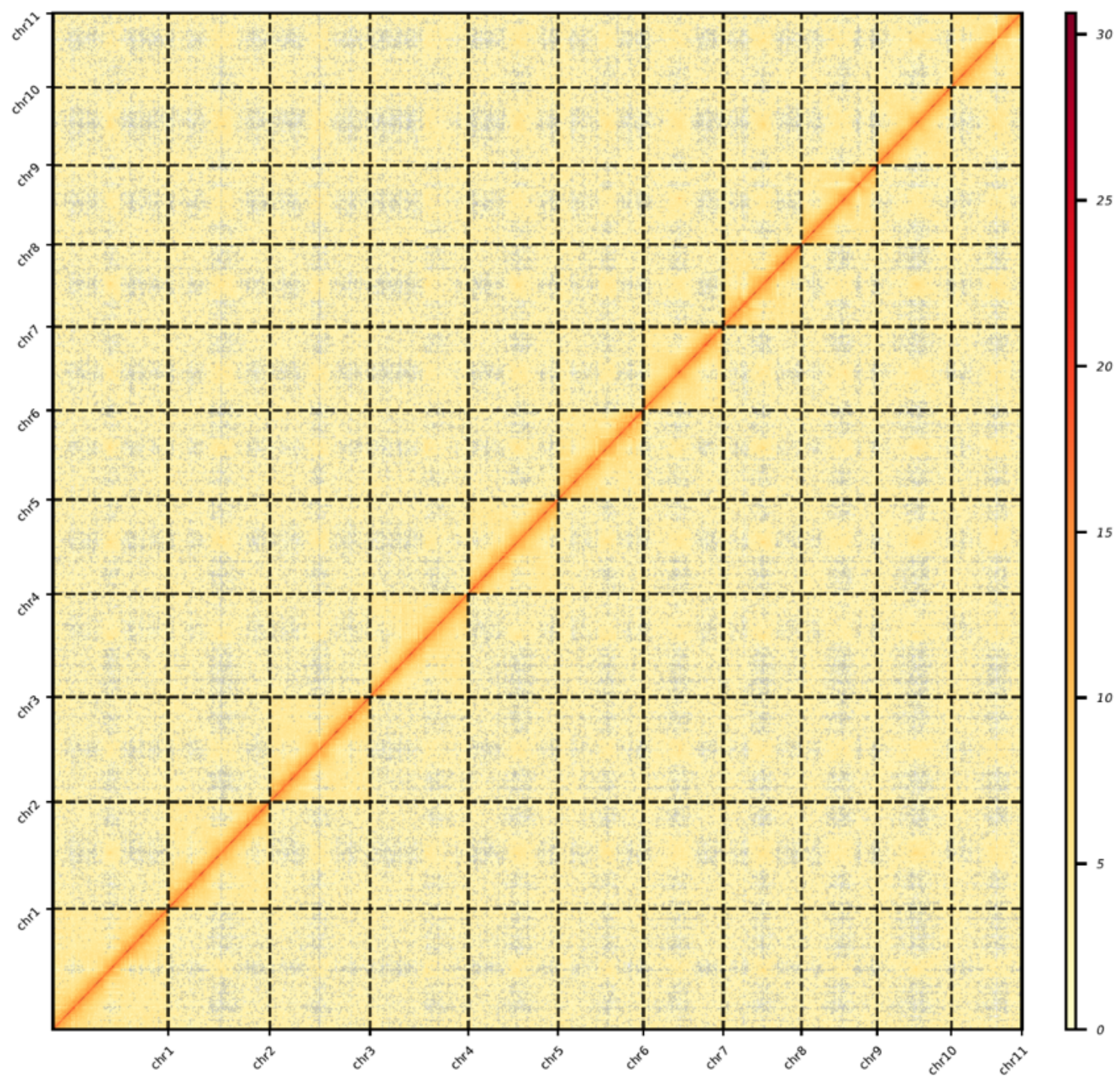

Supplementary Fig. 2 Hi-C contact map of the *G. pensilis* genome.

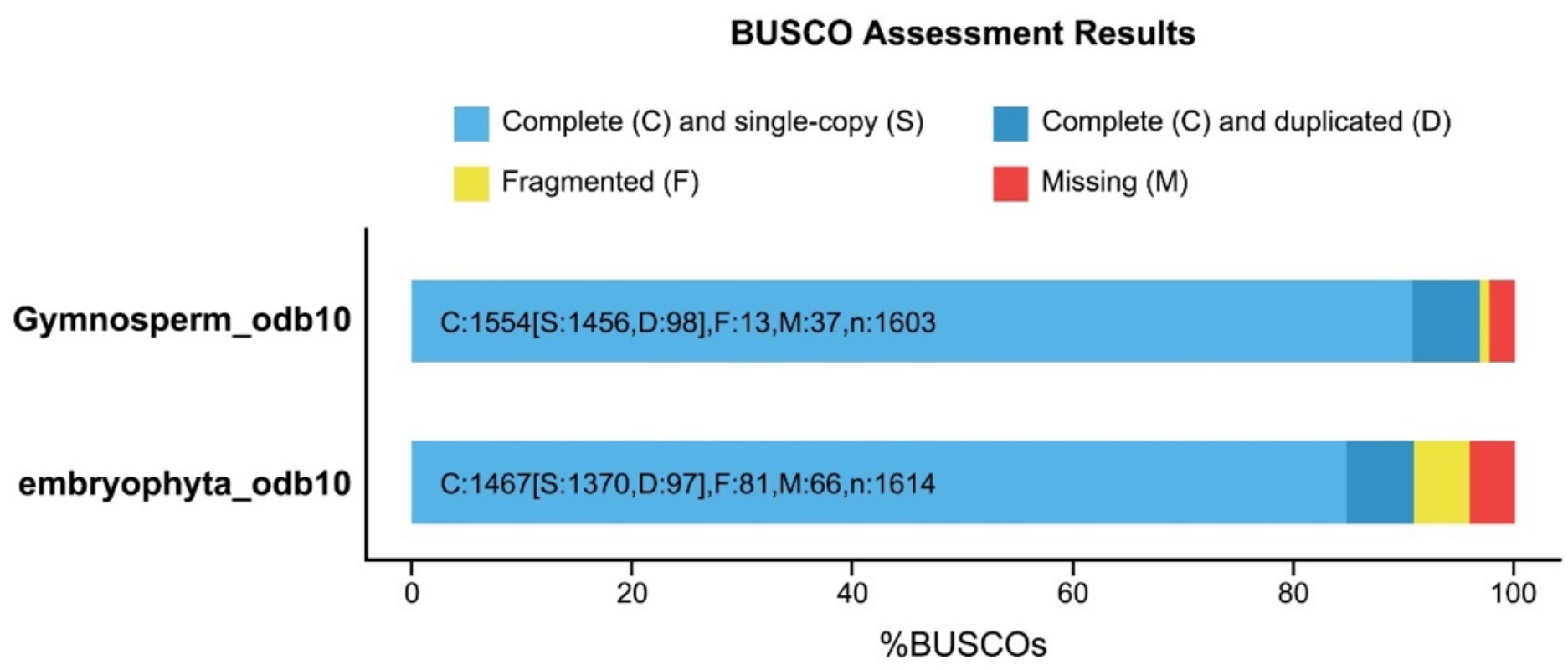

Supplementary Fig. 3 BUSCO assessment of the *G. pensilis* genome using the embryophyta_odb10 and gymnosperm_odb10 datasets.

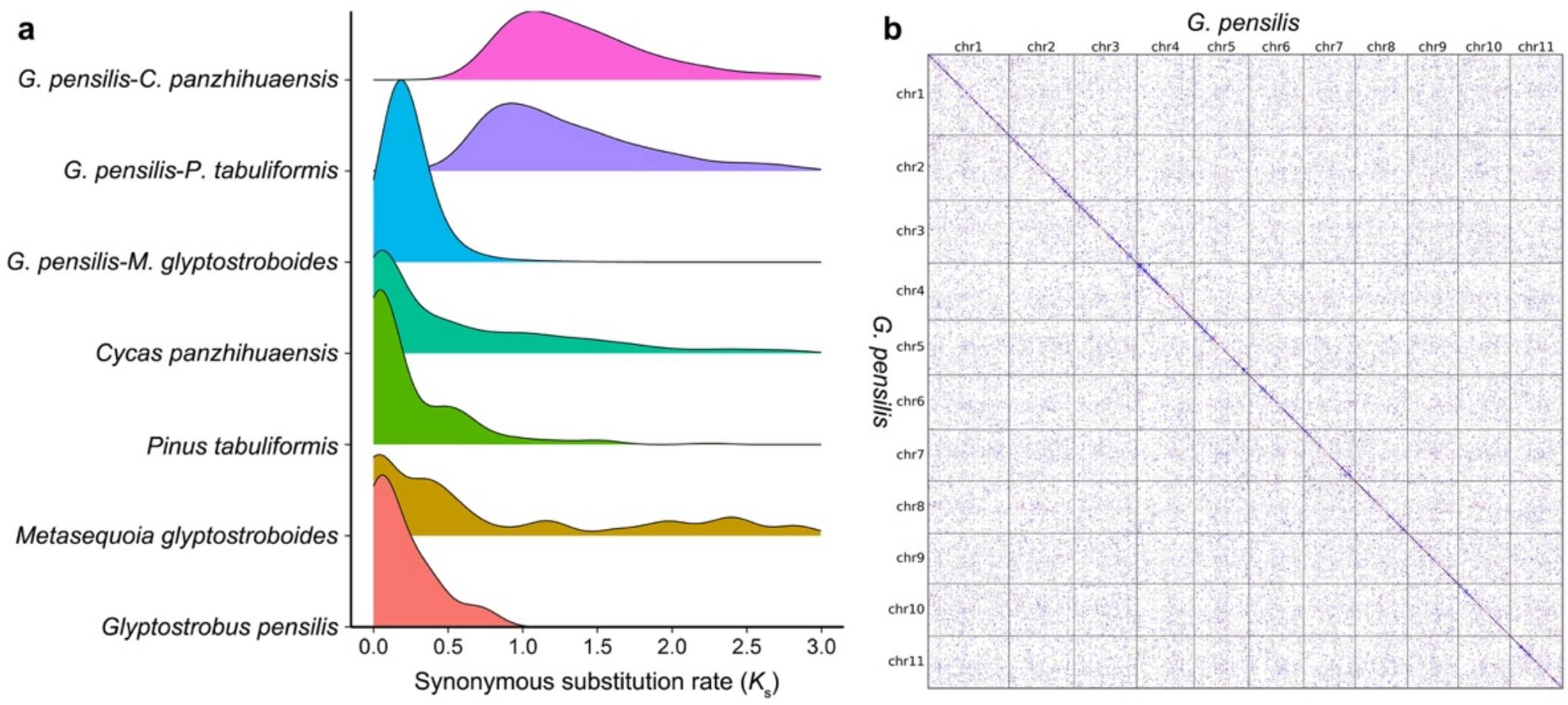

Supplementary Fig. 4 Intra- and interspecific genome synteny analyses of *G. pensilis* support the absence of a recent lineage-specific whole-genome duplication (WGD) event.

a Distribution of synonymous substitution rates (K_s) for syntenic gene pairs among the four gymnosperm species included in the analysis. **b** Intraspecific synteny dot plot of the *G. pensilis* genome.

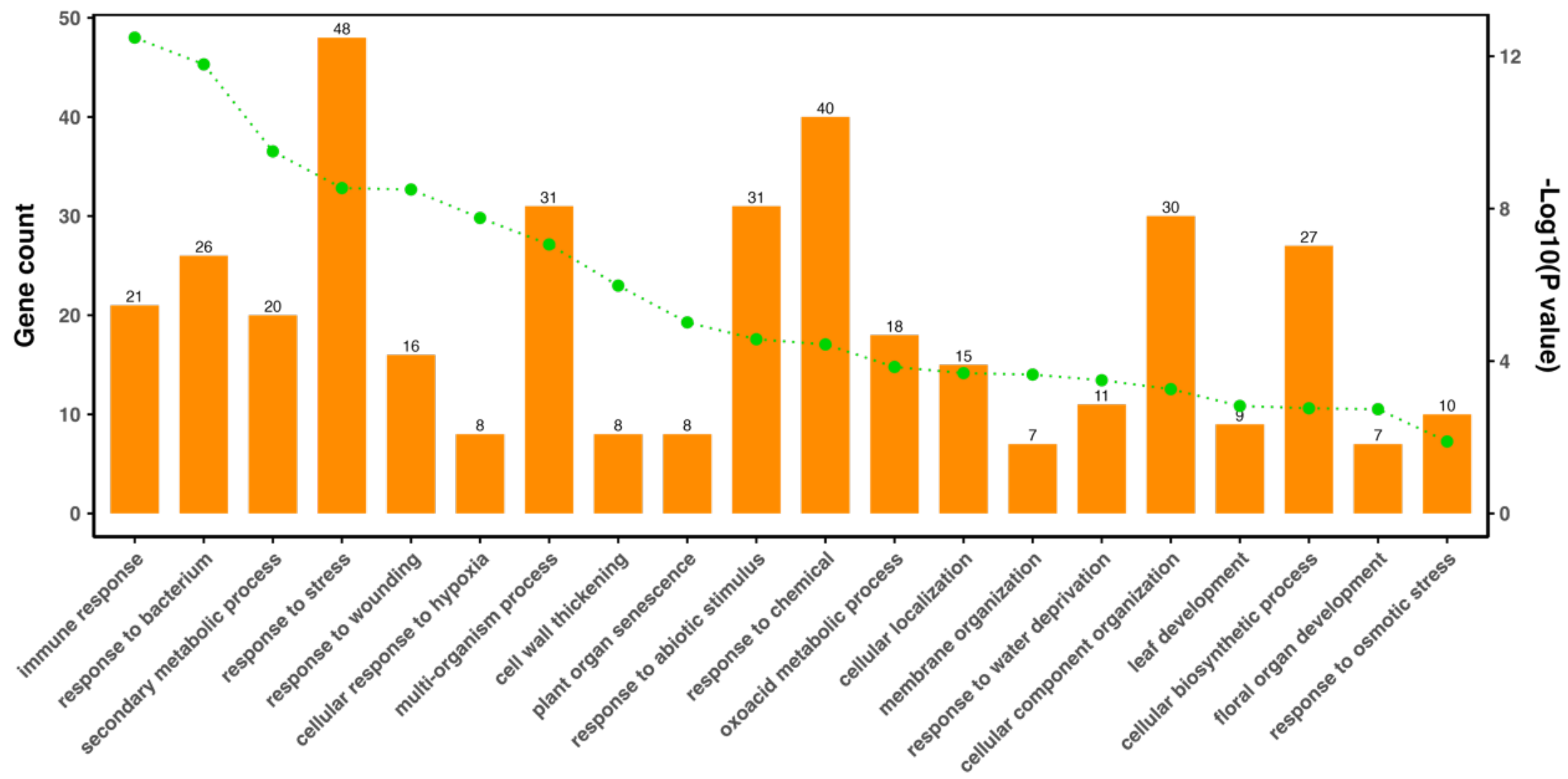

Supplementary Fig. 5 Gene Ontology (GO) enrichment of 1,989 putative *G. pensilis*–specific genes identified from comparisons with 22 land plant species. Enriched biological process (BP) terms are shown. Orange bars indicate the number of genes associated with each GO term, and green circles represent $-\log^{10}(P)$ values.

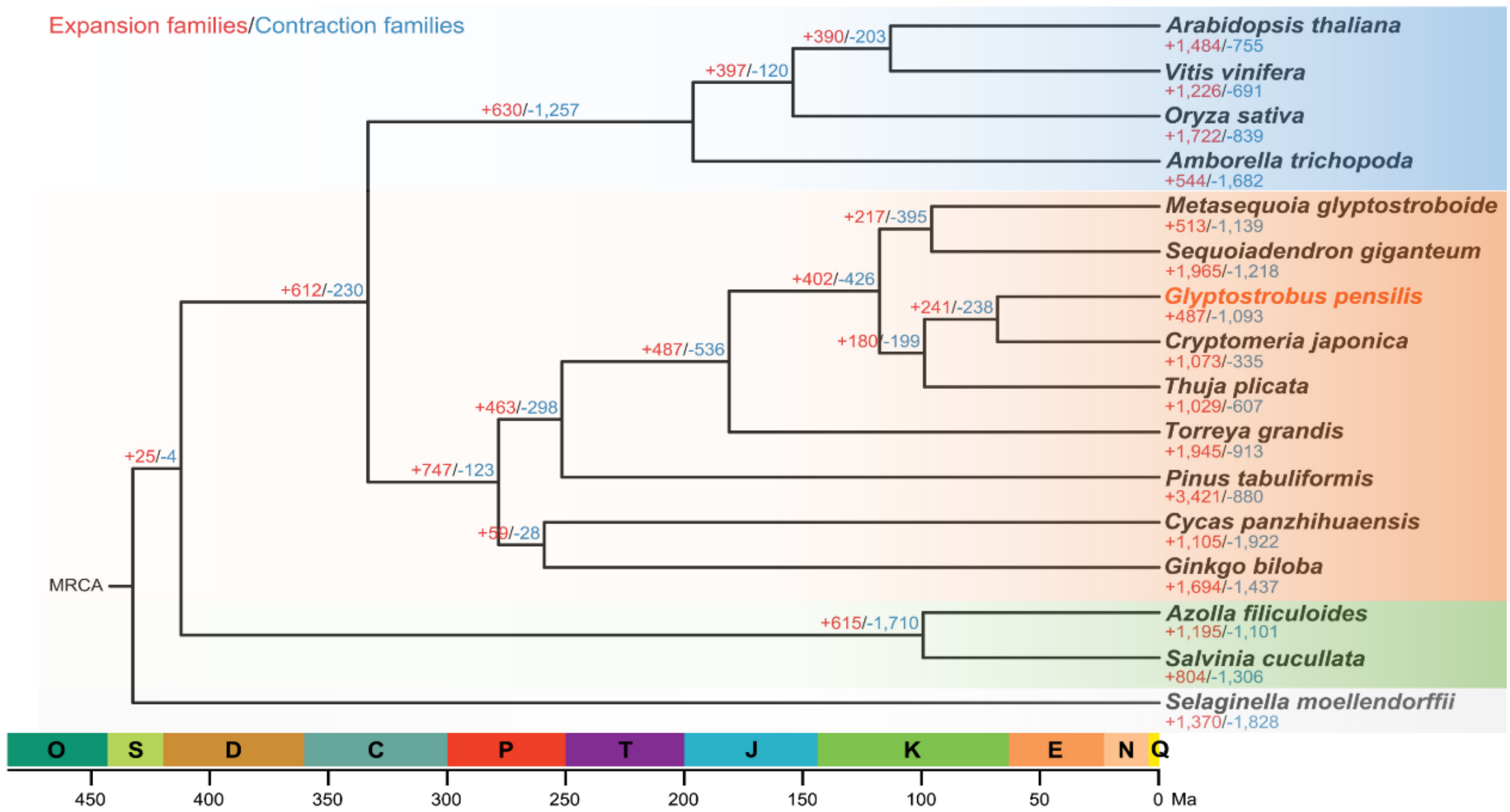

Supplementary Fig. 6 Phylogenetic relationships and gene family evolution of *G. pensilis* and 15 other representative species.

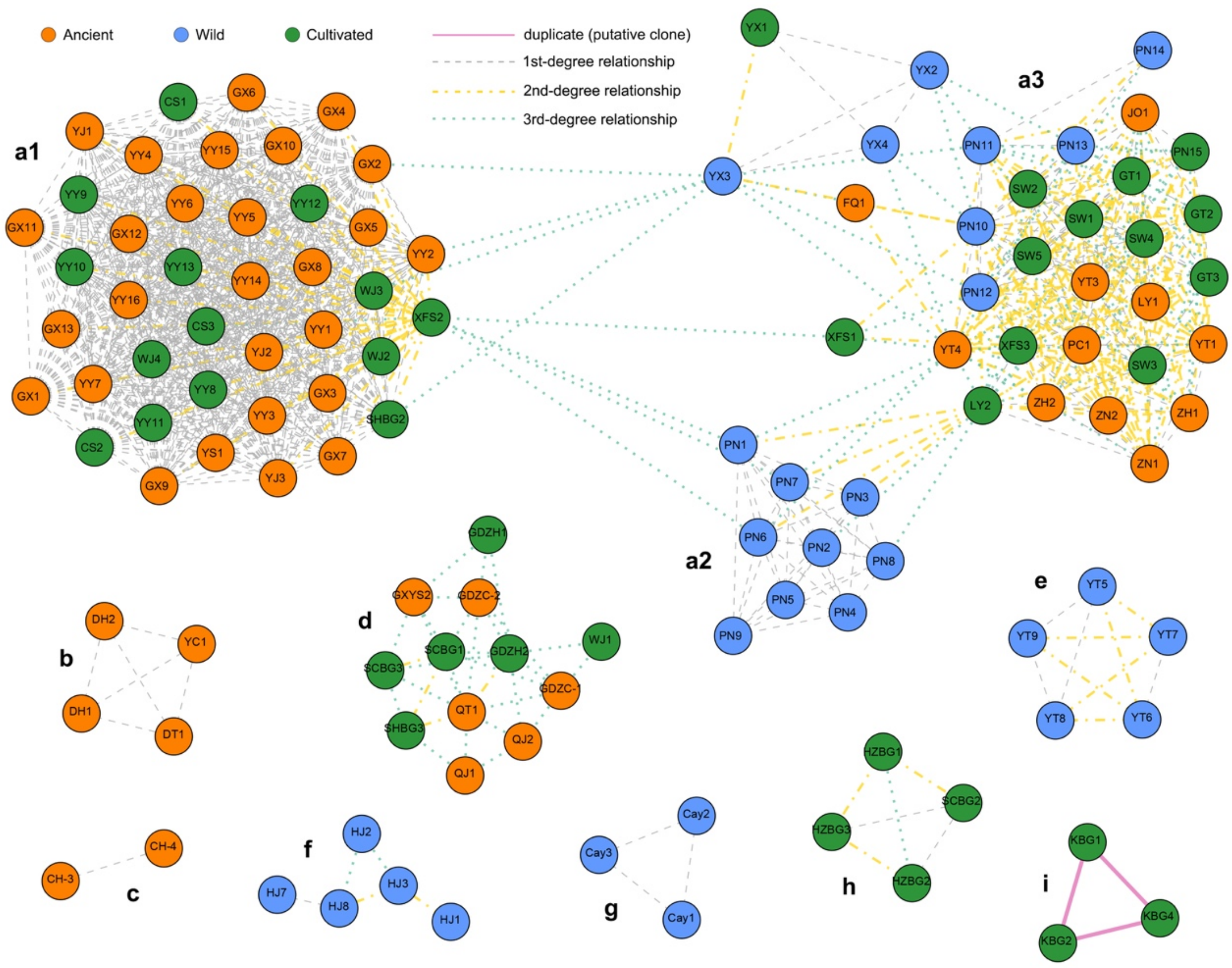

Supplementary Fig. 7 Pairwise kinship relationships among ancient, wild and cultivated individuals.

Ancient, wild, and cultivated trees are indicated by circles of different colors. Pairwise relatedness, ranging from duplicate or putative clonal replicates to third-degree relatives, is represented by four types of dashed lines. A total of nine kinship groups (a–i) were identified, with group a further subdivided into three subgroups (a1–a3). Individual names sharing the same prefix indicate samples originating from the same site (see Supplementary Table 1). Further details are provided in Supplementary Note 2.

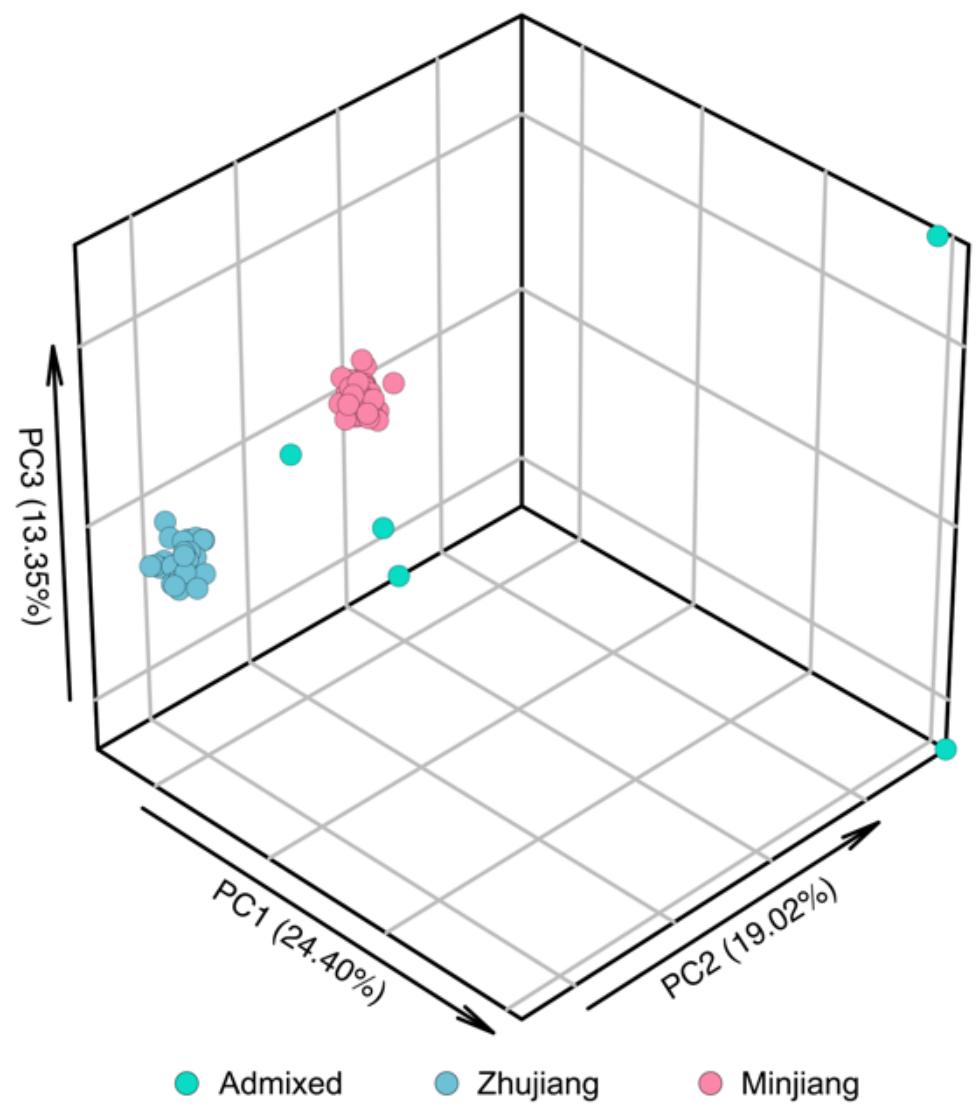

Supplementary Fig. 8 Principal component analysis (PCA) of 13,129 SNPs from 66 unrelated *G. pensilis* individuals.

The five dispersed points in the PCA correspond to the admixed individuals described in the main text. Among them, the two admixed individuals (FN1 and TD1) at the extreme end of PC1 are the same two samples that form the two outermost ingroup tips in the SVDquartets tree shown in Fig. 2d of the main text.

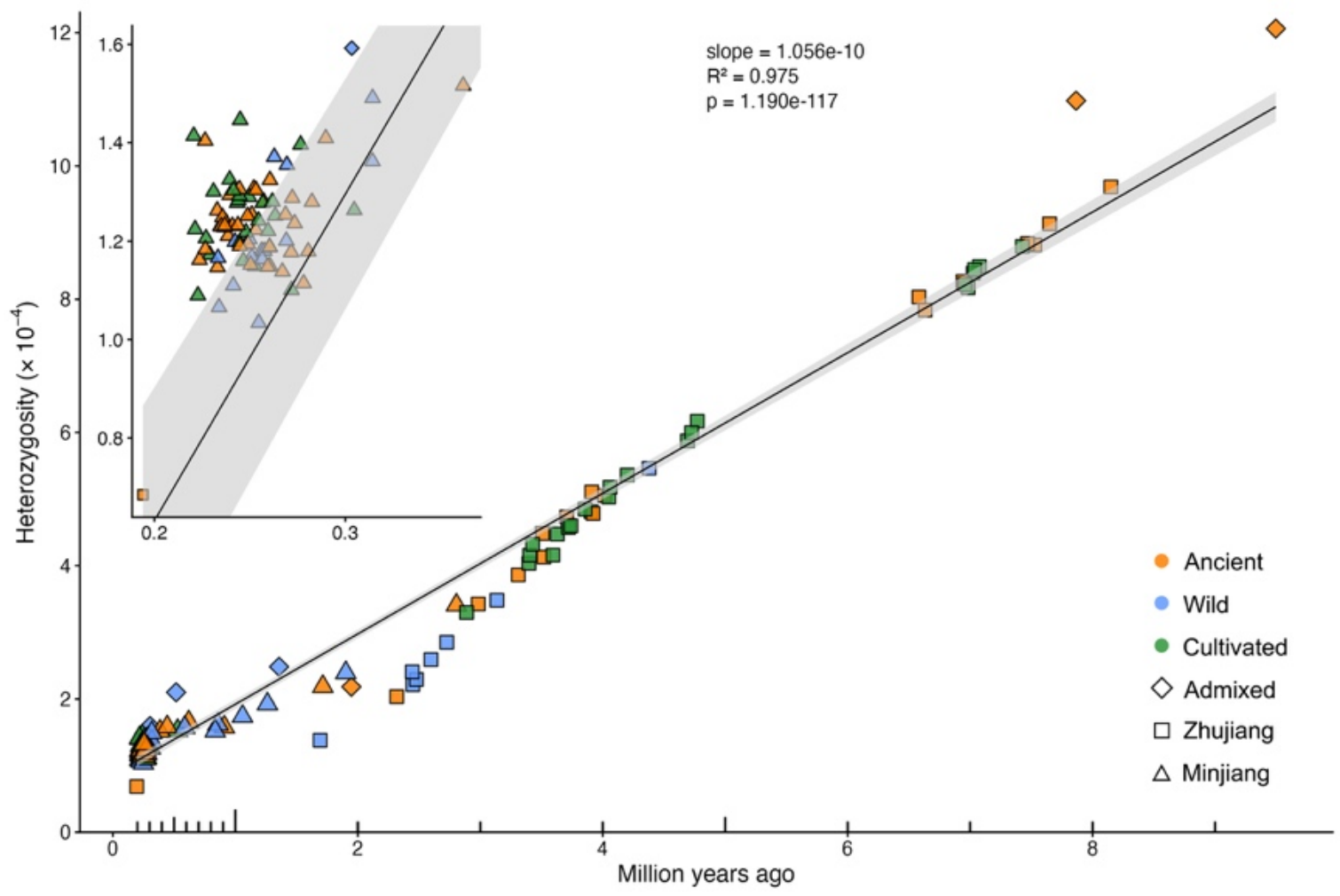

Supplementary Fig. 9 Relationship between genome-wide heterozygosity and recoverable PSMC depth.

Individual genome-wide heterozygosity (n = 147) plotted against the maximum temporal depth recovered by pairwise sequentially Markovian coalescent (PSMC) method. The inset shows an enlarged view of the region with time depths <0.4 million years (Ma).

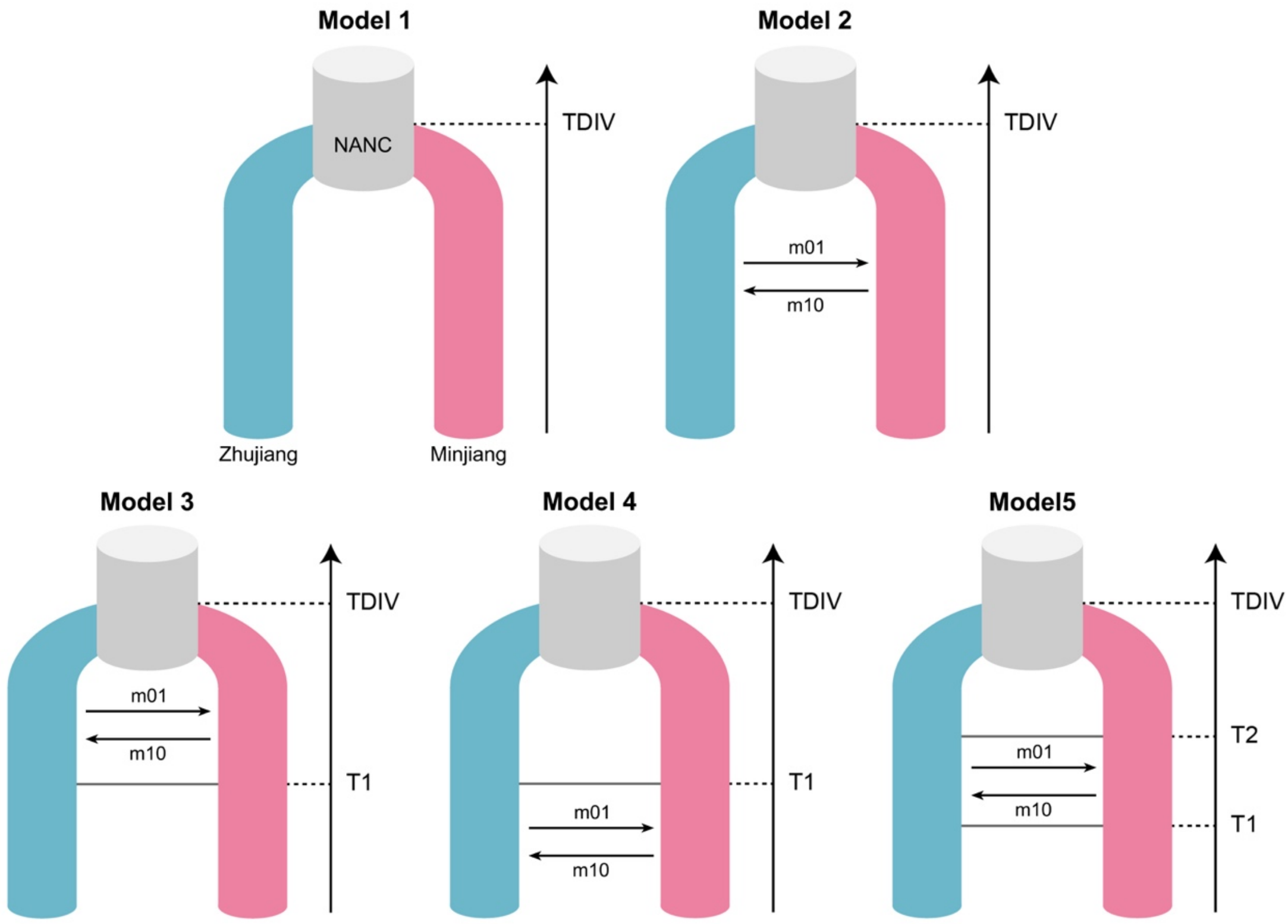

Supplementary Fig. 10 Five demographic scenarios representing alternative hypotheses of lineage divergence with or without gene flow in *G. pensilis*.

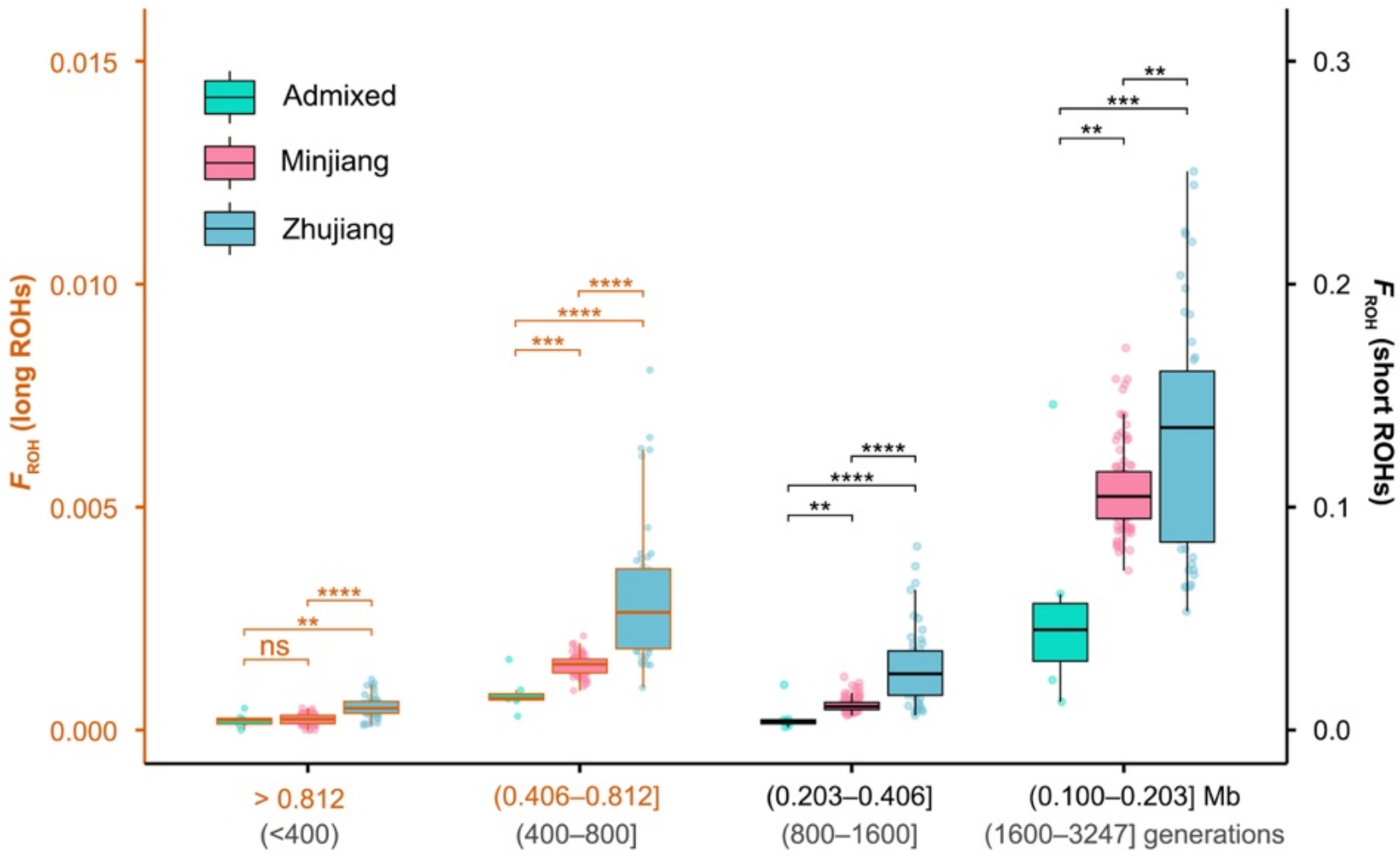

Supplementary Fig. 11 Distribution of ROHs in four length classes across the three genetic lineages based on all 147 individuals.

ROH length classes were used to approximate the time scale of inbreeding by converting physical ROH length (Mb) to genetic length (cM) using a recombination-rate proxy from the closely related conifer *Cryptomeria japonica* (≈0.154 cM/Mb; 1 cM ≈ 6.5 Mb), and then estimating generations since the common ancestor as $g = 100/(2L_{cM})$[35-37]. Box plots summarize the distribution across individuals: the center line indicates the median, the box bounds correspond to the 25th and 75th percentiles, and whiskers extend to 1.5× the interquartile range. Statistical significance for pairwise group comparisons was assessed using two-sided Wilcoxon rank-sum tests, with P values adjusted using the Benjamini–Hochberg (BH) procedure. Asterisks indicate adjusted significance levels (ns, not significant; $^*P < 0.05$; $^{**}P < 0.01$; $^{***}P < 0.001$; $^{****}P < 0.0001$).

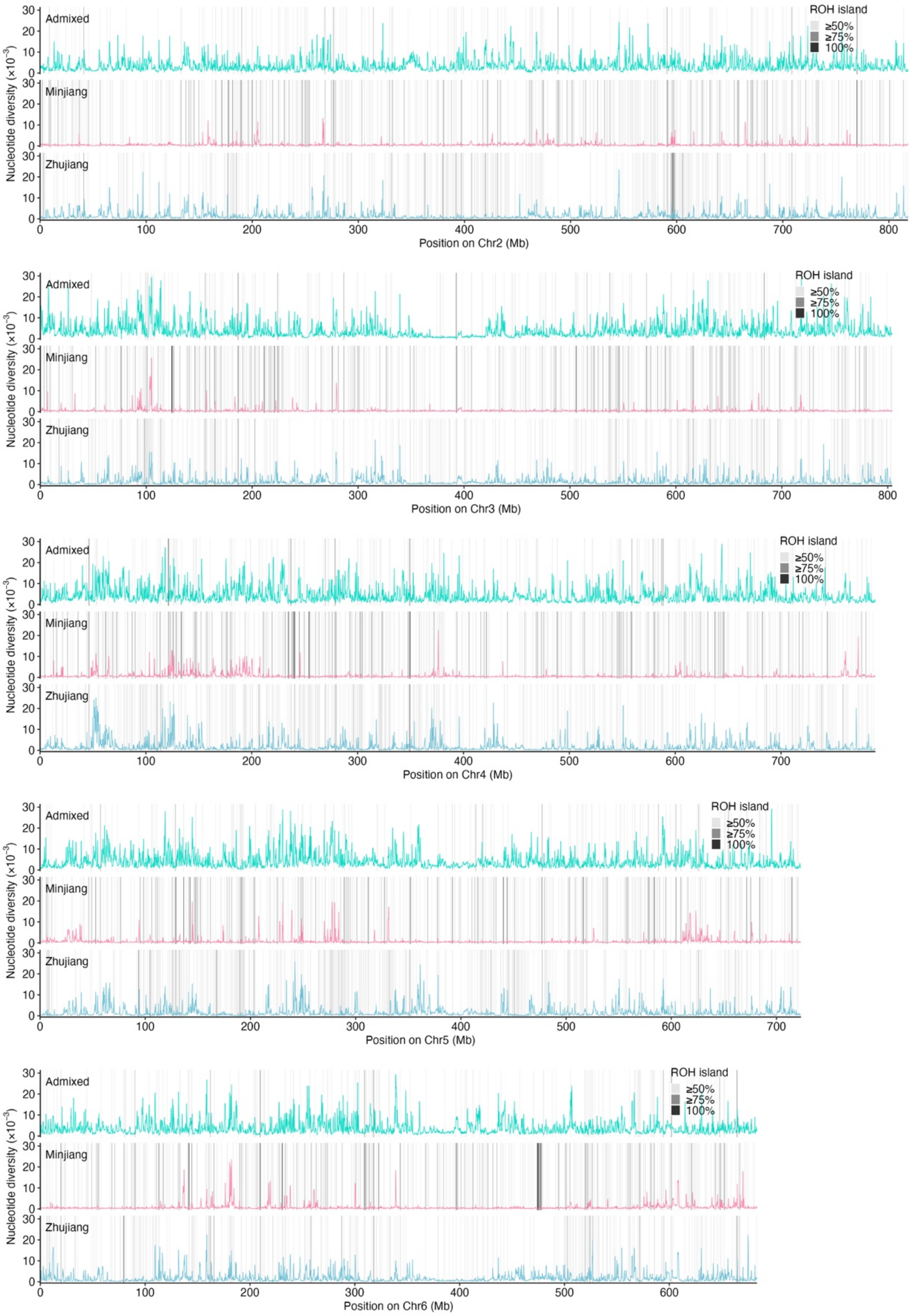

Continued on next page.

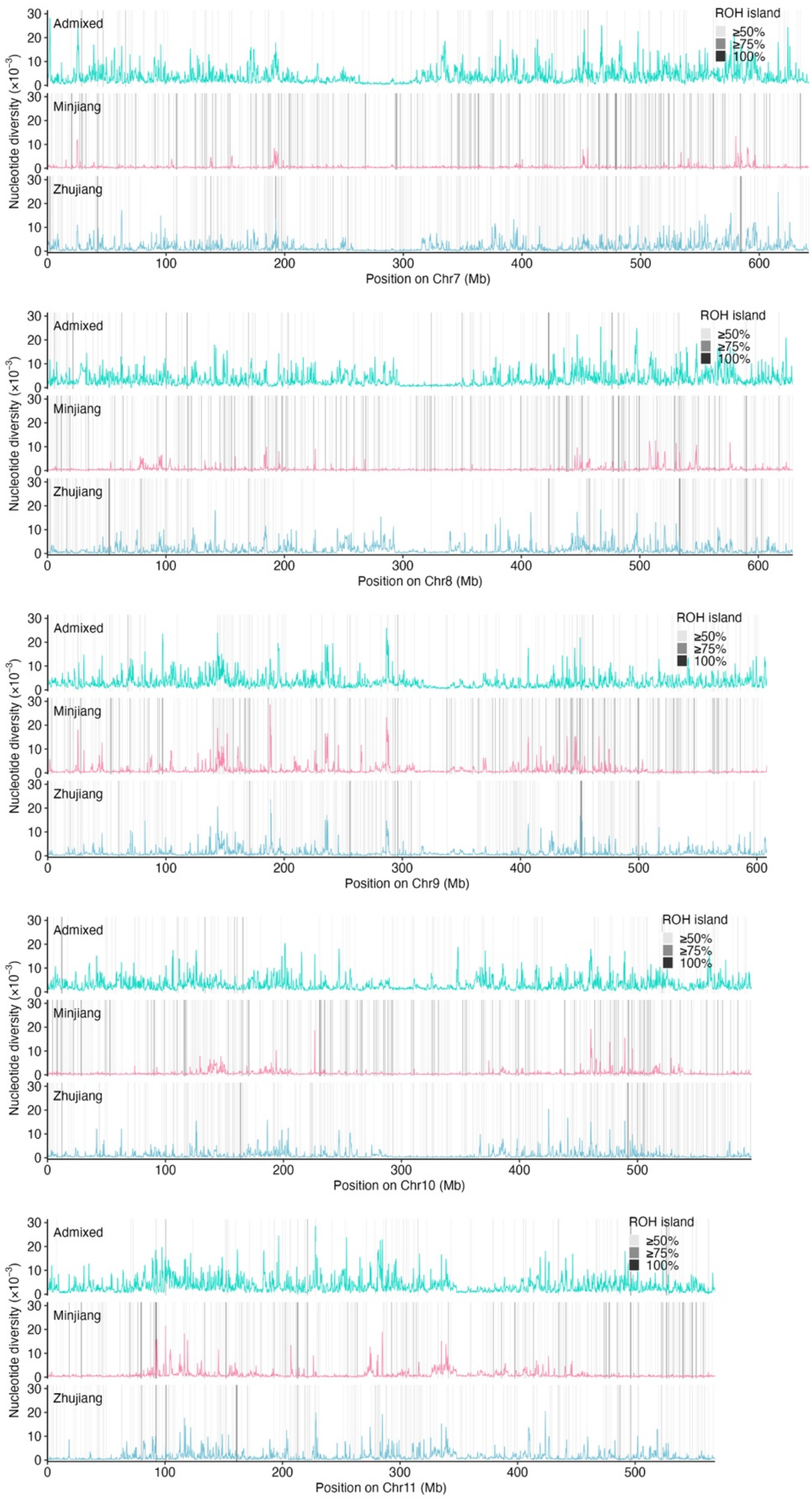

Supplementary Fig. 12 ROH islands and nucleotide diversity across the remaining 10 chromosomes.

ROH islands along the remaining 10 chromosomes (Chr2–Chr11) in the three genetic lineages, shown together with nucleotide diversity (π) in 200-kb sliding windows. Color intensity (light to dark) indicates regions shared by >50%, >75%, and 100% of individuals within each lineage.

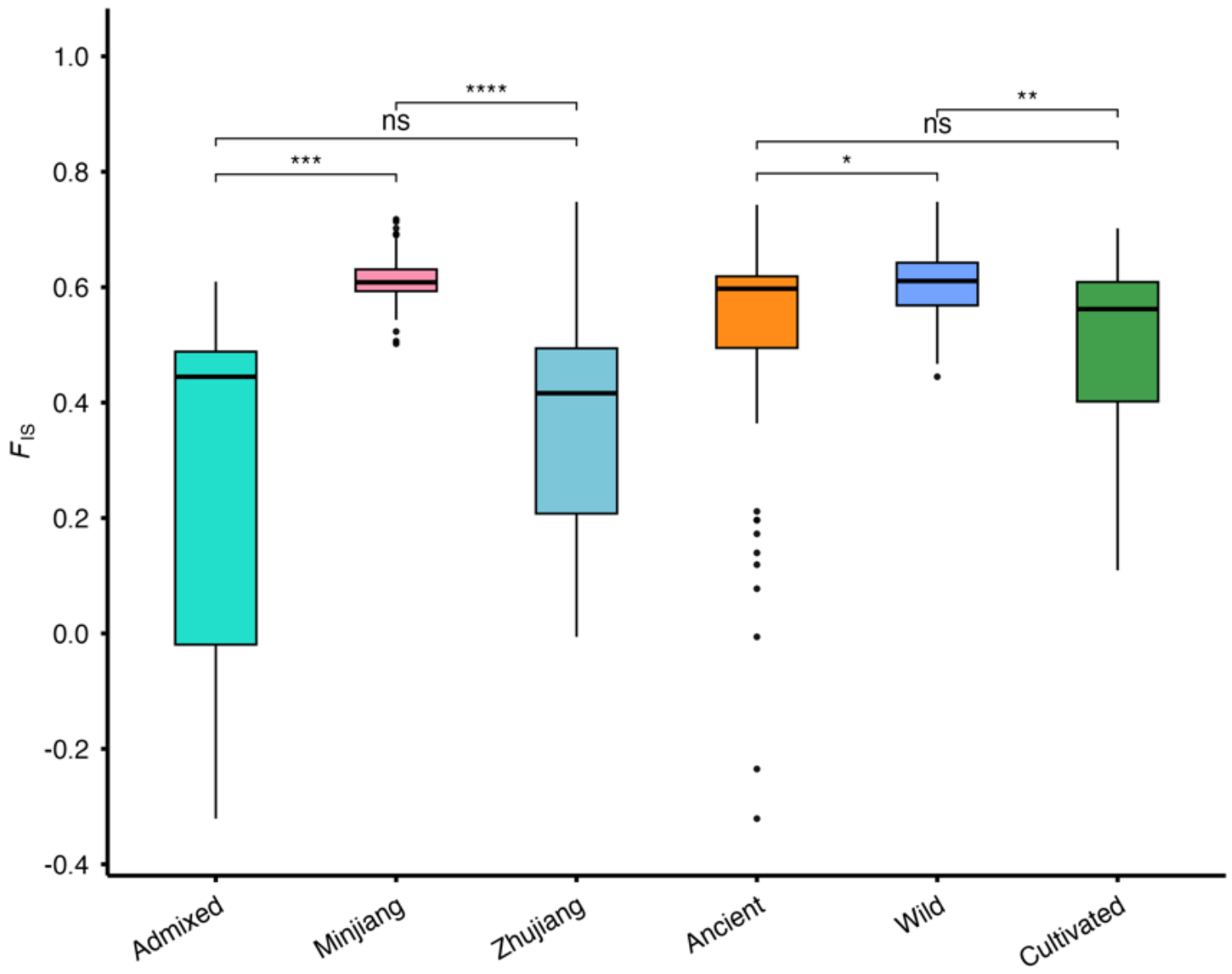

Supplementary Fig. 13 Comparison of F_{IS} among the three genetic lineages and three conservation types based on 147 individuals.

Box plots summarize the distribution across individuals: the center line indicates the median, the box bounds correspond to the 25th and 75th percentiles, and whiskers extend to 1.5× the interquartile range. Statistical significance for pairwise group comparisons was assessed using two-sided Wilcoxon rank-sum tests, with *P* values adjusted using the Benjamini–Hochberg (BH) procedure. Asterisks indicate adjusted significance levels (ns, not significant; $*P < 0.05$; $**P < 0.01$; $***P < 0.001$; $****P < 0.0001$).

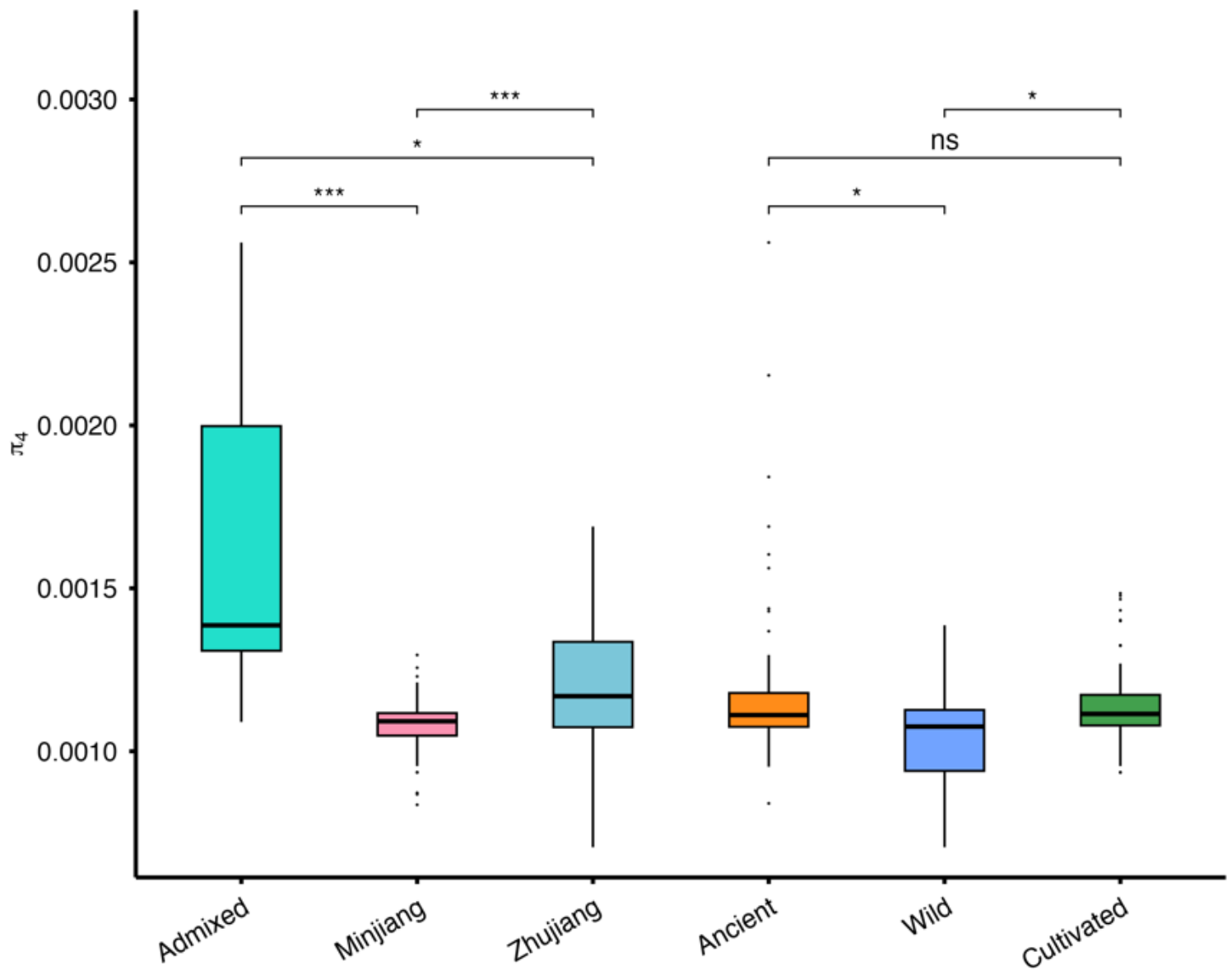

Supplementary Fig. 14 Heterozygosity at synonymous (4-fold degenerate) sites (π_4), compared among the three genetic lineages and three conservation types.

The analysis is based on the full 147-individual SNP dataset. Box plots summarize the distribution across individuals: the center line indicates the median, the box bounds correspond to the 25th and 75th percentiles, and whiskers extend to 1.5× the interquartile range. Statistical significance for pairwise group comparisons was assessed using two-sided Wilcoxon rank-sum tests, with *P* values adjusted using the Benjamini–Hochberg (BH) procedure. Asterisks indicate adjusted significance levels (ns, not significant; *$P < 0.05$; **$P < 0.01$; ***$P < 0.001$; ****$P < 0.0001$).

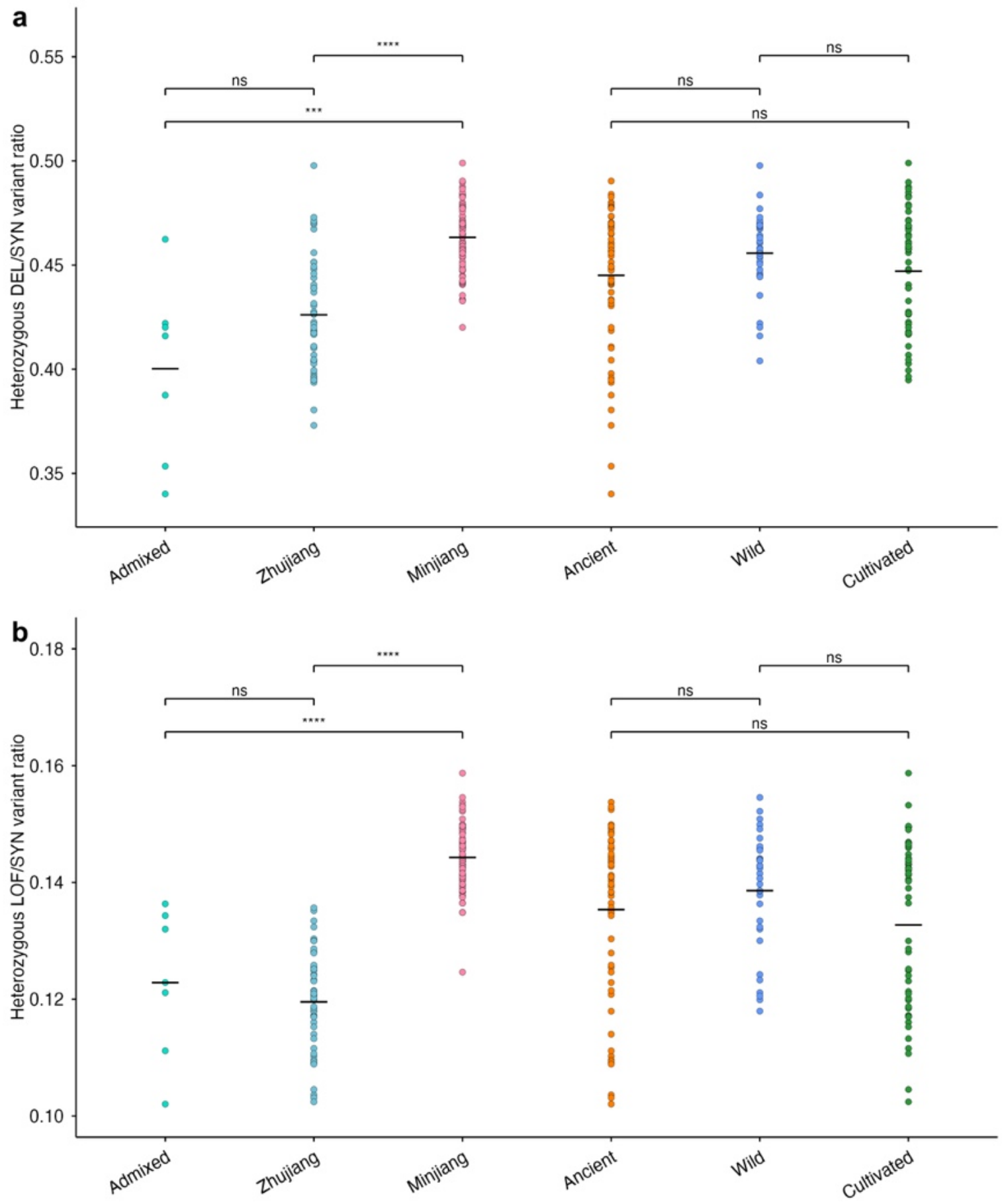

Supplementary Fig. 15 Ratios of heterozygous derived deleterious and loss-of-function variants to heterozygous derived synonymous variants.
a Ratios of deleterious (DEL) to synonymous (SYN) variants; **b** ratios of loss-of-function (LOF) to synonymous (SYN) variants. Horizontal bars denote average values. The analyses are based on the full 147-individual SNP dataset and are shown across the three genetic lineages and three conservation types. Statistical significance for pairwise group comparisons was assessed using two-sided Wilcoxon rank-sum tests, with P values adjusted using the Benjamini–Hochberg (BH) procedure. Asterisks indicate adjusted significance levels (ns, not significant; *$P < 0.05$; **$P < 0.01$; ***$P < 0.001$; ****$P < 0.0001$).

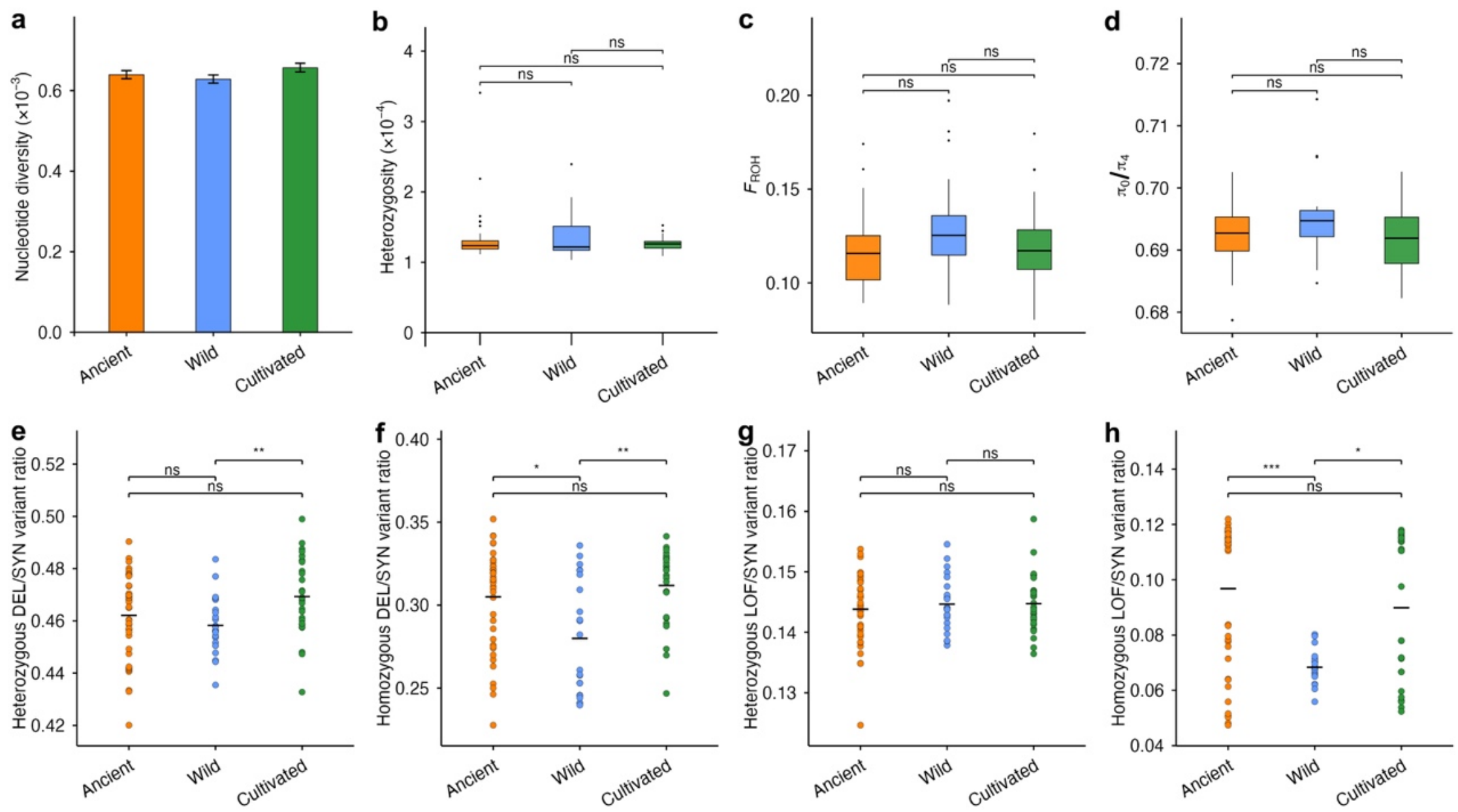

Supplementary Fig. 16 Genomic diversity, inbreeding, and genetic load across three conservation types within the Minjiang lineage.

a Genome-wide nucleotide diversity (π) across three conservation types (ancient, wild and cultivated). Error bars indicate bootstrap 95% confidence intervals estimated from 200-kb windows (1,000 replicates). **b** Mean genome-wide heterozygosity. **c** Comparison of F_{ROH}, defined as the proportion of the genome contained within runs of homozygosity (ROH). **d** Ratio of nucleotide diversity at 0-fold to 4-fold degenerate sites (π_0/π_4). Ratios of derived deleterious (DEL) to synonymous (SYN) variants in heterozygous (**e**) and homozygous (**f**) states. Ratios of derived loss-of-function (LOF) to synonymous (SYN) variants in heterozygous (**g**) and homozygous (**h**) states. Horizontal bars denote average values. All analyses are based on the full 147-individual SNP dataset and are shown for individuals within the Minjiang lineage (n = 92), including ancient (n = 43), wild (n = 22) and cultivated (n = 27) trees. Box plots summarize the distribution across individuals: the center line indicates the median, the box bounds correspond to the 25th and 75th percentiles, and whiskers extend to 1.5× the interquartile range. Statistical significance for pairwise group comparisons was assessed using two-sided Wilcoxon rank-sum tests, with P values adjusted using the Benjamini–Hochberg (BH) procedure. Asterisks indicate adjusted significance levels (ns, not significant; *$P < 0.05$; **$P < 0.01$; ***$P < 0.001$; ****$P < 0.0001$).

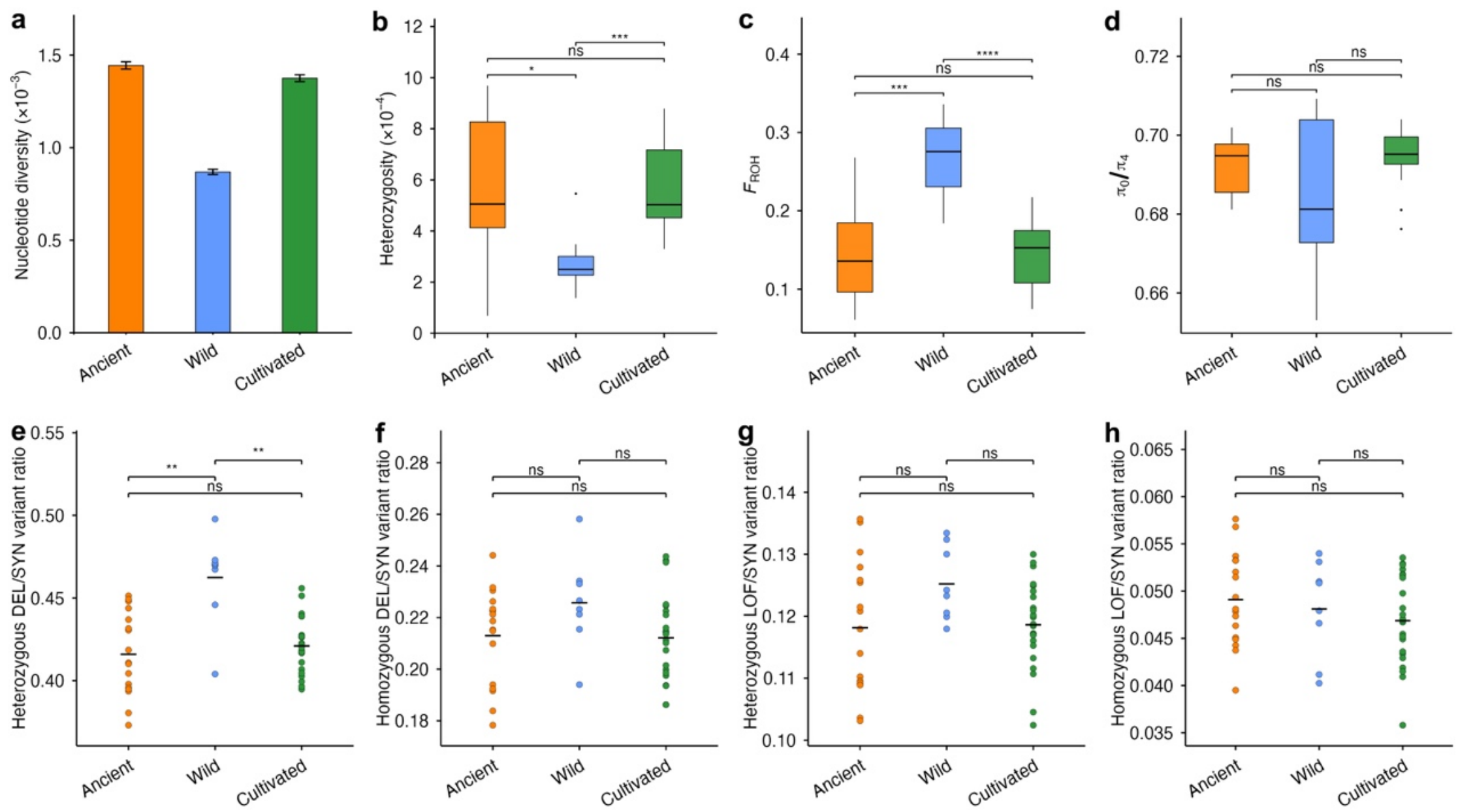

Supplementary Fig. 17 Genomic diversity, inbreeding, and genetic load across three conservation types within the Zhujiang lineage.

a Genome-wide nucleotide diversity (π) across three conservation types (ancient, wild and cultivated). Error bars indicate bootstrap 95% confidence intervals estimated from 200-kb windows (1,000 replicates). **b** Mean genome-wide heterozygosity. **c** Comparison of F_{ROH}, defined as the proportion of the genome contained within runs of homozygosity (ROH). **d** Ratio of nucleotide diversity at 0-fold to 4-fold degenerate sites (π_0/π_4). Ratios of derived deleterious (DEL) to synonymous (SYN) variants in heterozygous (**e**) and homozygous (**f**) states. Ratios of derived loss-of-function (LOF) to synonymous (SYN) variants in heterozygous (**g**) and homozygous (**h**) states. Horizontal bars denote average values. All analyses are based on the full 147-individual SNP dataset and are shown for individuals within the Zhujiang lineage (n = 48), including ancient (n = 17), wild (n = 8) and cultivated (n = 23) trees. Box plots summarize the distribution across individuals: the center line indicates the median, the box bounds correspond to the 25th and 75th percentiles, and whiskers extend to 1.5× the interquartile range. Statistical significance for pairwise group comparisons was assessed using two-sided Wilcoxon rank-sum tests, with P values adjusted using the Benjamini–Hochberg (BH) procedure. Asterisks indicate adjusted significance levels (ns, not significant; *$P < 0.05$; **$P < 0.01$; ***$P < 0.001$; ****$P < 0.0001$).

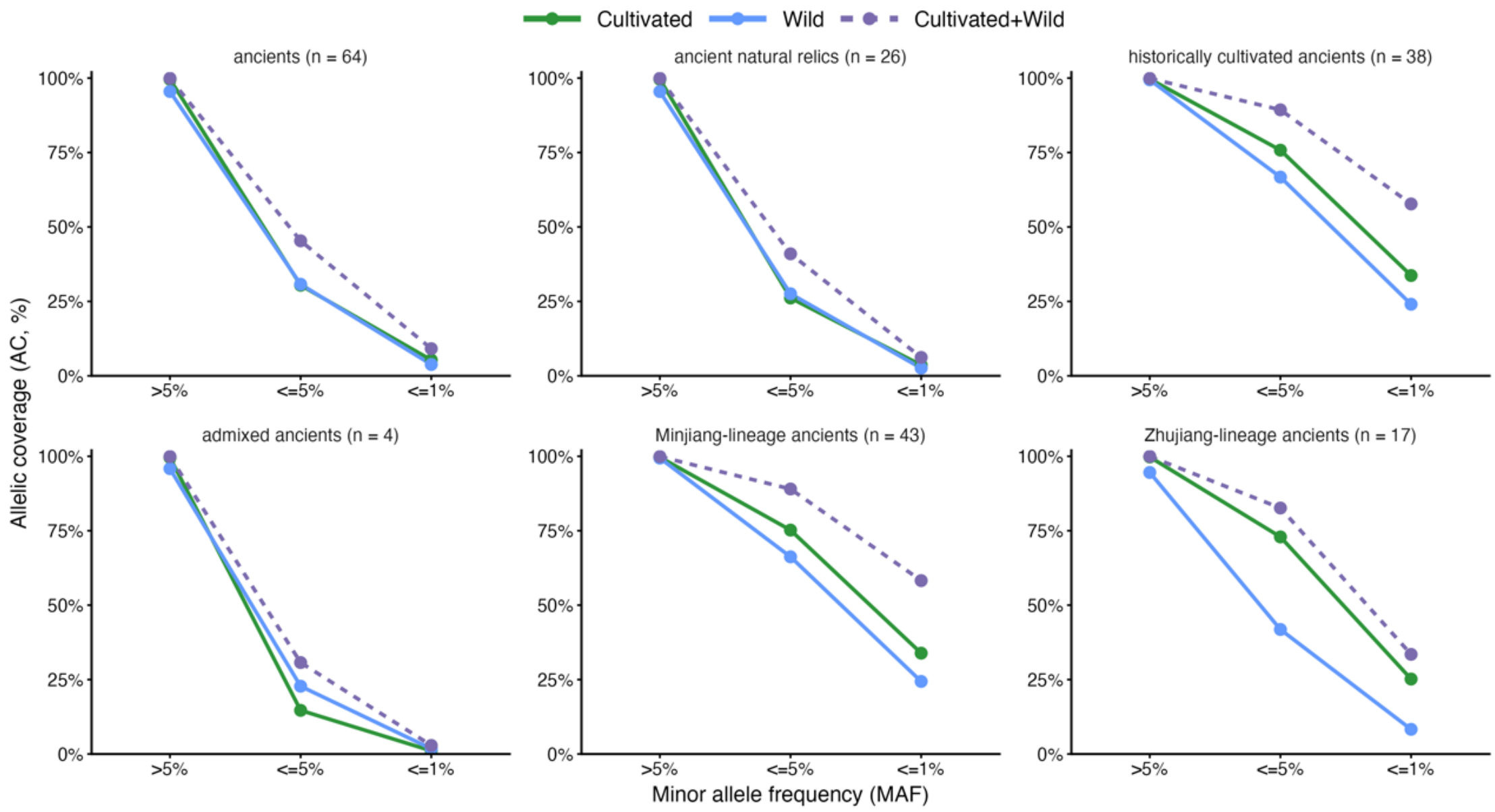

Supplementary Fig. 18 Rarity-unweighted allelic coverage (AC) of ancient-tree alleles.

Allelic coverage (AC; unweighted by rarity) across three minor allele frequency (MAF) thresholds, showing the proportion of variants captured by cultivated populations, wild populations, and the combined set (cultivated + wild), based on the full dataset (n = 147). The full ancient-tree set comprised 64 individuals. For stratified analyses, these ancients were grouped by origin (natural relics, n = 26; historically cultivated, n = 38), admixed group (n = 4), and genetic lineage (Minjiang, n = 43; Zhujiang, n = 17).

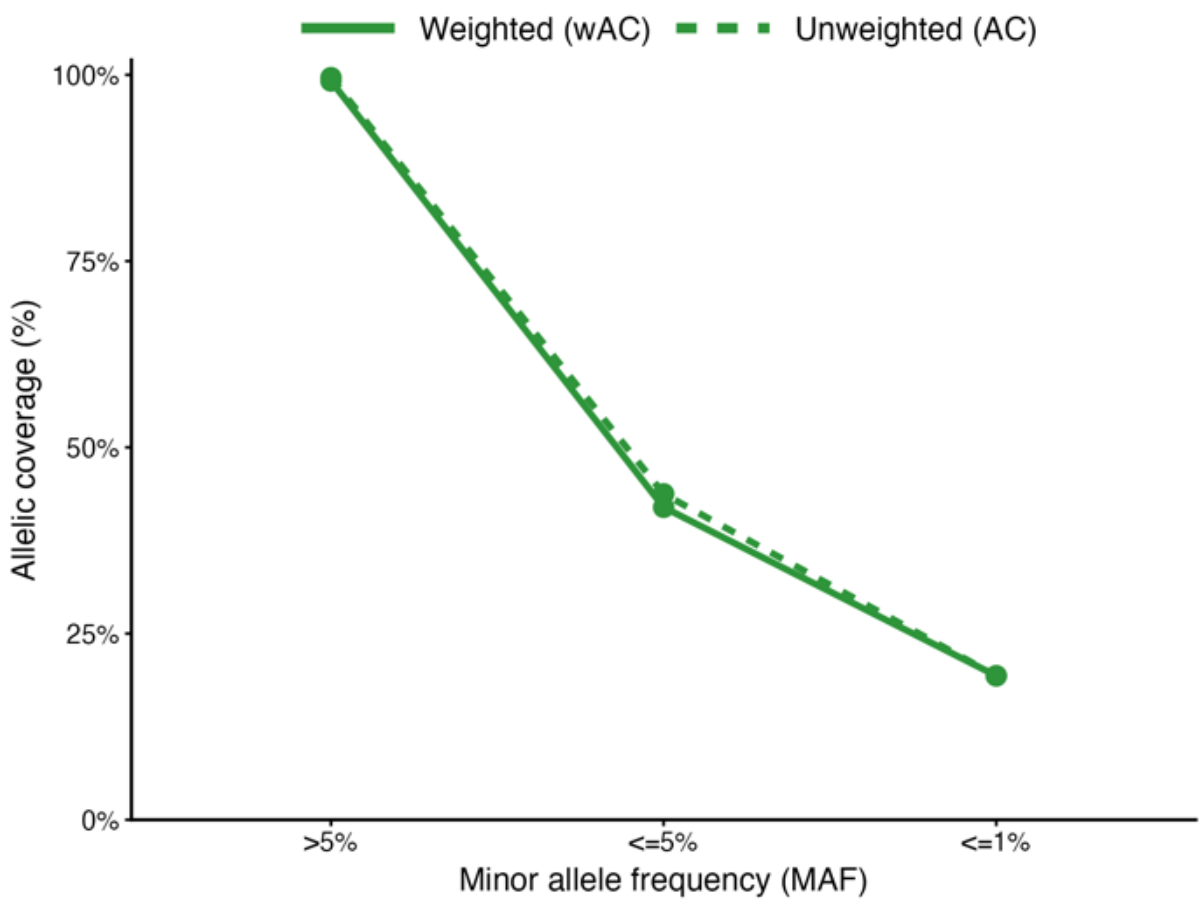

Supplementary Fig. 19 Rarity-weighted and unweighted allelic coverage of wild-tree alleles in cultivation.

Weighted (wAC; solid line) and unweighted (AC; dashed line) allelic coverage across three minor allele frequency (MAF) thresholds, showing the proportion of wild-tree variants captured by the cultivated population, based on the full dataset (n = 147). The wild-tree set comprised 33 individuals (n = 33) and the cultivated set comprised 50 individuals (n = 50).

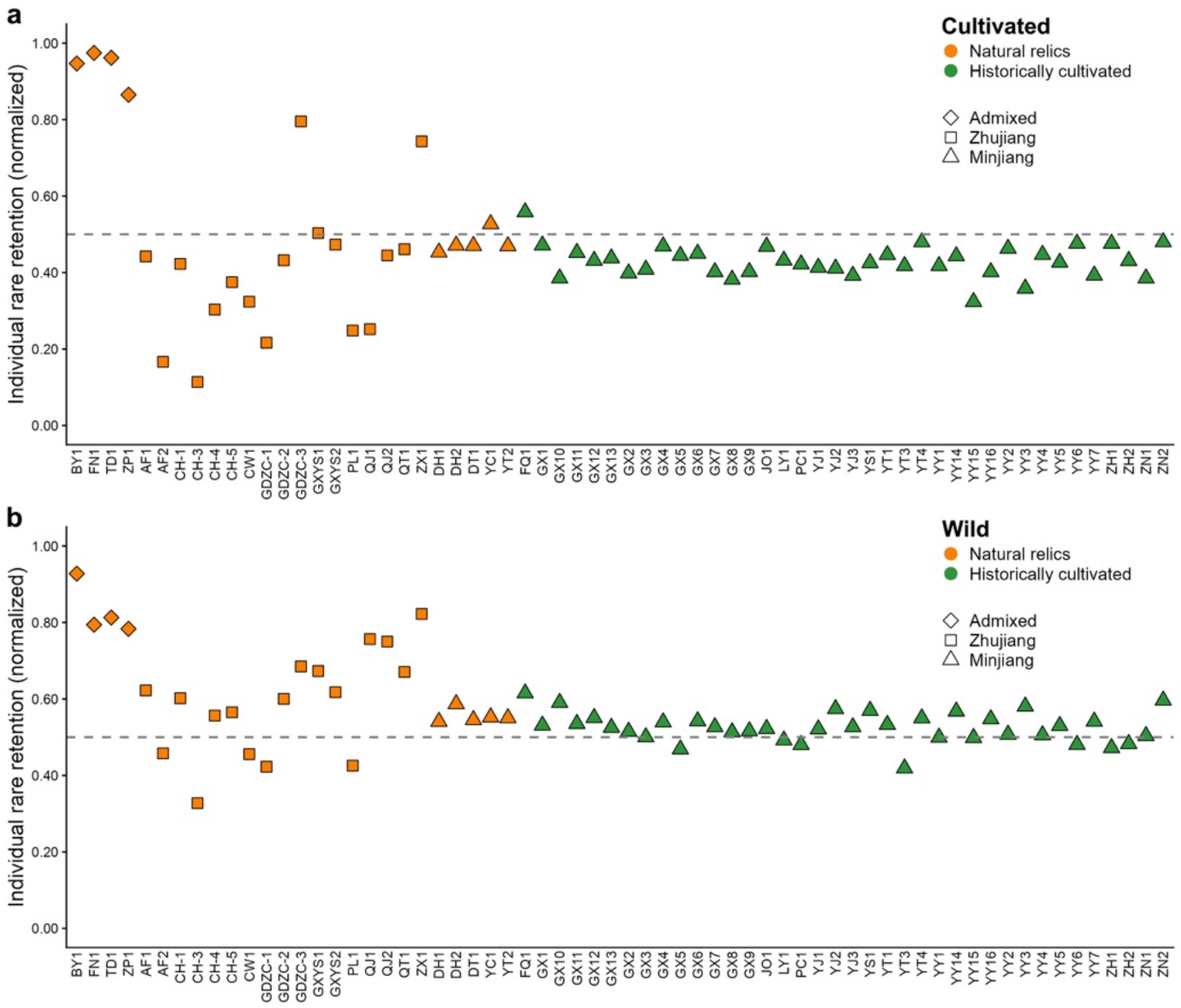

Supplementary Fig. 20 Individual rare retention (IRR) of ancient trees relative to cultivated and wild populations.

Individual rare retention index (IRR; normalized to 0–1) for all ancient trees (n = 64), calculated relative to the cultivated population (**a**) and the wild population (**b**), using the full dataset (n = 147). Higher IRR values indicate rare alleles present in ancient trees that are poorly represented in non-ancient populations.

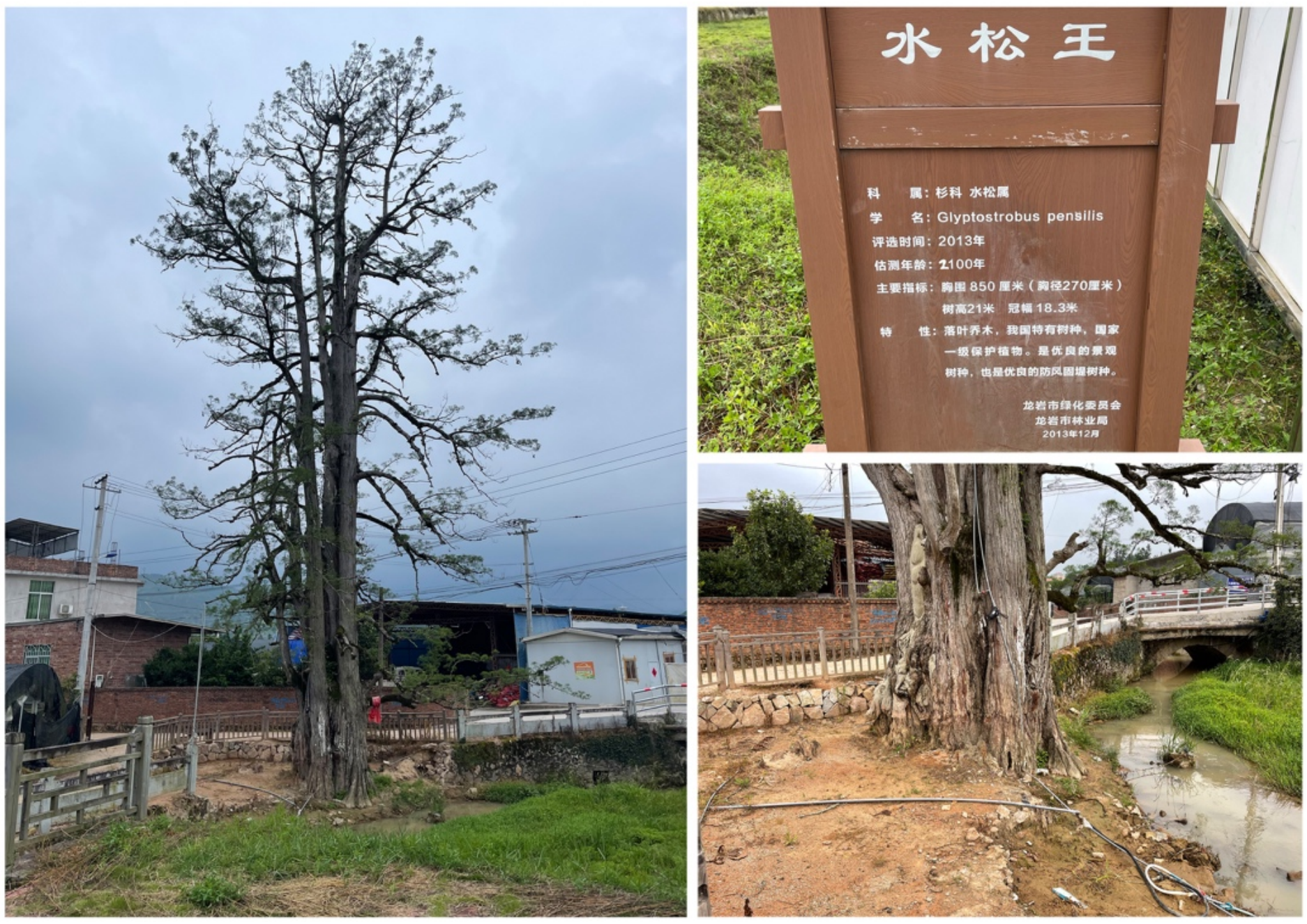

Supplementary Fig. 21 Example of a formally protected ancient *G. pensilis* individual with on-site conservation signage.

The tree, located in Zhangping County, Fujian Province, China, has an estimated age based on measurements conducted by the local Forestry Bureau in 2013. In our field survey in 2023, the tree was ~21 m tall with a crown spread of ~5 × 6 m and a diameter at breast height (DBH) of 2.52 m.

References

1. Manichaikul, A., Mychaleckyj J. C., Rich S. S., Daly K., Sale M. & Chen W. M. Robust relationship inference in genome-wide association studies. *Bioinformatics* **26**, 2867–2873 (2010).
2. Liu, B., et al. Estimation of genomic characteristics by analyzing k-mer frequency in de novo genome project. *arXiv*, 1308.2012 (2013).
3. Cheng, H., Concepcion G. T., Feng X., Zhang H. & Li H. Haplotype-resolved de novo assembly using phased assembly graphs with hifiasm. *Nat. Methods* **18**, 170–175 (2021).
4. Li, H. & Durbin R. Fast and accurate short read alignment with Burrows-Wheeler transform. *Bioinformatics* **25**, 1754–1760 (2009).
5. Zhang, X., Zhang S., Zhao Q., Ming R. & Tang H. Assembly of allele-aware, chromosomal-scale autopolyploid genomes based on Hi-C data. *Nat. Plants* **5**, 833–845 (2019).
6. Manni, M., Berkeley M. R., Seppey M., Simao F. A. & Zdobnov E. M. Busco update: Novel and streamlined workflows along with broader and deeper phylogenetic coverage for scoring of eukaryotic, prokaryotic, and viral genomes. *Mol. Biol. Evol.* **38**, 464–4654 (2021).
7. Wu, J. J., Han Y. W., Lin C. F., Cai J. & Zhao Y. P. Benchmarking gene set of gymnosperms for assessing genome and annotation completeness in BUSCO. *Hortic. Res.* **10**, uhad165 (2023).
8. Li, H., et al. The Sequence Alignment/Map format and SAMtools. *Bioinformatics* **25**, 2078–2079 (2009).
9. Ou, S. & Jiang N. LTR_retriever: a highly accurate and sensitive program for identification of long terminal repeat retrotransposons. *Plant Physiol.* **176**, 1410–1422 (2018).
10. Ou, S., et al. Benchmarking transposable element annotation methods for creation of a streamlined, comprehensive pipeline. *Genome Biol.* **20**, 275 (2019).
11. Smit, A. F. A., Hubley R. & Green P. RepeatMasker Home Page.). https://www.repeatmasker.org (2022).
12. Kim, D., Paggi J. M., Park C., Bennett C. & Salzberg S. L. Graph-based genome alignment and genotyping with HISAT2 and HISAT-genotype. *Nat. Biotechnol.* **37**, 907–915 (2019).
13. Bruna, T., Hoff K. J., Lomsadze A., Stanke M. & Borodovsky M. BRAKER2: automatic eukaryotic genome annotation with GeneMark-EP plus and AUGUSTUS supported by a protein database. *NAR Genomics Bioinf.* **3**, lqaa108 (2021).
14. Lomsadze, A., Burns P. D. & Borodovsky M. Integration of mapped RNA-Seq reads into automatic training of eukaryotic gene finding algorithm. *Nucleic Acids Res.* **42**, e119 (2014).
15. Haas, B. J., et al. Automated eukaryotic gene structure annotation using EVidenceModeler and the program to assemble spliced alignments. *Genome Biol.* **9**, R7 (2008).
16. Fu, L. M., Niu B. F., Zhu Z. W., Wu S. T. & Li W. Z. CD-HIT: accelerated for

clustering the next-generation sequencing data. *Bioinformatics* **28**, 3150–3152 (2012).

17. Pertea, M., Pertea G. M., Antonescu C. M., Chang T. C., Mendell J. T. & Salzberg S. L. StringTie enables improved reconstruction of a transcriptome from RNA-seq reads. *Nat. Biotechnol.* **33**, 290–295 (2015).
18. Holt, C. & Yandell M. MAKER2: an annotation pipeline and genome-database management tool for second-generation genome projects. *BMC Bioinformatics* **12**, 491 (2011).
19. Korf, I. Gene finding in novel genomes. *BMC Bioinformatics* **5**, 59 (2004).
20. Haas, B. J., et al. Improving the *Arabidopsis* genome annotation using maximal transcript alignment assemblies. *Nucleic Acids Res.* **31**, 5654–5666 (2003).
21. Jones, P., et al. InterProScan 5: genome-scale protein function classification. *Bioinformatics* **30**, 1236–1240 (2014).
22. Potter, S. C., Luciani A., Eddy S. R., Park Y., Lopez R. & Finn R. D. HMMER web server: 2018 update. *Nucleic Acids Res.* **46**, W200–W204 (2018).
23. Fu, F., et al. The *Metasequoia* genome and evolutionary relationships among redwoods. *Plant Commun.* **4**, 100643 (2023).
24. Niu, S., et al. The Chinese pine genome and methylome unveil key features of conifer evolution. *Cell* **185**, 204–217 (2022).
25. Liu, Y., et al. The *Cycas* genome and the early evolution of seed plants. *Nat. Plants* **8**, 389–401 (2022).
26. Wang, Y., et al. MCScanX: a toolkit for detection and evolutionary analysis of gene synteny and collinearity. *Nucleic Acids Res.* **40**, e49 (2012).
27. Wang, D. P., Wan H. L., Zhang S. & Yu J. Gamma-MYN: a new algorithm for estimating Ka and Ks with consideration of variable substitution rates. *Biol. Direct* **4**, 20 (2009).
28. Wang, D., Zhang Y., Zhang Z., Zhu J. & Yu J. KaKs_Calculator 2.0: a toolkit incorporating gamma-series methods and sliding window strategies. *Genomics Proteomics Bioinf.* **8**, 77–80 (2010).
29. Emms, D. M. & Kelly S. OrthoFinder: solving fundamental biases in whole genome comparisons dramatically improves orthogroup inference accuracy. *Genome Biol.* **16**, 157 (2015).
30. Stamatakis, A. RAxML version 8: a tool for phylogenetic analysis and post-analysis of large phylogenies. *Bioinformatics* **30**, 1312–1313 (2014).
31. Yang, Z. PAML 4: phylogenetic analysis by maximum likelihood. *Mol. Biol. Evol.* **24**, 1586–1591 (2007).
32. Stull, G. W., et al. Gene duplications and phylogenomic conflict underlie major pulses of phenotypic evolution in gymnosperms. *Nat. Plants* **7**, 1015–1025 (2021).
33. Han, M. V., Thomas G. W., Lugo-Martinez J. & Hahn M. W. Estimating gene gain and loss rates in the presence of error in genome assembly and annotation using CAFE 3. *Mol. Biol. Evol.* **30**, 1987–1997 (2013).
34. Yu, G. C., Wang L. G., Han Y. Y. & He Q. Y. clusterProfiler: an R package for comparing biological themes among gene clusters. *OMICS* **16**, 284–287 (2012).

35. Thompson, E. A. Identity by descent: variation in meiosis, across genomes, and in populations. *Genetics* **194**, 301–326 (2013).

36. Fujino, T., et al. A chromosome-level genome assembly of a model conifer plant, the Japanese cedar, *Cryptomeria japonica* D. Don. *BMC Genomics* **25**, 1039 (2024).

37. Moriguchi, Y., et al. The construction of a high-density linkage map for identifying SNP markers that are tightly linked to a nuclear-recessive major gene for male sterility in *Cryptomeria japonica* D. Don. *BMC Genomics* **13**, 95 (2012).
