# Supplementary material for: Population history rather than tree age contributes to the evolutionary importance of ancient trees in an endangered conifer
Source: Nat Plants. 2026 Aug 11;12(8):1455–68. doi: 10.1038/s41477-026-02367-9 (PMC13481254; doi:10.1038/s41477-026-02367-9)
Supplement: Supplementary file 2 [file 41477_2026_2367_MOESM2_ESM.pdf]

nature portfolio

Corresponding author(s): Ming Kang, Martin Lascoux

Last updated by author(s): Jun 29, 2026

Reporting Summary

Nature Portfolio wishes to improve the reproducibility of the work that we publish. This form provides structure for consistency and transparency in reporting. For further information on Nature Portfolio policies, see our Editorial Policies and the Editorial Policy Checklist.

Statistics

For all statistical analyses, confirm that the following items are present in the figure legend, table legend, main text, or Methods section.

n/a	Confirmed	
☐	☒	The exact sample size (*n*) for each experimental group/condition, given as a discrete number and unit of measurement
☐	☒	A statement on whether measurements were taken from distinct samples or whether the same sample was measured repeatedly
☐	☒	The statistical test(s) used AND whether they are one- or two-sided<br>*Only common tests should be described solely by name; describe more complex techniques in the Methods section.*
☒	☐	A description of all covariates tested
☒	☐	A description of any assumptions or corrections, such as tests of normality and adjustment for multiple comparisons
☐	☒	A full description of the statistical parameters including central tendency (e.g. means) or other basic estimates (e.g. regression coefficient) AND variation (e.g. standard deviation) or associated estimates of uncertainty (e.g. confidence intervals)
☐	☒	For null hypothesis testing, the test statistic (e.g. *F*, *t*, *r*) with confidence intervals, effect sizes, degrees of freedom and *P* value noted<br>*Give P values as exact values whenever suitable.*
☒	☐	For Bayesian analysis, information on the choice of priors and Markov chain Monte Carlo settings
☒	☐	For hierarchical and complex designs, identification of the appropriate level for tests and full reporting of outcomes
☒	☐	Estimates of effect sizes (e.g. Cohen's *d*, Pearson's *r*), indicating how they were calculated

Our web collection on statistics for biologists contains articles on many of the points above.

Software and code

Policy information about availability of computer code

Data collection	No software or custom code was used for data collection
Data analysis	Illumina, Pacbio and Hi-C sequencing methods; GCE v1.0.2, hifiasm v0.16, ALLHiC, BUSCO v5.4.4, embryophyta_odb10 and gymnosperm_odb10 datasets, LTR_retriever v2.9.0, EDTA v2.0.0, HISAT2 v2.2.1, AUGUSTUS v3.5.0, MAKER2, BWA v0.7.17, SAMtools v1.19, RepeatMasker v4.1.4, BRAKER2 v2.1.6, GeneMark-ET, CD-HIT v4.6.8, StringTie, SNAP, PASA v2.5.2, SWISS-PROT, NCBI NR, KOG, and eggNOG databases, InterProScan v5, KEGG, MCScanX, KaKs_Calculator 2.0, OrthoFinder v0.4.0, RAxML v8.2.8, PAML, Tracer v1.4, CAFE v4.2.1, OrthoFinder v0.4.0, R package clusterProfiler, DNBSEQ-T7, Trimmomatic v0.32, GATK v4.0, VCFtools v0.1.13, KING v2.1, STRUCTURE v2.3.4, KFinder v1.0, SNPRelate v1.34.1, pixy, GENESIS, SVDquartets, PSMC, GONE, PLINK v1.9, fastsimcoal2 v2.8, easySFS.py, ANGSD, BCFtools v1.15, SnpEff v5.0, SIFT4G, Est-SFS.<br>The code used for the main analyses in this study is publicly available at:<br>https://github.com/Hickory01/Ancient-Trees-Conservation-Genomics-of-Chinese-Swamp-Cypress.git

For manuscripts utilizing custom algorithms or software that are central to the research but not yet described in published literature, software must be made available to editors and reviewers. We strongly encourage code deposition in a community repository (e.g. GitHub). See the Nature Portfolio guidelines for submitting code & software for further information.

Data

Policy information about availability of data

All manuscripts must include a data availability statement. This statement should provide the following information, where applicable:

- Accession codes, unique identifiers, or web links for publicly available datasets
- A description of any restrictions on data availability
- For clinical datasets or third party data, please ensure that the statement adheres to our policy

The raw sequence data reported in this study have been deposited in the National Genomics Data Center (NGDC; https://ngdc.cncb.ac.cn) under accession number PRJCA061991. The final genome assembly and genome annotation files are available at in figshare (https://doi.org/10.6084/m9.figshare.32824982.v1.). Source data are provided with this paper.

Research involving human participants, their data, or biological material

Policy information about studies with human participants or human data. See also policy information about sex, gender (identity/presentation), and sexual orientation and race, ethnicity and racism.

Reporting on sex and gender	*Use the terms sex (biological attribute) and gender (shaped by social and cultural circumstances) carefully in order to avoid confusing both terms. Indicate if findings apply to only one sex or gender; describe whether sex and gender were considered in study design; whether sex and/or gender was determined based on self-reporting or assigned and methods used. Provide in the source data disaggregated sex and gender data, where this information has been collected, and if consent has been obtained for sharing of individual-level data; provide overall numbers in this Reporting Summary. Please state if this information has not been collected. Report sex- and gender-based analyses where performed, justify reasons for lack of sex- and gender-based analysis.*
Reporting on race, ethnicity, or other socially relevant groupings	*Please specify the socially constructed or socially relevant categorization variable(s) used in your manuscript and explain why they were used. Please note that such variables should not be used as proxies for other socially constructed/relevant variables (for example, race or ethnicity should not be used as a proxy for socioeconomic status). Provide clear definitions of the relevant terms used, how they were provided (by the participants/respondents, the researchers, or third parties), and the method(s) used to classify people into the different categories (e.g. self-report, census or administrative data, social media data, etc.) Please provide details about how you controlled for confounding variables in your analyses.*
Population characteristics	*Describe the covariate-relevant population characteristics of the human research participants (e.g. age, genotypic information, past and current diagnosis and treatment categories). If you filled out the behavioural & social sciences study design questions and have nothing to add here, write "See above."*
Recruitment	*Describe how participants were recruited. Outline any potential self-selection bias or other biases that may be present and how these are likely to impact results.*
Ethics oversight	*Identify the organization(s) that approved the study protocol.*

Note that full information on the approval of the study protocol must also be provided in the manuscript.

Field-specific reporting

Please select the one below that is the best fit for your research. If you are not sure, read the appropriate sections before making your selection.

☐ Life sciences ☐ Behavioural & social sciences ☒ Ecological, evolutionary & environmental sciences

For a reference copy of the document with all sections, see nature.com/documents/nr-reporting-summary-flat.pdf

Ecological, evolutionary & environmental sciences study design

All studies must disclose on these points even when the disclosure is negative.

Study description	This study investigates whether the evolutionary importance of ancient trees reflects tree age per se or demographic lineage history in the endangered conifer Glyptostrobus pensilis (Chinese swamp cypress). We assembled and annotated a chromosome-level (~8 Gb) reference genome and generated population-scale whole-genome resequencing data from 147 individuals, including 64 ancient, 33 wild, and 50 cultivated individuals spanning its fragmented distribution. These genomic resources enabled us to evaluate how population history shapes the persistence and genetic irreplaceability of ancient individuals and to quantify the extent to which their genetic variation is retained in contemporary wild and cultivated populations.
Research sample	Sampling informations are reported in the Supplementary Tables 1–3.
Sampling strategy	A total of 147 individuals of Glyptostrobus pensilis (Chinese swamp cypress) were sampled across its fragmented distribution, including 64 ancient, 33 wild, and 50 cultivated trees. Two closely related species, Taxodium distichum and Cryptomeria japonica, were included as outgroups. Targeted sampling was applied to ancient trees due to their rarity and limited population size. In

	contrast, interval-based random sampling was conducted for wild and cultivated trees within the same sites to ensure comparable spatial representation. For each adult tree, six to ten young leaves were collected for DNA extraction and subsequent whole-genome sequencing.
Data collection	Plant material for each individual listed in Supplementary Table 1 was collected in the field by the first author (Wei-Ping Zhang), except for three Vietnamese samples, which were provided by our collaborator Van Sam Hoang (Vietnam National University of Forestry). DNA sequencing was performed by commercial providers (Novogene and BGI Genomics Technologies).
Timing and spatial scale	Empirical data has been collected 2023-2024 across southern China and Vietnam.
Data exclusions	n/a
Reproducibility	DNA isolation from leaf samples, as well as the germination experiment, was conducted once in this study. Most of our genomic analyses were repeated and cross-validated using multiple complementary methods.
Randomization	n/a
Blinding	Investigators were not blinded during data collection or analysis. Conservation categories (ancient, wild, or cultivated) and vitality status (Healthy, Moderate, Fair, Poor, or Dying) were assigned using standardized field criteria grounded in documented biological and ecological characteristics. The distinction between natural relic and historically introduced ancient trees was determined through population genomic analyses combined geographic context. Whole-genome analyses, including estimates of genetic diversity and genetic load, were performed using standardized bioinformatic pipelines to minimize potential bias.

Did the study involve field work? ☒ Yes ☐ No

Field work, collection and transport

Field conditions	Our fieldwork primarily focused on documenting species identification and distribution information (including geographic coordinates and elevation), conservation status and threat information, habitat characteristics (including evidence of anthropogenic disturbance), tree size (age-related informations, with DBH used as supplementary information), growth condition, phenology, fruit production, and evidence of natural regeneration in the field, as well as other taxonomic and morphological traits. Other environmental variables not directly relevant to the scope of this study were not included.
Location	The 147 individuals of Chinese swamp cypress and two outgroups investigated in this study were sampled from multiple provinces in southern China and one location in Vietnam. Detailed sampling locations are provided in Supplementary Tables 1–3.
Access & import/export	No import or export permits were required for this study. Vietnamese samples were provided by our collaborator Prof. Van Sam Hoang (Vietnam National University of Forestry) and were collected in accordance with applicable local regulations.
Disturbance	No habitat disturbance was cause by the leaf and fruit collection.

Reporting for specific materials, systems and methods

We require information from authors about some types of materials, experimental systems and methods used in many studies. Here, indicate whether each material, system or method listed is relevant to your study. If you are not sure if a list item applies to your research, read the appropriate section before selecting a response.

Materials & experimental systems

n/a	Involved in the study	
☒	☐	Antibodies
☒	☐	Eukaryotic cell lines
☒	☐	Palaeontology and archaeology
☒	☐	Animals and other organisms
☒	☐	Clinical data
☒	☐	Dual use research of concern
☐	☒	Plants

Methods

n/a	Involved in the study	
☒	☐	ChIP-seq
☒	☐	Flow cytometry
☒	☐	MRI-based neuroimaging

Dual use research of concern

Policy information about dual use research of concern

Hazards

Could the accidental, deliberate or reckless misuse of agents or technologies generated in the work, or the application of information presented in the manuscript, pose a threat to:

No	Yes	
☒	☐	Public health
☒	☐	National security
☒	☐	Crops and/or livestock
☒	☐	Ecosystems
☒	☐	Any other significant area

Experiments of concern

Does the work involve any of these experiments of concern:

No	Yes	
☒	☐	Demonstrate how to render a vaccine ineffective
☒	☐	Confer resistance to therapeutically useful antibiotics or antiviral agents
☒	☐	Enhance the virulence of a pathogen or render a nonpathogen virulent
☒	☐	Increase transmissibility of a pathogen
☒	☐	Alter the host range of a pathogen
☒	☐	Enable evasion of diagnostic/detection modalities
☒	☐	Enable the weaponization of a biological agent or toxin
☒	☐	Any other potentially harmful combination of experiments and agents

Plants

Seed stocks	Adult leaf and seed samples were collected in the field from individuals representing all three conservation types (64 ancient, 33 wild and 50 cultivated trees). Voucher information for each sampled individual is provided in Supplementary Tables 1–3, and corresponding specimens have been deposited in the CAS Herbarium.
Novel plant genotypes	n/a
Authentication	n/a
