# Supplementary material for: Population history rather than tree age contributes to the evolutionary importance of ancient trees in an endangered conifer
Source: Nat Plants. 2026 Aug 11;12(8):1455–68. doi: 10.1038/s41477-026-02367-9 (PMC13481254; doi:10.1038/s41477-026-02367-9)
Supplement: Supplementary file 3 [file 41477_2026_2367_MOESM3_ESM.pdf]

nature portfolio

Peer Review File

Population history rather than tree age contributes to the evolutionary importance of ancient trees in an endangered conifer

Corresponding Author: Professor Ming Kang

Version 0:

Reviewer comments:

Reviewer #1

(Comments for the Author)
Review of Zhang et al - submitted to Nature Plants

I think that this is a very nice paper and one that will fit nicely in Nature Plants. I learned a lot from reading this paper. Overall, I can't say that I'm overly surprised by the result that the ancient trees have similar genetic variation to the young trees, but that's not a knock against the paper. On the contrary, I agree with the authors that there is a general expectation that ancient trees would be reservoirs for genetic diversity, so this paper is tackling an open question. The data looks excellent and analyses are done well. This work represents a really nice example of conservation genomics, the authors integrate much of the recent literature in population genomics in their approach.

The biggest issue I have with the paper is whether this species is actually an ideal test case for the question of whether old individuals possess beneficial alleles. It is argued in the Abstract (31-34), Introduction (Lines 69-70) and extensively in the Discussion (but predominantly on lines 346-347) that this notion can be rejected. However, after reading the paper I'm not sure that I agree.

There are numerous issues that are raised in the Discussion about the potential relaxation of selection in the ancient trees that they studied. A large fraction of the ancient trees analysed in this study have been cared for by humans, so it seems plausible that natural selection may have been relaxed for those individuals. In addition, the extremely limited population sizes in the species, which seem to have been very low for at least the last 5000 years would have led to high levels of drift. This history of high rates of drift may mean that the populations are not particularly well adapted. This is more or less supported by the pi0/pi4 results that the authors note as being at the upper end of what has been reported for plants.

The idea that the authors are arguing against is that old trees are reservoirs of beneficial alleles as they have withstood the test of time. However, if a population is not well adapted in the first place, then there would likely not be much of a difference between ancient and young trees. Furthermore, if the ancient trees are alive because humans have kept them alive, then they are not exemplars of the idea that ancient/old growth trees are reservoirs of beneficial alleles. I don't think that this undercuts the high quality of the paper, but I do think that this framing of the paper should be tempered. If you looked at, say, Norway Spruce or Douglas-fir, species with much larger population sizes, would you expect the same result? I'm not convinced that the results would be that different, but without testing it we cannot be sure. This comment could be addressed by some re-wording and re-framing.

I have some other questions about the paper, some big, some small.

Major comments:

What is an ancient tree? It is not described in the Intro or the results what actually constitutes an ancient tree. This is given in the methods (age more than 100 years), but for the sake of readability I think that this should be mentioned somewhere near the beginning. I couldn't find a table or anything with age estimates for all the samples. It seems that there may be a considerable difference between a tree that is 2100 years old and one that is 100 years old. For the results on vitality, a little more justification would be good. It seems strange to me to incorporate anthropogenic disturbance into a vitality metric. Such a disturbance doesn't seem like it is really in the spirit of what the authors are trying to measure - whether ancient trees are specifically well adapted.

What fraction of the ancient trees were maintained as fengshui trees (or maintained by humans in other ways)? Basically my

question is what is the expected lifespan of this species in the absence of human intervention? Most of the wild population were classified as healthy, but these are not managed. Maybe it's just the case that typical causes of mortality have been eliminated for the ancient trees. I appreciate that that is largely speculative, but this does bear on the overall thrust of the paper. The main message is that ancient trees don't harbour important genetic variation. But if the ancient trees are only ancient because humans have prevented them from dying, maybe this species is not the best test of this theory. What is the distribution of DBH and vitality for the natural relics versus the historically cultivated ancients?

Supp Fig S8 - On the PCA - PC1 was dominated by the placement of two of the admixed individuals. Were those individuals different in any other ways? Are they the two individuals that form the outermost clade in Figure 2d?

PSMC - what are we to infer from the finding that the maximum depth differs among the different groups? What is the relevance of this depth and it's correlation with heterozygosity. Of course the higher the heterozygosity the longer to coalescence (i.e. neutral coalescence time is expected to be 2Ne and nucleotide diversity/heterozygosity is expected to be 4Neu). If it's just that, I don't think that it needs to be presented alongside the major results. If it's not, the authors should give some description about what they want us to take away from that.

Additionally, I generally think that relating PSMC results to geological time can be a bit tricky given how assumption laden the estimate of generation time and mutation rate is. It would be good to have that uncertainty reflected in the description of how the PSMC results relate to the geological record.

Private alleles - one of the signature that we might expect for the ancient trees is that they possess genetic variation that is not present in the younger trees. Aside from the admixed individuals, Figure 2e shows that the ancient trees don't seem to have any greater proportion of private alleles than the young ones. That seems worth a mention in the text. I appreciate that this is returned to in the section beginning on Lines 251.

Mutation rate - the mutation rate that is stated in the methods is rather low (line536). The authors also used different estimates for fastsimcoal and PSMC. Did I miss something in why these are so different? As a side note, Hanlon et al estimate somatic mutation rates in sitka spuce and got an estimate of ~2.7e-8/bp/generation (which was higher than angiosperms) - that may provide a more empirical justification for the mutation rate choice than the citation given, which was based on divergence data.

Nucleotide diversity - no statement of uncertainty is given in the text, or in Figure 4a. Perhaps bootstrap samples of the 200kbp windows could be done to obtain empirical estimates of error? I don't think that this will change the conclusions from lines 199-206, but would probably strengthen the authors argument that there is little difference between the groups.

Landscape of nucleotide diversity - for people like me who find it useful to look at these graphs, it would be nice to include versions of 4e for the other 10 chromosomes. Throwing these into the supplement would be sufficient.

Minor points

Lines 37-47 - another aspect of ancient trees is that their typically large size supports a diverse ecosystem of micro and macro organisms. There is also the notion that ancient trees may foster mycorrhizal connections that support forest ecosystems in other ways. I leave it to the authors to take from this what they want, but suggest that a little more on the potential importance of ancient trees beyond their genetic diversity would be a good addition to the introduction.

Line 423 - How does Illumina read mapping demonstrate assembly quality in this context?

Figure 2e - is the colour code the same as in the previous panels?

Please excuse any typos or grammatical errors.

Reviewer #2

(Comments for the Author)
The authors address a very important question: do ancient trees retain higher genetic diversity or higher amounts of rare alleles, supporting the argument that they are disproportionately important to population survival. I applaud the authors for taking on this question.

However, the study never makes clear how “ancient” individuals are defined, except by DBH. Yet (1) we never are shown any data suggesting a tight correlation between DBH and age, a correlation that can be quite loose because of plastic growth rates; and (2) the DBH distribution overlaps between the ancient and non-ancient individuals (Fig 1d), demonstrating that some other criterion is used to identify ancient individuals. I did not see any description of how this categorization was made, leaving me to wonder whether ancient trees were defined based on tradition or subjective criteria. In light of this, I don’t know how to interpret the central claims of the manuscript.

Additionally, I have some methodological concerns and specific points that bear addressing:

22: define “ancient” on first use (even in abstract, in my opinion); also, are the “ancient” trees spread across both cultivated

and wild? Surely “ancient” is not a category distinct from “wild” and “cultivated”
55: I’m not sure what concept you have in mind by “genetically eroded survivors”. I think you are talking about individual trees; do you just mean maladapted? Or low-heterozygosity? I assume you mean one or both of these, but in either case “genetically eroded” doesn’t clearly convey to me what you might have in mind.
66-68: It is true that “size-based protection can fail to safeguard genuinely old individuals”, but it does not follow that “a direct genomic test is needed to evaluate whether ancient trees retain irreplaceable genetic variation.” These are two separate ideas that should be separated into separate sentences.
73: Delete “Once”
85: “more than 100 ancient, wild and cultivated individuals” – this doesn’t align with the number of individuals you provide in the abstract or the results
95-96: as in the abstract, you need to make clear that the ancient trees are all wild (if they are), and that additionally you sampled 33 wild trees not viewed as ancient. Also, we still don’t know what you mean by “ancient”.
99: Some information needed here on what went into vitality assessment. The information in the supplement is good; abstract it to a few sentences for the main text.
99-100. is the low mean level of vitality in the ancient individuals just a consequence of the fact that they are old?
104-106. Here we learn that dbh is used as a proxy for age; this information should be indicated up front. It is not a response in your study, but a predictor (the only proxy I gather of ancientness).
106—109. This is a bit circular: DBH is your proxy for age, age is associated with vitality, vitality is associated with DBH. Probably only report on 2 of these, not 3 (as though we are getting three pieces of distinct information; the association betw dbh and age is assumed, and the association between vitality and dbh / age is observed.
138-140. The total number of ancient individuals in each province is less interesting than the percent of individuals sampled in each province that are ancient; and even that’s of questionable use, b/c we don’t know how you decided how many individuals to sample from each population.
147-148: “these patterns allowed us to 148 distinguish 26 natural relics from 38 historically introduced ancient trees” --- not clear how this was done. Even looking at Supplemental Fig. 7, I’m not sure how you inferred this.
177-179: this is highly circular: PSMC uses heterozygosity to infer demographic history, so the finding of a high correlation between heterozygosity and depth ($r2 = 0.975$) is exactly what is expected.
186—188: higher Ne is an expected outcome of admixture; so I’m not sure what this result is intended to tell us.
201—204. Admixed lineages will of course have higher heterozygosity, so this is not so much a finding as a report of the known relationship between heterozygosity and admixture; then there is only a ca. 2-fold difference in pi between Zhujiang and Minjiang, which I wouldn’t think qualifies as “substantial.”
301-305. I think this is the first time we get a clear idea that “genomic erosion” is being used to mean “erosion of genomic diversity.” If that’s the use throughout, make that clear up front.
311-316. As discussed above, I don’t find this “lineage” effect persuasive. The admixed individuals are expected to have high diversity, so that’s not much of a finding; and the difference between the unadmixed lineages seems pretty minimal.
Figs 4h, i --- these are hard to interpret but very important.

Reviewer #3

(Comments for the Author)
This is an interesting study that tests the long-standing view that old trees are more genetically diverse and unique. The authors compare genomic data from 64 ancient, 33 wild, and 50 cultivated individuals of Glyptostrobus pensilis and find that population lineage identity, rather than individual age, contributes most to genome-wide diversity. This conclusion challenges the conventional assumption that long-lived individuals are inherently genetically irreplaceable and has potential implications for the conservation of this endangered species. The study is comprehensive, with relatively large-scale sampling and analyses from multiple perspectives.

I am not a specialist in genomic diversity, but I am familiar with dendrochronology and ancient trees. So, I only have several concerns regarding the framing and methodology, which may affect the strength of the conclusions.

Major comments
1. Overstatement of novelty
The manuscript states that “a central assumption… remains surprisingly untested,” which appears overstated. Several previous studies have addressed related questions, including:
1. Mosseler, A., Major, J. E., & Rajora, O. P. (2003). Old-growth red spruce forests as reservoirs of genetic diversity and reproductive fitness. Theoretical and Applied Genetics, 106(5), 931-937.
2. Hanlon, V. C., Otto, S. P., & Aitken, S. N. (2019). Somatic mutations substantially increase the per-generation mutation rate in the conifer Picea sitchensis. Evolution Letters, 3(4), 348-358.
3. Díez, C. M., Trujillo, I., Barrio, E., Belaj, A., Barranco, D., & Rallo, L. (2011). Centennial olive trees as a reservoir of genetic diversity. Annals of Botany, 108(5), 797-807.

These studies, although using different approaches, generally support the idea that old trees can act as reservoirs of genetic diversity, which contrasts with the conclusions of the present manuscript. While the genomic methods used here are more advanced, the authors should better acknowledge this body of work and more carefully position their contribution relative to existing studies.

2. Lack of direct age estimation
The ages of the 147 sampled trees are not directly quantified. Instead, diameter at breast height (DBH) is used as a proxy to distinguish ancient and wild trees. Although Tang et al. (ref. 18) reported a positive relationship between DBH and age, this relationship is based on a case study from Fujian Province and may not be broadly applicable. Given that G. pensilis is a relatively fast-growing species, large individuals in other regions may still be relatively young. As a result, conclusions related to individual age are not well supported. More importantly, the study conflates conservation categories (ancient, wild, cultivated) with tree age. To directly test the central hypothesis, it would be preferable to incorporate dendrochronological data or other reliable age estimates and explicitly analyses the relationship between individual age and genomic diversity.

3. Interpretation of population history versus age effects
I agree that population history is likely a major driver of genomic diversity. However, this conclusion would be more convincing if the authors compared individuals with similar population histories but different ages within the same region. Such a design would allow a more direct test of the relative importance of age versus lineage.

4. Limitations of species choice and sample size
The relatively small sample size may limit the robustness of the conclusions. In addition, G. pensilis rarely exceeds ~150 years (ref. 18), meaning that the sampled “old” trees may not represent truly long-lived individuals. Extremely old trees (e.g., >500 years) are often survivors from historically large and genetically diverse populations and may represent “lottery winners” (ref 2). In contrast, G. pensilis populations have been strongly impacted by human activities and may largely consist of younger cohorts. Therefore, the observed patterns could be strongly influenced by population history and genetic drift, rather than providing a definitive test of whether very old trees harbor unique genetic variation.

Given these limitations, the conservation implications should be framed more cautiously.

Minor comments
Lines 91–112: The section describing poorer vitality in ancient trees is not directly relevant to the main research question and could be substantially shortened.
Lines 370–371: The information presented appears to be based on anecdotal sources and may not be reliable; it should be revised or removed.

Decision Letter:

15th April 2026

Dear Professor Kang,

Your Article, "Population history rather than individual age contributes to the evolutionary importance of ancient trees in an endangered conifer" has now been seen by three referees. You will see from their comments copied below that while they find your work of considerable potential interest, they have raised quite substantial concerns that must be addressed. In light of these comments, we cannot accept the manuscript for publication, but would be very interested in considering a revised version that addresses these serious concerns.

As you can read below, Referees #1 and #3 are quite positive about your study and find it solid, novel, and significant, whereas Referee #2 is somewhat less enthusiastic. Overall, the main points include providing a clear definition of “ancient,” reframing and rewording the text to temper your claims, reorganizing the manuscript and adding a more detailed description of the results, improving the introduction, and including additional analyses to verify the robustness of some of the results. We hope you will find the referees’ comments useful as you decide how to proceed. Please do not hesitate to get in touch if you would like to discuss these issues further.

We therefore invite you to revise your manuscript considering all reviewer and editor comments. Please highlight changes in the manuscript text file and do not forget that most responses to reviewers should translate into changes in the text or figures. There is no need for a very precise version with all exact edits; just highlight the phrases you have modified or added to help editors and reviewers spot them.

We are committed to providing a fair and constructive peer-review process. Do not hesitate to contact us if there are specific requests from the reviewers that you believe are technically impossible or unlikely to yield a meaningful outcome.

If revising your manuscript please:

* Include a "Response to referees" document detailing, point-by-point, how you addressed each referee comment. If no action was taken to address a point, you must provide a compelling argument. This response will be sent back to the referees along with the revised manuscript.

* If you have not done so already we suggest that you begin to revise your manuscript so that it conforms to our Article format instructions at http://www.nature.com/nplants/info/final-submission. Refer also to any guidelines provided in this letter.

* Pay close attention to our href="https://www.nature.com/nature-portfolio/editorial-policies/image-integrity">Digital Image Integrity Guidelines. and to the following points. Please ensure that you retain unprocessed data and metadata files after

publication, ideally archiving data in perpetuity. These may be requested during the peer review and production process or after publication if any issues arise.

* Include a revised version of any required reporting checklist. It will be available to referees to aid in their evaluation if the manuscript goes back for peer review. A revised checklist is essential for re-review of the paper.

You may use the link below to submit your revised manuscript and related files:

Link Redacted

Note: This URL links to your confidential home page and associated information about manuscripts you may have submitted, or that you are reviewing for us. If you wish to forward this email to co-authors, please delete the link to your homepage.

If you wish to submit a suitably revised manuscript we would hope to receive it within 6 months. If you cannot send it within this time, please let us know. We will be happy to consider your revision so long as nothing similar has been accepted for publication at Nature Plants or published elsewhere.

Thank you for the opportunity to review your work.

Sincerely,

Nature Plants is committed to improving transparency in authorship. As part of our efforts in this direction, we are now requesting that all authors identified as 'corresponding author' on published papers create and link their Open Researcher and Contributor Identifier (ORCID) with their account on the Manuscript Tracking System (MTS), prior to acceptance. This applies to primary research papers only. ORCID helps the scientific community achieve unambiguous attribution of all scholarly contributions. You can create and link your ORCID from the home page of the MTS by clicking on 'Modify my Springer Nature account'. For more information please visit please visit www.springernature.com/orcid.

Please do not hesitate to contact me if you have any questions or would like to discuss the required revisions further.

****

Reviewers' Comments:

Reviewer #1 (Comments for the Author):

Review of Zhang et al - submitted to Nature Plants

I think that this is a very nice paper and one that will fit nicely in Nature Plants. I learned a lot from reading this paper. Overall, I can't say that I'm overly surprised by the result that the ancient trees have similar genetic variation to the young trees, but that's not a knock against the paper. On the contrary, I agree with the authors that there is a general expectation that ancient trees would be reservoirs for genetic diversity, so this paper is tackling an open question. The data looks excellent and analyses are done well. This work represents a really nice example of conservation genomics, the authors integrate much of the recent literature in population genomics in their approach.

The biggest issue I have with the paper is whether this species is actually an ideal test case for the question of whether old individuals possess beneficial alleles. It is argued in the Abstract (31-34), Introduction (Lines 69-70) and extensively in the Discussion (but predominantly on lines 346-347) that this notion can be rejected. However, after reading the paper I'm not sure that I agree.

There are numerous issues that are raised in the Discussion about the potential relaxation of selection in the ancient trees that they studied. A large fraction of the ancient trees analysed in this study have been cared for by humans, so it seems plausible that natural selection may have been relaxed for those individuals. In addition, the extremely limited population sizes in the species, which seem to have been very low for at least the last 5000 years would have led to high levels of drift. This history of high rates of drift may mean that the populations are not particularly well adapted. This is more or less supported by the pi0/pi4 results that the authors note as being at the upper end of what has been reported for plants.

The idea that the authors are arguing against is that old trees are reservoirs of beneficial alleles as they have withstood the test of time. However, if a population is not well adapted in the first place, then there would likely not be much of a difference between ancient and young trees. Furthermore, if the ancient trees are alive because humans have kept them alive, then they are not exemplars of the idea that ancient/old growth trees are reservoirs of beneficial alleles. I don't think that this undercuts the high quality of the paper, but I do think that this framing of the paper should be tempered. If you looked at, say, Norway Spruce or Douglas-fir, species with much larger population sizes, would you expect the same result? I'm not convinced that the results would be that different, but without testing it we cannot be sure. This comment could be addressed by some re-wording and re-framing.

I have some other questions about the paper, some big, some small.

Major comments:

What is an ancient tree? It is not described in the Intro or the results what actually constitutes an ancient tree. This is given in the methods (age more than 100 years), but for the sake of readability I think that this should be mentioned somewhere near the beginning. I couldn't find a table or anything with age estimates for all the samples. It seems that there may be a considerable difference between a tree that is 2100 years old and one that is 100 years old. For the results on vitality, a little more justification would be good. It seems strange to me to incorporate anthropogenic disturbance into a vitality metric. Such a disturbance doesn't seem like it is really in the spirit of what the authors are trying to measure - whether ancient trees are specifically well adapted.

What fraction of the ancient trees were maintained as fengshui trees (or maintained by humans in other ways)? Basically my question is what is the expected lifespan of this species in the absence of human intervention? Most of the wild population were classified as healthy, but these are not managed. Maybe it's just the case that typical causes of mortality have been eliminated for the ancient trees. I appreciate that that is largely speculative, but this does bear on the overall thrust of the paper. The main message is that ancient trees don't harbour important genetic variation. But if the ancient trees are only ancient because humans have prevented them from dying, maybe this species is not the best test of this theory. What is the distribution of DBH and vitality for the natural relics versus the historically cultivated ancients?

Supp Fig S8 - On the PCA - PC1 was dominated by the placement of two of the admixed individuals. Were those individuals different in any other ways? Are they the two individuals that form the outermost clade in Figure 2d?

PSMC - what are we to infer from the finding that the maximum depth differs among the different groups? What is the relevance of this depth and it's correlation with heterozygosity. Of course the higher the heterozygosity the longer to coalescence (i.e. neutral coalescence time is expected to be 2Ne and nucleotide diversity/heterozygosity is expected to be 4Neu). If it's just that, I don't think that it needs to be presented alongside the major results. If it's not, the authors should give some description about what they want us to take away from that.

Additionally, I generally think that relating PSMC results to geological time can be a bit tricky given how assumption laden the estimate of generation time and mutation rate is. It would be good to have that uncertainty reflected in the description of how the PSMC results relate to the geological record.

Private alleles - one of the signature that we might expect for the ancient trees is that they possess genetic variation that is not present in the younger trees. Aside from the admixed individuals, Figure 2e shows that the ancient trees don't seem to have any greater proportion of private alleles than the young ones. That seems worth a mention in the text. I appreciate that this is returned to in the section beginning on Lines 251.

Mutation rate - the mutation rate that is stated in the methods is rather low (line536). The authors also used different estimates for fastsimcoal and PSMC. Did I miss something in why these are so different? As a side note, Hanlon et al estimate somatic mutation rates in sitka spuce and got an estimate of ~2.7e-8/bp/generation (which was higher than angiosperms) - that may provide a more empirical justification for the mutation rate choice than the citation given, which was based on divergence data.

Nucleotide diversity - no statement of uncertainty is given in the text, or in Figure 4a. Perhaps bootstrap samples of the 200kbp windows could be done to obtain empirical estimates of error? I don't think that this will change the conclusions from lines 199-206, but would probably strengthen the authors argument that there is little difference between the groups.

Landscape of nucleotide diversity - for people like me who find it useful to look at these graphs, it would be nice to include versions of 4e for the other 10 chromosomes. Throwing these into the supplement would be sufficient.

Minor points

Lines 37-47 - another aspect of ancient trees is that their typically large size supports a diverse ecosystem of micro and macro organisms. There is also the notion that ancient trees may foster mycorrhizal connections that support forest ecosystems in other ways. I leave it to the authors to take from this what they want, but suggest that a little more on the potential importance of ancient trees beyond their genetic diversity would be a good addition to the introduction.

Line 423 - How does Illumina read mapping demonstrate assembly quality in this context?

Figure 2e - is the colour code the same as in the previous panels?

Please excuse any typos or grammatical errors.

Reviewer #2 (Comments for the Author):

The authors address a very important question: do ancient trees retain higher genetic diversity or higher amounts of rare

alleles, supporting the argument that they are disproportionately important to population survival. I applaud the authors for taking on this question.

However, the study never makes clear how “ancient” individuals are defined, except by DBH. Yet (1) we never are shown any data suggesting a tight correlation between DBH and age, a correlation that can be quite loose because of plastic growth rates; and (2) the DBH distribution overlaps between the ancient and non-ancient individuals (Fig 1d), demonstrating that some other criterion is used to identify ancient individuals. I did not see any description of how this categorization was made, leaving me to wonder whether ancient trees were defined based on tradition or subjective criteria. In light of this, I don’t know how to interpret the central claims of the manuscript.

Additionally, I have some methodological concerns and specific points that bear addressing:

22: define “ancient” on first use (even in abstract, in my opinion); also, are the “ancient” trees spread across both cultivated and wild? Surely “ancient” is not a category distinct from “wild” and “cultivated”
55: I’m not sure what concept you have in mind by “genetically eroded survivors”. I think you are talking about individual trees; do you just mean maladapted? Or low-heterozygosity? I assume you mean one or both of these, but in either case “genetically eroded” doesn’t clearly convey to me what you might have in mind.
66-68: It is true that “size-based protection can fail to safeguard genuinely old individuals”, but it does not follow that “a direct genomic test is needed to evaluate whether ancient trees retain irreplaceable genetic variation.” These are two separate ideas that should be separated into separate sentences.
73: Delete “Once”
85: “more than 100 ancient, wild and cultivated individuals” – this doesn’t align with the number of individuals you provide in the abstract or the results
95-96: as in the abstract, you need to make clear that the ancient trees are all wild (if they are), and that additionally you sampled 33 wild trees not viewed as ancient. Also, we still don’t know what you mean by “ancient”.
99: Some information needed here on what went into vitality assessment. The information in the supplement is good; abstract it to a few sentences for the main text.
99-100. is the low mean level of vitality in the ancient individuals just a consequence of the fact that they are old?
104-106. Here we learn that dbh is used as a proxy for age; this information should be indicated up front. It is not a response in your study, but a predictor (the only proxy I gather of ancientness).
106—109. This is a bit circular: DBH is your proxy for age, age is associated with vitality, vitality is associated with DBH. Probably only report on 2 of these, not 3 (as though we are getting three pieces of distinct information; the association betw dbh and age is assumed, and the association between vitality and dbh / age is observed.
138-140. The total number of ancient individuals in each province is less interesting than the percent of individuals sampled in each province that are ancient; and even that’s of questionable use, b/c we don’t know how you decided how many individuals to sample from each population.
147-148: “these patterns allowed us to 148 distinguish 26 natural relics from 38 historically introduced ancient trees” --- not clear how this was done. Even looking at Supplemental Fig. 7, I’m not sure how you inferred this.
177-179: this is highly circular: PSMC uses heterozygosity to infer demographic history, so the finding of a high correlation between heterozygosity and depth ($r2 = 0.975$) is exactly what is expected.
186—188: higher Ne is an expected outcome of admixture; so I’m not sure what this result is intended to tell us.
201—204. Admixed lineages will of course have higher heterozygosity, so this is not so much a finding as a report of the known relationship between heterozygosity and admixture; then there is only a ca. 2-fold difference in pi between Zhujiang and Minjiang, which I wouldn’t think qualifies as “substantial.”
301-305. I think this is the first time we get a clear idea that “genomic erosion” is being used to mean “erosion of genomic diversity.” If that’s the use throughout, make that clear up front.
311-316. As discussed above, I don’t find this “lineage” effect persuasive. The admixed individuals are expected to have high diversity, so that’s not much of a finding; and the difference between the unadmixed lineages seems pretty minimal.
Figs 4h, i --- these are hard to interpret but very important.

Reviewer #3 (Comments for the Author):

This is an interesting study that tests the long-standing view that old trees are more genetically diverse and unique. The authors compare genomic data from 64 ancient, 33 wild, and 50 cultivated individuals of Glyptostrobus pensilis and find that population lineage identity, rather than individual age, contributes most to genome-wide diversity. This conclusion challenges the conventional assumption that long-lived individuals are inherently genetically irreplaceable and has potential implications for the conservation of this endangered species. The study is comprehensive, with relatively large-scale sampling and analyses from multiple perspectives.

I am not a specialist in genomic diversity, but I am familiar with dendrochronology and ancient trees. So, I only have several concerns regarding the framing and methodology, which may affect the strength of the conclusions.

Major comments
1. Overstatement of novelty

The manuscript states that “a central assumption… remains surprisingly untested,” which appears overstated. Several previous studies have addressed related questions, including:
1. Mosseler, A., Major, J. E., & Rajora, O. P. (2003). Old-growth red spruce forests as reservoirs of genetic diversity and reproductive fitness. Theoretical and Applied Genetics, 106(5), 931-937.
2. Hanlon, V. C., Otto, S. P., & Aitken, S. N. (2019). Somatic mutations substantially increase the per-generation mutation rate in the conifer Picea sitchensis. Evolution Letters, 3(4), 348-358.
3. Díez, C. M., Trujillo, I., Barrio, E., Belaj, A., Barranco, D., & Rallo, L. (2011). Centennial olive trees as a reservoir of genetic diversity. Annals of Botany, 108(5), 797-807.

These studies, although using different approaches, generally support the idea that old trees can act as reservoirs of genetic diversity, which contrasts with the conclusions of the present manuscript. While the genomic methods used here are more advanced, the authors should better acknowledge this body of work and more carefully position their contribution relative to existing studies.

2. Lack of direct age estimation
The ages of the 147 sampled trees are not directly quantified. Instead, diameter at breast height (DBH) is used as a proxy to distinguish ancient and wild trees. Although Tang et al. (ref. 18) reported a positive relationship between DBH and age, this relationship is based on a case study from Fujian Province and may not be broadly applicable. Given that G. pensilis is a relatively fast-growing species, large individuals in other regions may still be relatively young. As a result, conclusions related to individual age are not well supported. More importantly, the study conflates conservation categories (ancient, wild, cultivated) with tree age. To directly test the central hypothesis, it would be preferable to incorporate dendrochronological data or other reliable age estimates and explicitly analyses the relationship between individual age and genomic diversity.

3. Interpretation of population history versus age effects
I agree that population history is likely a major driver of genomic diversity. However, this conclusion would be more convincing if the authors compared individuals with similar population histories but different ages within the same region. Such a design would allow a more direct test of the relative importance of age versus lineage.

4. Limitations of species choice and sample size
The relatively small sample size may limit the robustness of the conclusions. In addition, G. pensilis rarely exceeds ~150 years (ref. 18), meaning that the sampled “old” trees may not represent truly long-lived individuals. Extremely old trees (e.g., >500 years) are often survivors from historically large and genetically diverse populations and may represent “lottery winners” (ref 2). In contrast, G. pensilis populations have been strongly impacted by human activities and may largely consist of younger cohorts. Therefore, the observed patterns could be strongly influenced by population history and genetic drift, rather than providing a definitive test of whether very old trees harbor unique genetic variation.

Given these limitations, the conservation implications should be framed more cautiously.

Minor comments
Lines 91–112: The section describing poorer vitality in ancient trees is not directly relevant to the main research question and could be substantially shortened.
Lines 370–371: The information presented appears to be based on anecdotal sources and may not be reliable; it should be revised or removed.

********************END********************

Version 1:

Reviewer comments:

Reviewer #1

(Comments for the Author)
I think that the authors have done a very thorough job in revising the manuscript in line with my previous comments and those of the other authors. I've thought about this paper a lot in the time since submitting my original review and think that it makes a great contribution to the field.

I have no additional feedback of substance.

Another point that I've thought about is that old, large trees, probably have a high reproductive output (both in pollen and seeds) relative to juveniles, and have the effect of reducing the effective population size in the local area. I only mention this to indicate to the authors that their paper has made me think a lot, so thanks!

Reviewer #3

(Comments for the Author)
After reviewing the comments from the other reviewers, I noted that several concerns were shared across the reviews, particularly with respect to the methodology, the choice of study species, and the conclusions drawn from the analyses. In my view, the authors have made a substantial effort to address these concerns during the revision process. They have also moderated and clarified their main conclusions accordingly. I am satisfied with the revisions and have no further comments.

Decision Letter:

Our ref: NPLANTS-260222391A

9th June 2026

Dear Dr. Kang,

Thank you for submitting your revised manuscript "Population history rather than tree age contributes to the evolutionary importance of ancient trees in an endangered conifer" (NPLANTS-260222391A). It has now been seen by the original referees and their comments are below. The reviewers find that the paper has improved in revision, and therefore we'll be happy in principle to publish it in Nature Plants, pending minor revisions to satisfy the referees' final requests and to comply with our editorial and formatting guidelines.

If the current version of your manuscript is in a PDF format, please email us a copy of the file in an editable format (Microsoft Word or LaTex)-- we can not proceed with PDFs at this stage.

We are now performing detailed checks on your paper and will send you a checklist detailing our editorial and formatting requirements in about a week. Please do not upload the final materials and make any revisions until you receive this additional information from us.

Thank you again for your interest in Nature Plants Please do not hesitate to contact me if you have any questions.

Sincerely,

Reviewer #1 (Comments for the Author):

I think that the authors have done a very thorough job in revising the manuscript in line with my previous comments and those of the other authors. I've thought about this paper a lot in the time since submitting my original review and think that it makes a great contribution to the field.

I have no additional feedback of substance.

Another point that I've thought about is that old, large trees, probably have a high reproductive output (both in pollen and seeds) relative to juveniles, and have the effect of reducing the effective population size in the local area. I only mention this to indicate to the authors that their paper has made me think a lot, so thanks!

Reviewer #3 (Comments for the Author):

After reviewing the comments from the other reviewers, I noted that several concerns were shared across the reviews, particularly with respect to the methodology, the choice of study species, and the conclusions drawn from the analyses. In my view, the authors have made a substantial effort to address these concerns during the revision process. They have also moderated and clarified their main conclusions accordingly. I am satisfied with the revisions and have no further comments.

Version 2:

Decision Letter:

9th July 2026

Dear Professor Kang,

We are pleased to inform you that your Article entitled "Population history rather than tree age contributes to the evolutionary importance of ancient trees in an endangered conifer", has now been accepted for publication in Nature Plants.

Over the next few weeks, your paper will be copyedited to ensure that it conforms to Nature Plants style. We look particularly carefully at the titles of all papers to ensure that they are relatively brief and understandable.

Once your paper is typeset, you will receive an email with a link to choose the appropriate publishing options for your paper and our Author Services team will be in touch regarding any additional information that may be required.

After the grant of rights is completed, you will receive a link to your electronic proof via email with a request to make any corrections within 48 hours. If, when you receive your proof, you cannot meet this deadline, please inform us at rjsproduction@springernature.com immediately.

You will not receive your proofs until the publishing agreement has been received through our system.

You may wish to make your media relations office aware of your accepted publication, in case they consider it appropriate to organize some internal or external publicity. Once your paper has been scheduled you will receive an email confirming the publication details. This is normally 3-4 working days in advance of publication. If you need additional notice of the date and time of publication, please let the production team know when you receive the proof of your article to ensure there is sufficient time to coordinate. Further information on our embargo policies can be found here:
https://www.nature.com/authors/policies/embargo.html

Due to the importance of these deadlines, we ask you please us know now whether you will be difficult to contact over the next month. If this is the case, we ask you provide us with the contact information (email, phone and fax) of someone who will be able to check the proofs on your behalf, and who will be available to address any last-minute problems.

Acceptance of your manuscript is conditional on all authors' agreement with our publication policies (see http://www.nature.com/authors/policies/index.html). In particular your manuscript must not be published elsewhere.

If you have any questions about our publishing options, costs, Open Access requirements, or our legal forms, please contact ASJournals@springernature.com

Authors may need to take specific actions to achieve compliance with funder and institutional open access mandates. If your research is supported by a funder that requires immediate open access (e.g. according to Plan S principles or the NIH public access policy) then you should select the gold OA route, and we will direct you to the compliant route where possible. Because authors warrant under our subscription licensing terms that they haven’t committed to licensing any version of their article under a licence inconsistent with the terms of our agreement – including the applicable embargo period – publication under the subscription model isn’t suitable for authors whose funders require no embargo.

An online order form for reprints of your paper is available at https://www.nature.com/reprints/author-reprints.html. All co-authors, authors' institutions and authors' funding agencies can order reprints using the form appropriate to their geographical region.

You mentioned about the submission of potential cover material (including a short caption of around 40 words) related to your manuscript; suggestions should be sent to Nature Plants as electronic files (the image should be 300 dpi at 210 x 297 mm in either TIFF or JPEG format), preferably during your copyediting period. Please note that such pictures should be selected more for their aesthetic appeal than for their scientific content, and that colour images work better than black and white or grayscale images. Please include a written description of your image and how it was created along with your image files. Please do not try to design a cover with the Nature Plants logo etc., and please do not submit composites of images related to your work. I am sure you will understand that we cannot make any promise as to whether any of your suggestions might be selected for the cover of the journal.

You can now use a single sign-on for all your accounts, view the status of all your manuscript submissions and reviews, access usage statistics for your published articles and download a record of your refereeing activity for the Nature journals.

To assist our authors in disseminating their research to the broader community, our SharedIt initiative provides you with a unique shareable link that will allow anyone (with or without a subscription) to read the published article. Recipients of the link with a subscription will also be able to download and print the PDF.

As soon as your article is published, you will receive an automated email with your shareable link.

With kind regards,

P.S. Click on the following link if you would like to recommend Nature Plants to your librarian
http://www.nature.com/subscriptions/recommend.html#forms

** Visit the Springer Nature Editorial and Publishing website at www.springernature.com/editorial-and-publishing-jobs for more information about our career opportunities. If you have any questions please click here.**

Open Access This Peer Review File is licensed under a Creative Commons Attribution 4.0 International License, which permits use, sharing, adaptation, distribution and reproduction in any medium or format, as long as you give appropriate credit to the original author(s) and the source, provide a link to the Creative Commons license, and indicate if changes were made. In cases where reviewers are anonymous, credit should be given to 'Anonymous Referee' and the source.
The images or other third party material in this Peer Review File are included in the article's Creative Commons license, unless indicated otherwise in a credit line to the material. If material is not included in the article's Creative Commons license and your intended use is not permitted by statutory regulation or exceeds the permitted use, you will need to obtain permission directly from the copyright holder.
To view a copy of this license, visit https://creativecommons.org/licenses/by/4.0/

Reviewers' Comments:

Reviewer #1 (Comments for the Author):

Review of Zhang et al - submitted to Nature Plants

I think that this is a very nice paper and one that will fit nicely in Nature Plants. I learned a lot from reading this paper. Overall, I can't say that I'm overly surprised by the result that the ancient trees have similar genetic variation to the young trees, but that's not a knock against the paper. On the contrary, I agree with the authors that there is a general expectation that ancient trees would be reservoirs for genetic diversity, so this paper is tackling an open question. The data looks excellent and analyses are done well. This work represents a really nice example of conservation genomics, the authors integrate much of the recent literature in population genomics in their approach.

Answer: We sincerely thank the reviewer for this careful, constructive, and encouraging assessment of our manuscript, and greatly appreciate the recognition of the significance of the study, the quality of the data, and the strength of the analyses. We are particularly grateful that the reviewer acknowledges the value of our central result and views the study as a valuable contribution to conservation genomics.

The biggest issue I have with the paper is whether this species is actually an ideal test case for the question of whether old individuals possess beneficial alleles. It is argued in the Abstract (31-34), Introduction (Lines 69-70) and extensively in the Discussion (but predominantly on lines 346-347) that this notion can be rejected. However, after reading the paper I'm not sure that I agree.

There are numerous issues that are raised in the Discussion about the potential relaxation of selection in the ancient trees that they studied. A large fraction of the ancient trees analysed in this study have been cared for by humans, so it seems plausible that natural selection may have been relaxed for those individuals. In addition, the extremely limited population sizes in the species, which seem to have been very low for at least the last 5000 years would have led to high levels of drift. This history of high rates of drift may mean that the populations are not particularly well adapted. This is more or less supported by the pi0/pi4 results that the authors note as being at the upper end of what has been reported for plants.

The idea that the authors are arguing against is that old trees are reservoirs of beneficial alleles as they have withstood the test of time. However, if a population is not well adapted in the first place, then there would likely not be much of a difference between ancient and young trees. Furthermore, if the ancient trees are alive because humans have kept them alive, then they are not exemplars of the idea that ancient/old growth trees are reservoirs of beneficial alleles. I don't think that this undercuts the high quality of the paper, but I do think that this framing of the paper should be tempered. If you looked at, say, Norway Spruce or Douglas-fir, species with much larger population sizes, would you expect the same result? I'm not convinced that the results would be

that different, but without testing it we cannot be sure. This comment could be addressed by some re-wording and re-framing.

Answer: Thank you for this thoughtful and important comment. We agree that *Glyptostrobus pensilis* may not be an ideal or universally representative model for all long-lived tree species, and that its strong demographic contraction, drift, and frequent occurrence of ancient trees in human-dominated settings need to be considered carefully when drawing broader inferences from our results.

At the same time, we do not think that these features weaken our main result. Instead, they help explain why ancient-tree status alone may be an unreliable guide to genomic importance. As the reviewer notes, if some ancient trees have persisted partly because they were protected by humans, or if long-term small population size and drift have reduced adaptive efficiency, then old age by itself cannot be taken as evidence that those individuals are genetically exceptional survivors. In this sense, *G. pensilis* provides a realistic and conservation-relevant case in which to evaluate whether ancient-tree status alone is informative, or whether lineage history provide a better explanation. Consistent with this interpretation, despite broad differences in age structure among ancient, wild, and cultivated trees, genomic contrasts are clearer among the ancestral–admixed, Zhujiang, and Minjiang backgrounds than among the three conservation categories themselves.

We also think that related logic may extend beyond highly endangered species, although its generality across other systems will require direct testing. Even in more widespread taxa that may better exemplify the idea that ancient or old-growth trees can act as reservoirs of beneficial alleles, larger effective population sizes would not necessarily erase lineage structure. Where regeneration and gene flow are more extensive, variation carried by old trees may also be more readily transmitted to and retained in extant populations. Under such conditions, the evolutionary importance of old individuals may still depend less on age itself than on lineage history and on whether they retain genomic variation that remains underrepresented in extant populations.

Accordingly, we have tempered the framing throughout the revised manuscript, including the Abstract (Lines 19, 32–34), Introduction (Lines 95–97), and Discussion (Lines 367–371 and 390–398). We now make clearer that our main conclusion is specific to this study system: in *G. pensilis*, ancient-tree status did not consistently predict greater overall genomic variation, and much of the observed heterogeneity was better explained by lineage history than by age alone, while also discussing the possibility that similar logic may extend to other systems.

I have some other questions about the paper, some big, some small.

Major comments:

What is an ancient tree? It is not described in the Intro or the results what actually constitutes an ancient tree. This is given in the methods (age more than 100 years), but for the sake of readability I think that this should be mentioned somewhere near the beginning. I couldn't find a table or anything with age estimates for all the samples. It seems that there may be a considerable difference between a tree that is 2100 years old and one that is 100 years old. For the results on vitality, a little more justification would be good. It seems strange to me to incorporate anthropogenic disturbance into a vitality metric. Such a disturbance doesn't seem like it is really in the spirit of what the authors are trying to measure - whether ancient trees are specifically well adapted.

Answer: Thank you for these important and helpful comments. We have addressed this set of concerns by clarifying the definition of ancient trees earlier in the manuscript, providing age-related information more explicitly, and revising the description and interpretation of vitality/current condition.

(1) Definition of ancient trees. We now define ancient trees much earlier in the manuscript. Specifically, in the Abstract (Lines 22–23), Introduction (Lines 86–89), Results (Lines 110–112), and Methods (Lines 407–415), we state that, in this study, ancient trees refer to documented individuals older than 100 years occurring in human-dominated landscapes, following Huang et al. (2023, *Nat. Plants*), while excluding individuals persisting in remnant natural swamp habitats. This definition is also practical in our study system, because such trees typically receive long-term local attention and are therefore more likely to have documentary age-related records. We also clarify explicitly that ancient, wild, and cultivated trees are treated as three non-overlapping conservation types.

(2) Age information and the range of ages among ancient trees. We now provide the recorded ages of all sampled ancient trees in Results (Lines 108–117), Supplementary Table 3, and add a fuller explanation of the age sources in Methods (Lines 421–439). All sampled ancient trees are supported by available records as being older than 100 years, and most recorded ages fall between 150 and 800 years after excluding the two lowest and two highest recorded values. We also summarize the available age-related information for the wild and cultivated types in the main text. However, we did not further subdivide ancient trees into narrower age classes in our analyses because the available age records derive from heterogeneous sources, including published studies, forestry records, archival documentation, news reports, and local interviews, and therefore do not provide equally precise estimates for all individuals. In addition, the Critically Endangered status of *G. pensilis* and the associated protection constraints make destructive or invasive direct age estimation unrealistic in our study system. We therefore use the age records to define ancient-tree status, but avoid treating recorded age as a precise continuous variable in the genomic analyses. We have clarified this point in the Methods (Lines 430–433).

(3) Vitality/current-condition assessment. We have revised this part of the manuscript to better reflect our intended interpretation. Our purpose was not to test whether ancient trees are intrinsically better or less well adapted, but to describe their present field condition and the associated conservation urgency. In the revised manuscript, we summarized the assessment criteria more clearly in the Results (Lines 122–129), with further details provided in the Methods (Lines 443–455) and Supplementary Note 1. We have also removed anthropogenic disturbance as a direct component of the vitality/current-condition score, because we agree that it is better treated as a potential contributing factor to poor condition rather than as part of the condition metric itself. The revised wording therefore focuses on crown architecture, structural integrity, growth condition, reproductive status, and habitat quality.

What fraction of the ancient trees were maintained as fengshui trees (or maintained by humans in other ways)? Basically my question is what is the expected lifespan of this species in the absence of human intervention? Most of the wild population were classified as healthy, but these are not managed. Maybe it's just the case that typical causes of mortality have been eliminated for the ancient trees. I appreciate that that is largely speculative, but this does bear on the overall thrust of the paper. The main message is that ancient trees don't harbour important genetic variation. But if the ancient trees are only ancient because humans have prevented them from dying, maybe this species is not the best test of this theory. What is the distribution of DBH and vitality for the natural relics versus the historically cultivated ancients?

Answer: We agree that human influence may have contributed to the persistence of at least some ancient *G. pensilis* trees in human-dominated landscapes. Among the 64 sampled ancient trees, 24 have recorded ages exceeding 500 years (Fig. 1c and Supplementary Table 3), whereas the oldest currently reported wild *G. pensilis* individual is ~357 years old (Tang et al. 2019, *Plant Divers.*). This pattern is consistent with the possibility that human association has helped at least some individuals persist to greater ages. However, it does not necessarily mean that typical causes of mortality have simply been eliminated for ancient trees. Our field surveys also documented substantial anthropogenic disturbance, habitat degradation, and other external stresses affecting many ancient individuals (Extended Data Fig. 1b). Nor can we exclude some degree of survivor bias, especially given that only about 100 ancient trees remain. In light of the endangered status of this species, its genomic erosion, and the potential role of human influence in this study system, we have adjusted the manuscript accordingly and no longer present *G. pensilis* as a definitive general test of whether very old trees harbor unique genetic variation across all long-lived tree systems. The corresponding framing changes are reflected in the Abstract (Lines 32–34), Introduction (Lines 95–97), and Discussion (Lines 367–371 and 390–395).

More importantly, our study system also highlights a more realistic conservation scenario. Nearly all surviving ancient trees in this species now receive some degree of human attention, management, or repair, so we could not reliably estimate a meaningful fraction maintained specifically as fengshui trees or under other distinct categories of

care. For this reason, rather than classifying ancient trees by type of human maintenance, we used genetic evidence together with geographic context and affinity to nearby wild populations to distinguish likely natural relics from historically introduced individuals (Results, Lines 159–168; Methods, Lines 550–558; Fig. 2a; Supplementary Table 3). This provenance-based distinction is central to our interpretation. Many genetically redundant ancient trees are concentrated in the historically introduced subset and largely belong to the Minjiang lineage, whereas some likely natural-relic ancient trees from the Zhujiang lineage and the ancestral–admixed group retain private variation that remains poorly represented in extant populations. Thus, the potential role of human protection does not invalidate the study; instead, it reinforces the need to distinguish tree age itself from the lineage history, provenance, and genetic representation that shape the evolutionary importance of ancient trees.

With respect to the comparison between likely natural-relic ancient trees and historically cultivated ancient trees, our results do not show a simple contrast in present condition (Supplementary Table 1). Among the 26 likely natural-relic ancient trees, 3 were classified as Dying, 13 as Poor, 9 as Fair, and 1 as Moderate. Among the 38 historically cultivated ancient trees, 16 were classified as Dying, 7 as Poor, 10 as Fair, and 5 as Moderate. Thus, the proportion of trees in Poor or Dying condition was broadly similar between the two groups (16/26 = 61.5% in likely natural relics; 23/38 = 60.5% in historically cultivated ancient trees), although the distribution among condition categories differed. The compiled age-related records, with DBH now treated only as supporting evidence rather than as a proxy for age, indicate that the 38 historically cultivated ancient trees have a somewhat higher mean recorded age than the 26 likely natural-relic ancient trees (455 versus 388 years), whereas the likely natural-relic group shows greater variance and includes the two most extreme recorded ages (1,209 and 2,113 years). As our focus is primarily on their genetic differences, and the age- and condition-related differences between them are limited, we do not emphasize these comparisons further in the main text.

Supp Fig S8 - On the PCA - PC1 was dominated by the placement of two of the admixed individuals. Were those individuals different in any other ways? Are they the two individuals that form the outermost clade in Figure 2d?

Answer: We re-checked the sample identities and confirmed that these are the same two admixed individuals shown at the extreme end of PC1 in the PCA (Supplementary Fig. 8) and that they form the two outermost ingroup tips in the SVDquartets tree (Fig. 2d).

To make this clearer, we have added the following sentence to the revised legend of Supplementary Fig. 8: “The five dispersed points in the PCA correspond to the admixed individuals described in the main text. Among them, the two admixed individuals (FN1 and TD1) at the extreme end of PC1 are the same two samples that form the two outermost ingroup tips in the SVDquartets tree shown in Fig. 2d of the main text.”

PSMC - what are we to infer from the finding that the maximum depth differs among the different groups? What is the relevance of this depth and it's correlation with heterozygosity. Of course the higher the heterozygosity the longer to coalescence (i.e. neutral coalescence time is expected to be 2Ne and nucleotide diversity/heterozygosity is expected to be 4Neu). If it's just that, I don't think that it needs to be presented alongside the major results. If it's not, the authors should give some description about what they want us to take away from that.

Answer: We agree. We therefore removed the heterozygosity–depth correlation from the main text and moved the corresponding panel to the Supplementary Fig. 9, where it is retained only as supporting context for understanding differences in the depth range over which PSMC profiles can be recovered. The revised Results now focus on the lineage-specific demographic trajectories themselves.

Additionally, I generally think that relating PSMC results to geological time can be a bit tricky given how assumption laden the estimate of generation time and mutation rate is. It would be good to have that uncertainty reflected in the description of how the PSMC results relate to the geological record.

Answer: We agree that uncertainty associated with mutation-rate and generation-time assumptions should be reflected more clearly when relating PSMC trajectories to the geological events. In the revised manuscript, we retained the broad comparison with Quaternary sea-level history, while adding wording to indicate that the temporal correspondence is approximate rather than exact.

The revised text now reads (Lines 200–206): "All groups showed temporal patterns broadly coincident with Quaternary sea-level fluctuations under the assumed mutation-rate and generation-time parameters, including an early bottleneck and partial recovery during long-term sea-level decline (~5–1 Ma), followed by subsequent demographic shifts associated with intermediate sea-level oscillations (Fig. 3a). Effective population sizes (N_e) fell to ~400–1,000, approximately corresponding to the period approaching the Last Glacial Maximum (LGM)."

Private alleles - one of the signature that we might expect for the ancient trees is that they possess genetic variation that is not present in the younger trees. Aside from the admixed individuals, Figure 2e shows that the ancient trees don't seem to have any greater proportion of private alleles than the young ones. That seems worth a mention in the text. I appreciate that this is returned to in the section beginning on Lines 251.

Answer: We have added a brief statement to the Results (Lines 192–194) to make this point explicit: "Aside from these admixed individuals, ancient and non-ancient wild individuals in the Zhujiang and Minjiang lineages showed broadly similar private-allele proportions." The more detailed analysis of rare-allele representation across ancient and non-ancient wild and cultivated individuals remains in the later Results section.

Mutation rate - the mutation rate that is stated in the methods is rather low (line536). The authors also used different estimates for *fastsimcoal* and PSMC. Did I miss something in why these are so different? As a side note, Hanlon et al estimate somatic mutation rates in sitka spuce and got an estimate of ~2.7e-8/bp/generation (which was higher than angiosperms) - that may provide a more empirical justification for the mutation rate choice than the citation given, which was based on divergence data.

Answer: We apologize for the confusing presentation in the original Methods. We did not use different mutation rates for PSMC and fastsimcoal2. In the original version, the apparent discrepancy arose from reporting format, not from different assumptions.
Both PSMC and fastsimcoal2 used the same Cupressaceae-based annual mutation rate (5.92216×10^{-10} site^{-1} year^{-1}) and the same generation time (50 years), as estimated by De La Torre et al. (2017, Mol. Biol. Evol.). The PSMC section reported the rate on a per-year basis, whereas the fastsimcoal2 section reported the equivalent per-generation value (2.96×10^{-8} site^{-1} generation^{-1}). We have revised the Methods (Lines 605–607 and 635–636) accordingly so that the mutation rate is now reported consistently on a per-generation basis throughout the manuscript.

We also thank the reviewer for suggesting Hanlon et al. (2019, *Evol. Lett.*) as an empirical reference for conifers. The per-generation estimate reported there for Sitka spruce (*Picea sitchensis*; Pinaceae), approximately 2.7×10^{-8} bp^{-1} generation^{-1}, is very similar in magnitude to the per-generation mutation rate assumed in our study (2.96×10^{-8} site^{-1} generation^{-1}). However, because our study species, *Glyptostrobus pensilis*, belongs to Cupressaceae, we conservatively retained the Cupressaceae-based estimate from De La Torre et al. (2017, *Mol. Biol. Evol.*) in our analyses. Given the small difference between the two estimates, this choice is unlike to affect the main conclusions.

Nucleotide diversity - no statement of uncertainty is given in the text, or in Figure 4a. Perhaps bootstrap samples of the 200kbp windows could be done to obtain empirical estimates of error? I don't think that this will change the conclusions from lines 199-206, but would probably strengthen the authors argument that there is little difference between the groups.

Answer: We thank the reviewer for this helpful suggestion. We estimated uncertainty for genome-wide nucleotide diversity by bootstrap resampling of the 200-kb windows within each group (1,000 replicates) and used the resulting bootstrap distribution to derive 95% confidence intervals. These revisions are now reflected in Fig. 4a and Methods (Lines 649–651). The resulting confidence intervals are consistent with our original interpretation that differences among conservation groups are comparatively small.

Landscape of nucleotide diversity - for people like me who find it useful to look at these graphs, it would be nice to include versions of 4e for the other 10 chromosomes. Throwing these into the supplement would be sufficient.

Answer: We have now added nucleotide-diversity landscapes for the remaining 10 chromosomes (Chr2–Chr11) to the Supplementary Fig. 12.

Minor points

Lines 37-47 - another aspect of ancient trees is that their typically large size supports a diverse ecosystem of micro and macro organisms. There is also the notion that ancient trees may foster mycorrhizal connections that support forest ecosystems in other ways. I leave it to the authors to take from this what they want, but suggest that a little more on the potential importance of ancient trees beyond their genetic diversity would be a good addition to the introduction.

Answer: We agree and have revised the Introduction accordingly. We added a brief statement noting that ancient trees can also function as structural anchors that support local biodiversity through the provision of persistent habitat and microhabitats for diverse organisms (Lines 41–43).

Line 423 - How does Illumina read mapping demonstrate assembly quality in this context?

Answer: We agree and have revised the text (Lines 139–142) to clarify that the high Illumina read-mapping rate is presented as one of several assembly-quality metrics, alongside BUSCO completeness and Long Terminal Repeat Assembly Index (LAI).

Figure 2e - is the colour code the same as in the previous panels?

Answer: Yes. The colour code is consistent across Fig. 2b–e, and we have clarified this in the revised figure legend (Lines 1045–1046).

Reviewer #2 (Comments for the Author):

The authors address a very important question: do ancient trees retain higher genetic diversity or higher amounts of rare alleles, supporting the argument that they are disproportionately important to population survival. I applaud the authors for taking on this question.

Answer: We sincerely thank the reviewer for recognizing the importance of this question and for the positive assessment of the study's motivation. We have carefully revised the manuscript to clarify the interpretation of our results.

However, the study never makes clear how "ancient" individuals are defined, except by DBH. Yet (1) we never are shown any data suggesting a tight correlation between DBH and age, a correlation that can be quite loose because of plastic growth rates; and (2) the DBH distribution overlaps between the ancient and non-ancient individuals (Fig 1d), demonstrating that some other criterion is used to identify ancient individuals. I did not see any description of how this categorization was made, leaving me to wonder whether ancient trees were defined based on tradition or subjective criteria. In light of this, I don't know how to interpret the central claims of the manuscript.

Answer: Thank you for this important comment. We agree that, in the original manuscript, the basis for classifying "ancient" individuals was not stated clearly enough early in the text, which could make our central comparisons difficult to interpret. We have substantially revised the manuscript to address this issue.

First, ancient trees in this study are defined as individuals older than 100 years occurring in human-dominated landscapes (following Huang et al. 2023, *Nat. Plants*), while excluding individuals persisting in remnant natural swamp habitats. Accordingly, ancient, wild, and recently cultivated (~20–50 years old) trees are treated as three separate, non-overlapping conservation types. These categories are defined independently based on ecological context, management history, site characteristics, and age-related documentation. We now state this definition explicitly at an earlier stage in the manuscript, specifically in the Abstract (Lines 22–23), Introduction (Lines 86–89), Results (Lines 110–112), and Methods (Lines 407–415).

Second, we agree that DBH alone should not be used as the sole basis for assigning ancient-tree status, especially given plasticity in growth rates. To address this concern, we have expanded the age-related information for all three conservation types and now present DBH only as supporting size-related information. These age records were compiled from multiple sources, including published studies, forestry records, archival documentation, news reports, and local interviews, with source reliability evaluated conservatively. For ancient trees, all 64 sampled individuals are supported by available records as being older than 100 years, and most recorded ages fall between 150 and 800 years after excluding the two lowest and two highest recorded values. For the wild type, 22 of 33 sampled individuals came from Fujian and 3 from Vietnam, the two main sampled wild-population regions of *G. pensilis*, for which published mean ages are ~85

years (Tang et al. 2019, *Plant Divers.*) and ~112 years (Averyanov et al. 2009, *Taiwania*), respectively; the remaining 8 wild individuals came from Guangdong, where even the largest reported tree is unlikely to exceed ~70 years. Cultivated trees were mostly recent plantings (~20–50 years old), based on planting histories, local records, and information from site managers. Thus, although overlap in DBH is expected, the available evidence still supports broad differences in age structure among the three conservation types. The corresponding additions and clarifications are now incorporated in the Results (Lines 108–121), Methods (Lines 421–442), and Supplementary Table 3.

With these revisions, we believe these changes now make the basis for defining ancient trees much clearer and allow the genomic comparisons among ancient, wild, and cultivated individuals to be interpreted more appropriately.

Additionally, I have some methodological concerns and specific points that bear addressing:

22: define "ancient" on first use (even in abstract, in my opinion); also, are the "ancient" trees spread across both cultivated and wild? Surely "ancient" is not a category distinct from "wild" and "cultivated"

Answer: We agree that the definition of "ancient" should be made explicit at first use and that the relationship among the three sampled conservation types required clarification. In the revised manuscript, we now state this explicitly in the Abstract (Lines 22–23), Introduction (Lines 86–89), Results (Lines 110–112), and Methods (Lines 407–415). These revisions make clear that, in our sampling design, ancient, wild, and cultivated are treated as three separate, non-overlapping conservation types.

55: I'm not sure what concept you have in mind by "genetically eroded survivors". I think you are talking about individual trees; do you just mean maladapted? Or low-heterozygosity? I assume you mean one or both of these, but in either case "genetically eroded" doesn't clearly convey to me what you might have in mind.

Answer: Thank you for pointing this out. We agree that the phrase "genetically eroded survivors" did not clearly convey our intended meaning in the original version. Our intention was not to imply that individual ancient trees are necessarily maladapted, but rather to refer to individuals or lineages that have persisted despite reduced genomic diversity, increased inbreeding, and elevated genetic load resulting from long-term demographic decline. To clarify this concept, we have revised the Introduction to define genomic erosion more explicitly.

The revised text now states (Lines 50–53): "Range contraction, habitat fragmentation and poor natural regeneration typically reduce genomic diversity while increasing inbreeding and genetic load, collectively resulting in genomic erosion with potentially negative consequences for long-term viability." This change also makes the related wording later in the manuscript (Line 57–59 and 330–331) easier to interpret.

66-68: It is true that "size-based protection can fail to safeguard genuinely old individuals", but it does not follow that "a direct genomic test is needed to evaluate whether ancient trees retain irreplaceable genetic variation." These are two separate ideas that should be separated into separate sentences.

Answer: We have separated these two points in the revised Introduction. The text now reads: "In addition, size-based protection can fail to safeguard genuinely old individuals[13]. Whether ancient trees retain irreplaceable genetic variation therefore cannot be inferred from age, size, or status alone, and is best evaluated through genomic tests." (Lines 68–71)

73: Delete "Once"

Answer: Done. "Once" has been removed, and the sentence has been revised accordingly (Lines 76–79).

85: "more than 100 ancient, wild and cultivated individuals" – this doesn't align with the number of individuals you provide in the abstract or the results

Answer: We have revised the Introduction (Line 92) to state the exact sample size: "147 individuals spanning ancient, wild and cultivated trees."

95-96: as in the abstract, you need to make clear that the ancient trees are all wild (if they are), and that additionally you sampled 33 wild trees not viewed as ancient. Also, we still don't know what you mean by "ancient".

Answer: As clarified above, "ancient," "wild," and "cultivated" are treated here as three non-overlapping conservation types. We now define "ancient" explicitly at first use in the Abstract (Lines 22–23), and Introduction (Lines 86–89), Results (Lines 110–112), and retain the fuller description in the Methods (Lines 407–415). We also state that the 33 wild trees are separate, non-ancient individuals from remnant natural swamp habitats.

99: Some information needed here on what went into vitality assessment. The information in the supplement is good; abstract it to a few sentences for the main text.

Answer: Thank you for this helpful suggestion. We have therefore added a concise summary in the Results (Lines 122–125): "Ancient trees were more frequently in poor condition than wild and cultivated trees, highlighting greater conservation urgency, based on current-condition categories assigned from field observations of crown architecture, structural integrity, growth condition, reproductive status and habitat quality (see Methods and Supplementary Note 1)."

99-100. is the low mean level of vitality in the ancient individuals just a consequence of the fact that they are old?

Answer: We agree that the lower mean vitality of ancient individuals is likely related in part to their older age, which is often accompanied by severe crown loss or apical dieback, trunk damage or hollowing, and reduced growth and reproduction. However, age alone is unlikely to explain all of the observed variation in condition. Some ancient trees were also exposed to additional stresses, including degraded pneumatophores, poor habitat quality, heavy epiphytic loads, and pronounced anthropogenic or environmental disturbance (Extended Data Fig. 1b), which may further contribute to poor condition. Thus, the lower condition scores of ancient trees likely reflect both their old age and the stressful environments in which many surviving individuals persist. We have revised and condensed the main-text description accordingly (Lines 108–125), while retaining more detailed information on the current-condition assessment of the 147 sampled individuals in Supplementary Note 1.

104-106. Here we learn that dbh is used as a proxy for age; this information should be indicated up front. It is not a response in your study, but a predictor (the only proxy I gather of ancientness).

Answer: We thank the reviewer for this important clarification. We have clarified this point by presenting more explicit age-related information earlier in the Results (Lines 108–121) and by treating DBH only as supporting size-related information rather than as a primary proxy for age. The revised text about DBH now reads (Lines 118–121): "DBH differed in the same general direction among the three conservation types (median: 89.2 cm in ancient, 49.4 cm in wild and 31.1 cm in cultivated trees; Fig. 1d and Supplementary Table 1), providing supporting size-related evidence consistent with the age-related patterns summarized above."

106—109. This is a bit circular: DBH is your proxy for age, age is associated with vitality, vitality is associated with DBH. Probably only report on 2 of these, not 3 (as though we are getting three pieces of distinct information; the association betw dbh and age is assumed, and the association between vitality and dbh / age is observed.

Answer: We have removed the DBH–vitality analysis from the main Results to avoid overinterpreting DBH. The revised text (Lines 108–129) now emphasizes only two distinct patterns: broad age-structure differences among the three conservation types, supported by available age-related information, and poorer field condition of ancient trees relative to wild and cultivated trees.

138-140. The total number of ancient individuals in each province is less interesting than the percent of individuals sampled in each province that are ancient; and even that's of questionable use, b/c we don't know how you decided how many individuals to sample from each population.

Answer: We have removed the provincial counts from the main text, and proportions would likewise be difficult to interpret because our ancient-tree sampling was not designed as a proportional survey across populations or provinces. As clarified earlier in the Results (Lines 105–107) and detailed in the Methods (Lines 415–418), most known surviving ancient trees were included where sampling was feasible.

147-148: "these patterns allowed us to 148 distinguish 26 natural relics from 38 historically introduced ancient trees" --- not clear how this was done. Even looking at Supplemental Fig. 7, I'm not sure how you inferred this.

Answer: We now described explicitly how the 64 ancient trees were classified as likely natural relics or historically introduced individuals in the Methods (Lines 550–558): "To aid interpretation of ancient-tree provenance, we combined kinship structure with geographic context and genetic affinity to nearby wild populations. Ancient trees occurring as tight local clusters of individuals related within three degrees of kinship, especially when they also showed clear affinity to geographically nearby wild populations, were interpreted as likely historically introduced or locally propagated. By contrast, ancient trees showing little or no close kinship and lacking clear affinity to nearby extant wild populations were interpreted as likely natural relics, representing remnants of historically more extensive wild stands. On this basis, the 64 ancient trees were classified as individuals of likely historical introduction or likely natural-relic origin."

We also revised the corresponding sentence in the Results (Lines 159–168): "Eleven individuals from Fujian formed a 1st–3rd degree kinship cluster and were closely related to three nearby wild populations (Fig. 2a), suggesting historical introduction from local sources. Twenty-seven individuals from northeastern Jiangxi formed a single 1st-degree related cluster, most likely half-sibs, and were also closely related to the same nearby wild populations, consistent with a strong founder effect associated with human-mediated introduction. Together, these patterns identify 38 ancient trees as likely historically introduced. In contrast, the remaining 26 ancient trees showed little or no close kinship and lacked clear affinity to nearby extant wild populations (Fig. 2a and Supplementary Table 3), and are therefore inferred to represent likely natural relics.". We have also revised Supplementary Fig. 7 and added Supplementary Note 2 to provide a more detailed and explicit interpretation of kinship structure among ancient, wild and cultivated trees.

177-179: this is highly circular: PSMC uses heterozygosity to infer demographic history, so the finding of a high correlation between heterozygosity and depth (r2 = 0.975) is exactly what is expected.

Answer: We have removed the heterozygosity–depth correlation from the main text. The corresponding panel has been moved to Supplementary Fig. 9, where it is retained only as supporting context for interpreting why PSMC profiles differ in their recoverable depth among individuals. The revised Results now focus on the lineage-specific demographic trajectories themselves.

186—188: higher N_e is an expected outcome of admixture; so I'm not sure what this result is intended to tell us.

Answer: We agree that elevated inferred *N*e in the ancestral–admixed group may be expected under admixture. Our intention was not simply to emphasize that the ancestral-admixed group had higher N_e, but rather to highlight the broader temporal pattern in the PMSC trajectories. Specifically, although the ancestral-admixed group retained higher inferred N_e and showed a slower decline over earlier parts of the trajectory, its inferred N_e converged toward similarly low levels as the Zhujiang and Minjiang lineages in the more recent past. This pattern is consistent with a shared recent contraction in this endangered conifer.

To clarify this point and avoid overinterpreting the elevated N_e of the admixed group, we revised the text (Lines 206–211) as follows: "From ~1 Ma onward, lineage-specific trajectories emerged: both lineages underwent sustained declines, but Zhujiang showed a modest late-Pleistocene rebound, whereas Minjiang continued to contract without recovery. During the same interval, the ancestral–admixed group retained higher N_e and a slower decline, with N_e converging toward similarly low levels over the more recent part of the trajectory."

201—204. Admixed lineages will of course have higher heterozygosity, so this is not so much a finding as a report of the known relationship between heterozygosity and admixture; then there is only a ca. 2-fold difference in pi between Zhujiang and Minjiang, which I wouldn't think qualifies as "substantial."

Answer: We have revised the sentence in the Results (Lines 225–227) to present this pattern more cautiously: "In contrast, lineage-level differences were more pronounced: the ancestral–admixed group, as expected, showed the highest diversity ($\pi = 3.88 \times 10^{-3}$), Zhujiang was intermediate (1.42×10^{-3}), and Minjiang was lower (6.96×10^{-4})."

301-305. I think this is the first time we get a clear idea that "genomic erosion" is being used to mean "erosion of genomic diversity." If that's the use throughout, make that clear up front.

Answer: We agree that the intended meaning of "genomic erosion" should be defined clearly at first use. As described above, we now define this term earlier in the Introduction (Lines 50–53), so that its use here and elsewhere in the manuscript is clearer and more consistent.

311-316. As discussed above, I don't find this "lineage" effect persuasive. The admixed individuals are expected to have high diversity, so that's not much of a finding; and the difference between the unadmixed lineages seems pretty minimal.

Answer: We agree that the elevated diversity of the ancestral–admixed group is expected and, on its own, does not constitute a strong argument for a lineage effect. We have therefore revised this Discussion paragraph to reduce emphasis on the ancestral–admixed group and to focus instead on whether the two major lineages still show a consistent genomic contrast. In the revised text, our emphasis is not on any single metric alone, but on the fact that the two major lineages show a consistent genomic contrast across diversity-, inbreeding-, and load-related patterns, and that this contrast is more informative than the comparatively weak differences among ancient, wild, and cultivated trees despite their broad differences in age structure.

The revised text now reads (Lines 337–347): "Across the species, lineage history explains a larger part of genomic variation than conservation type that differ broadly in age structure. Even without considering the ancestral–admixed group, the two major lineages still showed a consistent genomic contrast: Zhujiang retained higher genetic diversity and lower inbreeding and genetic load, whereas Minjiang showed the opposite pattern, together with more extensive sharing of ROH islands. Notably, Zhujiang individuals combine relatively high overall diversity with elevated ROH, consistent with temporally lagged genomic signals in long-lived species[16]. By comparison, differences among ancient, wild, and cultivated trees were comparatively weak, both overall and within lineages, despite substantial heterogeneity among ancient trees."

Figs 4h, i --- these are hard to interpret but very important.

Answer: We have revised Figs. 4h and 4i by retaining only the homozygous ratios in the main figure and moving the heterozygous ratios to Supplementary Fig. 15. We hope this revision improves the presentation of the main figure while preserving the complete information, and we have updated the legend and Results text accordingly.

Reviewer #3 (Comments for the Author):

This is an interesting study that tests the long-standing view that old trees are more genetically diverse and unique. The authors compare genomic data from 64 ancient, 33 wild, and 50 cultivated individuals of Glyptostrobus pensilis and find that population lineage identity, rather than individual age, contributes most to genome-wide diversity. This conclusion challenges the conventional assumption that long-lived individuals are inherently genetically irreplaceable and has potential implications for the conservation of this endangered species. The study is comprehensive, with relatively large-scale sampling and analyses from multiple perspectives.

Answer: We sincerely thank the reviewer for this careful and encouraging assessment of our manuscript. We greatly appreciate the reviewer's recognition of the significance of the question addressed, the breadth of our sampling and analyses, and the potential conservation relevance of our main conclusion.

I am not a specialist in genomic diversity, but I am familiar with dendrochronology and ancient trees. So, I only have several concerns regarding the framing and methodology, which may affect the strength of the conclusions.

Major comments

1. Overstatement of novelty

The manuscript states that "a central assumption… remains surprisingly untested," which appears overstated. Several previous studies have addressed related questions, including:

1. Mosseler, A., Major, J. E., & Rajora, O. P. (2003). Old-growth red spruce forests as reservoirs of genetic diversity and reproductive fitness. Theoretical and Applied Genetics, 106(5), 931-937.
2. Hanlon, V. C., Otto, S. P., & Aitken, S. N. (2019). Somatic mutations substantially increase the per-generation mutation rate in the conifer Picea sitchensis. Evolution Letters, 3(4), 348-358.
3. Díez, C. M., Trujillo, I., Barrio, E., Belaj, A., Barranco, D., & Rallo, L. (2011). Centennial olive trees as a reservoir of genetic diversity. Annals of Botany, 108(5), 797-807.

These studies, although using different approaches, generally support the idea that old trees can act as reservoirs of genetic diversity, which contrasts with the conclusions of the present manuscript. While the genomic methods used here are more advanced, the authors should better acknowledge this body of work and more carefully position their contribution relative to existing studies.

Answer: Thank you for this constructive comment. We agree that our original wording overstated the novelty of the question. Our intention was not to imply that the possible genetic importance of old trees had not been considered previously. Rather, our starting point was the broader idea, supported by earlier studies, that old trees or old-growth

systems may act as reservoirs of genetic diversity. Building on that background, our study asks a more specific question: whether old individuals themselves can be treated as intrinsically valuable genetic reservoirs.

In response, we have revised the Introduction to acknowledge the relevant prior literature more explicitly and to position our contribution more carefully. Specifically, in the revised Introduction (Lines 46–49), we now state: "In this context, previous studies have suggested that ancient trees or old-growth systems may retain important genetic diversity[11],[12]. Whether this also means that ancient trees are themselves intrinsically valuable genetic reservoirs, however, remains insufficiently resolved." We now cite the studies by Mosseler et al. (2003, *Theor. Appl. Genet.*) and Díez et al. (2011, *Ann. Bot.*) in this context, as representative examples directly relevant to the point raised here.

Regarding Hanlon et al. (2019, *Evol. Lett.*), we agree that this study is relevant background for genetic processes in long-lived conifers, especially the accumulation of somatic mutations. However, it is less directly aligned with the specific "reservoir" question addressed here.

We therefore agree that the prior literature should be acknowledged more carefully, and we have revised the framing accordingly. This helps position our main conclusion more precisely: in our study system, *Glyptostrobus pensilis*, the genomic importance of ancient trees is heterogeneous among individuals and is better explained by population history than by age alone.

2. Lack of direct age estimation
The ages of the 147 sampled trees are not directly quantified. Instead, diameter at breast height (DBH) is used as a proxy to distinguish ancient and wild trees. Although Tang et al. (ref. 18) reported a positive relationship between DBH and age, this relationship is based on a case study from Fujian Province and may not be broadly applicable. Given that G. pensilis is a relatively fast-growing species, large individuals in other regions may still be relatively young. As a result, conclusions related to individual age are not well supported. More importantly, the study conflates conservation categories (ancient, wild, cultivated) with tree age. To directly test the central hypothesis, it would be preferable to incorporate dendrochronological data or other reliable age estimates and explicitly analyses the relationship between individual age and genomic diversity.

Answer: We appreciate this important comment. In the revised manuscript, we now make this limitation much clearer and provide substantially fuller age-related information for all 147 sampled individuals to support the interpretation of the three conservation types. The corresponding revisions are included in the Results (Lines 108–117), Methods (Lines 421–438), and Supplementary Table 3.

First, our study was not designed to quantify exact chronological age for every sampled tree, but to compare three independently defined, non-overlapping conservation types: ancient, wild, and cultivated trees, which differ in broad age structure, ecological setting, and management background. In this study, ancient trees are defined as officially documented individuals older than 100 years occurring in

human-dominated landscapes, whereas wild individuals occur in remnant natural swamp habitats and recently cultivated individuals represent trees from recent plantings (typically 20–50 years old). In addition, direct age determination was not practical in our study system, because *G. pensilis* is critically endangered and legally protected in both China and Vietnam, making invasive coring or ring counting of the limited remaining ancient and wild individuals unrealistic.

Second, we have substantially expanded the age-related information for all 147 sampled individuals to support the interpretation of broad age-structure differences among the three conservation types. All sampled 64 ancient trees are supported by available records as being older than 100 years, and most recorded ages fall between 150 and 800 years after excluding the two lowest and two highest recorded values. These records derive from multiple documentary sources, including published studies, forestry records, archival documentation, news reports, and local interviews. To provide broader age-related context for the non-ancient groups, we now summarize the available evidence from the main sampled wild-population regions. Of the 33 wild individuals, 22 came from Fujian and 3 from Vietnam, where published mean ages are ~85 years (Tang et al. 2019, *Plant Divers.*) and ~112 years (Averyanov et al. 2009, *Taiwania*), respectively. The remaining 8 wild individuals came from a small population in Guangdong, where even the largest reported tree is unlikely to exceed ~70 years. For the cultivated type, the 50 sampled individuals were mostly recent plantings (~20–50 years old), based on planting histories, local records, and information from site managers.

Third, we have revised the treatment of DBH so that it is no longer presented as a basis for assigning ancient status, but only as supporting size-related information. Tang et al. (2019, *Plant Divers.*) showed that radial growth in wild G. pensilis declines markedly with age, from about 7 mm yr^{-1} in trees younger than 60 years to about 0.89 mm yr^{-1} in trees older than 100 years. Thus, although DBH should not be used as a direct age estimator because growth can vary with geography, climate, and human management, slower DBH accumulation in older trees is still biologically expected in this species. In our dataset, DBH differed in the same general direction among the three conservation types (median: 89.2 cm in ancient, 49.4 cm in wild, and 31.1 cm in cultivated trees; Fig. 1d and Supplementary Table 1), providing supporting size-related evidence consistent with the age-related patterns summarized above.

Finally, our revised framing is that the available evidence supports broad age-structure differences among ancient, wild, and cultivated trees, while the genomic analyses evaluate whether ancient-tree status, as defined in this conservation context, is associated with distinctive genomic patterns. We consider this the most appropriate interpretation for the comparisons undertaken here, given both the biology of the species and the practical constraints on direct age estimation.

3. Interpretation of population history versus age effects
I agree that population history is likely a major driver of genomic diversity. However, this conclusion would be more convincing if the authors compared individuals with similar population histories but different ages within the same region. Such a design would allow a more direct test of the relative importance of age versus lineage.

Answer: Thank you for this helpful suggestion. We agree that comparisons within lineages help clarify the relative contributions of age and population history. In the revised manuscript, we have therefore added a short paragraph in the Results (Lines 273–280) and two new Supplementary Figs. 16 and 17, showing comparisons of ancient, wild, and cultivated individuals within the two major lineages, Zhujiang and Minjiang, each of which is largely restricted to a single geographic region.

The revised text now states (Lines 273–280): "Weak differentiation among conservation types was also observed within lineages. In the Minjiang lineage, ancient, wild and cultivated individuals showed broadly overlapping nucleotide diversity, heterozygosity, inbreeding (F_{ROH}), selection efficacy ($\pi0/\pi4$), and mutation load metrics, without a consistent pattern of significant differences across these variables (Supplementary Fig. 16). A similar genomic pattern was observed in the Zhujiang lineage, with minor deviations involving wild individuals (Supplementary Fig. 17), likely reflecting their small sample size and origin from a single geographic population.".

We have also incorporated this within-lineage perspective into the revised Discussion (Lines 345–347).

4. Limitations of species choice and sample size
The relatively small sample size may limit the robustness of the conclusions. In addition, *G. pensilis* rarely exceeds ~150 years (ref. 18), meaning that the sampled "old" trees may not represent truly long-lived individuals. Extremely old trees (e.g., >500 years) are often survivors from historically large and genetically diverse populations and may represent "lottery winners" (ref 2). In contrast, *G. pensilis* populations have been strongly impacted by human activities and may largely consist of younger cohorts. Therefore, the observed patterns could be strongly influenced by population history and genetic drift, rather than providing a definitive test of whether very old trees harbor unique genetic variation.

Given these limitations, the conservation implications should be framed more cautiously.

Answer: Thank you for this important comment. Our ancient-tree sample was not intended as a small arbitrary subset, but to include most known surviving ancient trees where sampling was feasible, while the overall sampling of 64 ancient, 33 wild, and 50 cultivated individuals was designed to capture the extant diversity of the three conservation types across the current range of *G. pensilis*. Based on the available records, all 64 sampled ancient trees exceed 100 years, 62 of 64 exceed 150 years, and

24 of 64 (37.5%) exceed 500 years (Fig. 1c and Supplementary Table 3). These data support the view that the sampled ancient trees represent genuinely old individuals spanning a broad age range, and they also support broad age-structure differences among the ancient, wild, and cultivated categories. We now provide substantially fuller age-related information for these sampled ancient trees in the Results (Lines 108–117), Methods (Lines 421–439), and Supplementary Table 3.

At the same time, we agree that these genuinely old individuals occur in a species whose recent history has been strongly shaped by demographic contraction, drift, and human influence, and that these factors can strongly affect the observed patterns. We now make this much more explicit in the revised manuscript. Accordingly, we no longer present *G. pensilis* as a definitive general test of whether very old trees harbor unique genetic variation across all long-lived tree systems. Instead, we emphasize that, in this endangered conifer, ancient-tree status alone did not consistently predict greater overall genomic variation than younger wild or cultivated trees. Much of the observed heterogeneity is better explained by lineage history and provenance. In this sense, the value of this system is not that it provides a universal answer, but that it offers a realistic and conservation-relevant example in which ancient status, demographic history, drift, and human influence all interact.

We also think that related logic may extend beyond highly endangered species, although this will require direct testing. Even in more widespread taxa that may better exemplify the idea that ancient or old-growth trees can act as reservoirs of beneficial alleles, larger effective population sizes would not necessarily erase lineage structure. Where regeneration and gene flow are more extensive, variation carried by old trees may also be more readily transmitted to and retained in extant populations. Under such conditions, the evolutionary importance of old individuals may still depend less on age itself than on whether they retain lineage-specific or otherwise underrepresented genomic variation. Nevertheless, given the limitations inherent to this endangered and strongly bottlenecked study system, we have tempered the Abstract (Lines 32–34), Introduction (Lines 95–97), and Discussion (Lines 367–371 and 390–395) accordingly, so that our conclusions are framed more explicitly as being drawn from *G. pensilis*.

Minor comments

Lines 91–112: The section describing poorer vitality in ancient trees is not directly relevant to the main research question and could be substantially shortened.

Answer: We have shortened and refocused this section accordingly. The revised Results (Lines 99–132) now place greater emphasis on age-related information for the ancient, wild, and cultivated categories, while the poorer condition of ancient trees is mentioned only briefly to indicate their greater current conservation urgency.

Lines 370–371: The information presented appears to be based on anecdotal sources and may not be reliable; it should be revised or removed.

Answer: We agree and have removed this information from the main text. We now retain this example only in Supplementary Note 1 and Supplementary Fig. 21 as an illustrative case of a formally protected ancient *G. pensilis* individual with on-site conservation signage.
